# Genetic Polymorphisms and Weight Loss in Obesity: A Randomised Trial of Hypo-Energetic High- versus Low-Fat Diets 

**DOI:** 10.1371/journal.pctr.0010012

**Published:** 2006-06-30

**Authors:** Thorkild I. A Sørensen, Philippe Boutin, Moira A Taylor, Lesli H Larsen, Camilla Verdich, Liselotte Petersen, Claus Holst, Søren M Echwald, Christian Dina, Søren Toubro, Martin Petersen, Jan Polak, Karine Clément, J. Alfredo Martínez, Dominique Langin, Jean-Michel Oppert, Vladimir Stich, Ian Macdonald, Peter Arner, Wim H. M Saris, Oluf Pedersen, Arne Astrup, Philippe Froguel, The NUGENOB Consortium

**Affiliations:** 1 Institute of Preventive Medicine, Danish Epidemiology Science Centre, Copenhagen University Hospital, Copenhagen, Denmark; 2 CNRS UPRES A 8090, Institut Biologie de Lille, Institut Pasteur de Lille, Lille, France; 3 School of Biomedical Sciences, Queen's Medical Centre, University of Nottingham Medical School, Nottingham, United Kingdom; 4 Steno Diabetes Centre, Gentofte, Denmark; 5 Department of Human Nutrition, Centre for Advanced Food Research, The Royal Veterinary and Agricultural University, Copenhagen, Denmark; 6 Department of Sports Medicine, Centre of Preventive Medicine, Third Faculty of Medicine, Charles University, Praha, Czech Republic; 7 Department of Nutrition, Hôtel-Dieu Hospital, University Pierre-et-Marie Curie (Paris 6), Paris, France; 8 Department of Physiology and Nutrition, University of Navarra, Pamplona, Spain; 9 Obesity Research Unit, INSERM U586, Louis Bugnard Institute and Clinical Investigation Centre, Toulouse University Hospitals, Paul Sabatier University, Toulouse, France; 10 The Obesity Unit, Department of Medicine, Karolinska Institute, Huddinge University Hospital, Stockholm, Sweden; 11 Department of Human Biology, Nutrition, and Toxicology Research Centre NUTRIM, Maastricht University, Maastricht, Netherlands

## Abstract

**Objectives:**

To study if genes with common single nucleotide polymorphisms (SNPs) associated with obesity-related phenotypes influence weight loss (WL) in obese individuals treated by a hypo-energetic low-fat or high-fat diet.

**Design:**

Randomised, parallel, two-arm, open-label multi-centre trial.

**Setting:**

Eight clinical centres in seven European countries.

**Participants:**

771 obese adult individuals.

**Interventions:**

10-wk dietary intervention to hypo-energetic (−600 kcal/d) diets with a targeted fat energy of 20%–25% or 40%–45%, completed in 648 participants.

**Outcome Measures:**

WL during the 10 wk in relation to genotypes of 42 SNPs in 26 candidate genes, probably associated with hypothalamic regulation of appetite, efficiency of energy expenditure, regulation of adipocyte differentiation and function, lipid and glucose metabolism, or production of adipocytokines, determined in 642 participants.

**Results:**

Compared with the noncarriers of each of the SNPs, and after adjusting for gender, age, baseline weight and centre, heterozygotes showed WL differences that ranged from −0.6 to 0.8 kg, and homozygotes, from −0.7 to 3.1 kg. Genotype-dependent additional WL on low-fat diet ranged from 1.9 to −1.6 kg in heterozygotes, and from 3.8 kg to −2.1 kg in homozygotes relative to the noncarriers. Considering the multiple testing conducted, none of the associations was statistically significant.

**Conclusions:**

Polymorphisms in a panel of obesity-related candidate genes play a minor role, if any, in modulating weight changes induced by a moderate hypo-energetic low-fat or high-fat diet.

## Editorial Commentary

**Background:** Obesity is an important cause of death and disease, particularly in the developed world. It is understood that both environmental and genetic factors contribute towards obesity. Numerous studies have associated particular gene variants with a tendency towards obesity, but it is not known whether such gene variants affect the degree to which obese individuals will lose weight when dieting.

**What this trial shows:** As part of a randomised trial, 771 participants were assigned to one of two different low-energy diets for 10 weeks: one low in fat or one high in fat. The researchers then did a genetic analysis of 642 participants completing the intervention, to find out whether any of 42 distinct genetic variations in 26 genes were associated with weight loss in the trial. The genetic variants were chosen for study as they were known or already thought to be associated with appetite regulation or various aspects of metabolism and fat tissue development and function. The investigators found that none of the genetic variants studied had a significant association with weight loss in the trial. It was also seen that the majority of genetic variants were not associated with efficacy of one dietary intervention over another.

**Strengths and limitations:** Although a large number of participants was recruited into the trial, the genetic analysis involved multiple comparisons—168 tests of 42 genetic variants. This increases the likelihood that any significant associations found could have resulted from chance alone. Significant associations from this study will require additional confirmation in larger studies.

**Contribution to the evidence:** This study adds data indicating that variation in the genes studied did not have an important influence on weight loss.

## INTRODUCTION

Obesity has a joint genetic and environmental etiology [1]. Rare cases of severe, early onset obesity have been attributed to single gene mutations with large functional effect [2]. The genetic influence on obesity may be because of combinations of multiple genes with individual small effect sizes related to frequent single nucleotide polymorphisms (SNPs). These may interact mutually and with the environment in a complex manner to influence obesity [2,3]. Numerous gene variants have been reported to be associated with obesity or obesity-related phenotypes [4], but only a few of the associations have been replicated in other populations [2,4].

It is conceivable that the effect of some genes on the obesity phenotypes may be nutrient-sensitive. Indeed, diet composition and/or energy intake may modulate gene expression through complex transcriptional mechanisms as well as more downstream processes involving the gene products [2,3]. Several studies have clearly demonstrated that expression of multiple genes in adipose tissue is altered by reductions in energy intake, but so far none has revealed an influence of the dietary composition in this setting [3,5,6]. The implications for human health of alterations in these processes by common genetic polymorphisms are still elusive, but they may be revealed by showing that the effect of the diet on weight changes depends on differences in the genes, and this could also help explain discrepancies in the association studies.

However, few gene-nutrient interaction studies of obesity and weight change have been carried out in humans, and the results have been ambiguous, whether being based on either epidemiological, observational, and cross-sectional studies [3,7] or small experimental over-feeding studies [8]. A few studies [3], including twin studies [9], suggest that genes play a role in weight loss (WL) as the outcome of dietary treatment of obesity. These genes could be the same genes as those involved in obesity, exemplified by a study of the relation between variation in the *MC4R* gene, the most common form of monogenic obesity [2,10], and the outcome of surgical treatment [11]. However, twin studies of the two phenotypes—degree of obesity and WL—show that the genetic correlation for these phenotypes is far from 1.0, implying that it is only a subset of the genes that are the same behind the genetic influence on either of the two phenotypes [12].

A possibly useful approach to elucidate the role of the genes and diets in obesity would be to study effects of genetic polymorphisms on WL induced by specific dietary treatments of obesity. The NUGENOB Consortium (Nutrient-Gene Interactions in Human Obesity: Implications for Dietary Guidelines) has conducted a randomised intervention trial of a 10-wk low-fat or high-fat hypo-energetic diet (with fixed protein content and a corresponding high or low carbohydrate content) in obese individuals from eight centres in seven European countries with the specific purpose of studying aspects of gene-nutrient interaction (for more on the NUGENOB project, see http://www.nugenob.org) [13]*.* This trial showed that the low-fat diet produced the same mean WL as the high-fat diet, but resulted in more participants losing >10% of initial body weight and had fewer dropouts [13].

The aim of the present study was to investigate, using the NUGENOB trial [13], if genetic polymorphism modulates WL induced by either a low- or a high-fat hypo-energetic diet. Common SNPs in a panel of obesity-related candidate genes were selected on the basis of their known or presumed involvement in various parts of the pathogenic processes of obesity and its related phenotypes.

## METHODS

The study was a randomised, parallel, two-arm, open-label, 10-wk intervention by two hypo-energetic diets with either low or high fat content, undertaken at eight sites in seven European countries. The clinical parts of the trial are described in detail in a previous publication [13].

### Participants and Baseline Investigation

We planned to recruit 100 Caucasian Europeans from each of seven centres and 50 from one other centre, and included during the period May 2001 through September 2002 in total 771 participants (579 women) from the United Kingdom (Nottingham), the Netherlands (Maastricht), France (Paris and Toulouse), Spain (Pamplona), Czech Republic (Prague), Sweden (Stockholm), and Denmark (Copenhagen).

The study aimed at recruiting participants with a body mass index (BMI) greater than or equal to 30 kg/m^2^ and age 20–50 y, and without weight change more than 3 kg within the 3 mo prior to the study start. Participants reporting clinically diagnosed hypertension, diabetes or hyperlipidæmia treated by drugs, untreated thyroid disease, surgically or drug-treated obesity, pregnancy, or alcohol or drug abuse were excluded. Participants not included in other trials were recruited through local sources as available and convenient. The ethical committee at each of the participating centres approved the study. Volunteers were informed about the nature of the study, and written consent was obtained prior to study participation.

Before assignment to the dietary intervention group, all participants underwent a thorough standardised clinical and physiological examination described in Standard Operational Procedures applied in all participating centres (see Trial Protocol and Annexes). Each examination included blood sampling, and measurement of height with a calibrated stadiometer and weight (in light indoor clothes and without shoes) with a calibrated set of scales. Resting metabolic rate was measured by a ventilated hood system.

Blood drawing and processing of blood samples were performed according to international guidelines for genetic studies [14]. Whole blood was drawn in sterile vacutainer tubes with ethylenediaminetetraacetic acid. The plasma was removed, and the white cell layer (the buffy coat) was transferred to cryovials and frozen at −80 °C.

### Interventions

The target macronutrient composition of the two diets was: *low-fat diet*: 20%–25% of total energy from fat, 15% from protein and 60%–65% from carbohydrate; *high-fat diet*: 40%–45% of total energy from fat, 15% from protein, and 40%–45% from carbohydrate. Both diets were designed to provide 600 kcal/d fewer than the individually estimated energy requirement, which was based on pre-treatment resting metabolic rate multiplied by a physical activity level of 1.3, assuming a sedentary life style. The dietary programme is described in detail on the Web site http://www.nugenob.org. The dietary instructions also aimed at minimising differences between the two diets in other components such as source and type of fat, amount and type of fibre, type of carbohydrate, amount of fruit and vegetables, and in meal frequency patterns, while taking local customs into account as appropriate. Participants were requested to abstain from alcohol consumption. The dietary instructions were reinforced and monitored and participants were weighed weekly. Participants were advised to follow their habitual activity patterns throughout the dietary intervention period. A 3-d, weighed food record of two weekdays and one weekend day was obtained before the study and during the last week of the intervention. One-day weighed food records were completed during the second, fifth, and seventh weeks. The dietary records were analysed using the country-specific food-nutrient database routinely used in each centre. The nutrient composition of the baseline diet was the average across the 3d of recordings, and the composition of the intervention diet was based on averaging across all the dietary recordings during the intervention period (Table 1).

**Table 1:**
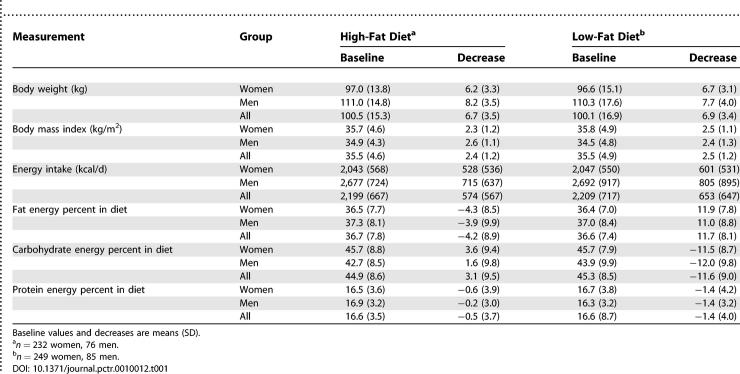
Baseline and 10-wk Measurements of Body Weight, Body Mass Index, Energy Intake, and Macronutrient Energy Percent of the Diet (without Alcohol Energy Percent) in 642 European Obese Individuals Who Completed Dietary Intervention

### Objectives

The objectives of the clinical trial were to assess the effect on WL in obese participant of a low-fat versus a high-fat hypo-energetic diet. The objectives of the present analysis were to investigate if the genotypes of 42 SNPs in 26 candidate genes, probably associated with hypothalamic regulation of appetite, efficiency of energy expenditure, regulation of adipocyte differentiation and function, lipid and glucose metabolism, or production of adipocytokines, influenced the WL, possibly dependent on which of the two diets the participants were prescribed.

### Outcomes

The outcomes in the present analysis were the WL during the 10-wk dietary intervention in the various genotype groups defined by the SNPs in the selected genes.

The selection of candidate genes was based on their potential contribution to obesity-related phenotypes, their presumed nutrient-sensitive expression and function of the gene products, and the presence of common SNPs with a presumed allele frequency above 0.05 allowing meaningful analysis of the interaction between diet and its effect on WL. The SNPs were selected through mutation analysis of genes found by positional cloning, mutation analysis of plausible biological candidate genes selected from the literature and found to be associated with functional effects of the gene products, and the available pertinent knowledge about already known obesity-related SNPs [2].

On this basis, 42 SNPs in 26 genes known or presumed to be associated with hypothalamic regulation of appetite, efficiency of energy expenditure, regulation of adipocyte differentiation and function, lipid and glucose metabolism, or production of several adipocytokines were selected [15–54]. Table 2 lists the genes and the SNPs analysed, and they are grouped according to their presumed function in relation to the obesity phenotypes. It appears from the positions of the SNPs that some may have functional effects by changing the gene products, some may affect the promoter function, and others may be in linkage disequilibrium with other SNPs with functional effects. Since all selected SNPs have been found in previous studies to be associated with obesity-related phenotypes, they may be considered tag SNPs of potentially functional haplotypes irrespective of their own presumed functional effects in relation to the obesity-related phenotypes or the currently addressed induced WL. For several of the genes selected (*ENPP1, GAD2,* *CART, SLC6A14, GHRL, PCSK1, ADIPOQ,* and *WAC*) a linkage disequilibrium analysis secured an optimal coverage of the genetic variation known at the time of gene selection. For this reason, these SNPs were considered the risk SNPs, and the participants not carrying these SNPs were denoted “noncarriers” corresponding to “wild types” irrespective of which alleles were most frequent in the present study.

**Table 2:**
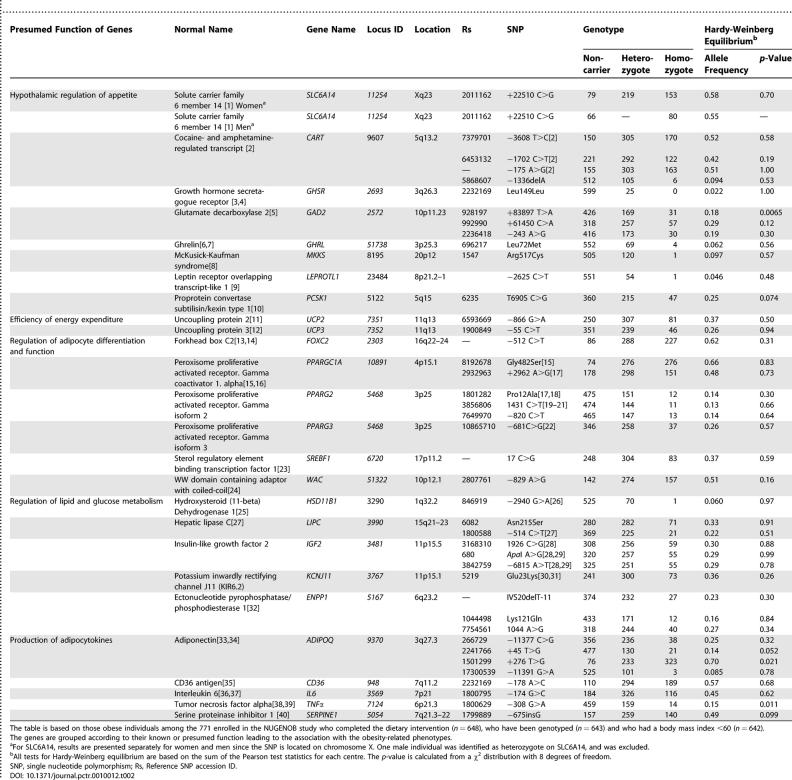
Genotype Distribution and Allele Frequencies in 642 European Obese Women and Men Enrolled in the NUGENOB Study

For each genotype, four genetic models were analysed. In the first general genetic model, there were no assumptions made about a specific effect of the mutant alleles in the individuals who were heterozygous and homozygous for the gene variant when compared with those who were noncarrier (null hypothesis). Three specific genetic models assume a particular effect of the gene variant compared with the noncarrier: dominant effect (noncarrier = 0, heterozygous and homozygous = 1), co-dominant effect (assessed as an additive effect: noncarrier = 0, heterozygous = 1, homozygous = 2), and recessive effect of gene variant (noncarrier = 0, heterozygous = 0, homozygous = 1). For some of the variants, the homozygotes were so rare (less than 10 homozygotes in 7 out of the 42 SNPs) that identification of recessive transmission or distinction between dominant and co-dominant transmission would not be possible (Table 2).

### Genotyping

Samples of buffy coat, drawn at baseline, were sent to the Steno Diabetes Centre in Copenhagen on dry ice, where DNA was extracted. Extracted DNA samples were diluted in Tris/EDTA buffer to a stock DNA solution of 100 ng/μl and a working DNA solution of 10 ng/μl. Stock solutions were stored at −80 °C, working solutions were stored at 4 °C. DNA samples were stored and handled in locations free of contaminating polymerase chain reaction products.

Forty of the 42 SNPs in the 26 genes were genotyped by the LightCycler assay (Roche Diagnostics, Basel, Switzerland) (the *SLC6A14, CART, GHSR, GAD2, GHRL, LEPROTL1, UCP2, UCP3, WAC, LIPC, IGF2, ENPP1, ADIPOQ,* and *CD36* genes) based on hybridization probes labelled with fluorescent dyes that allow fluorescence resonance energy transfer [55] or by TaqMan assay (Applied Biosystems, Foster City, California, United States) (the *MKKS, PCSK1, FOXC2, PPARGC1A, PPARG2, PPARG3, SREBF1, HSD11B1, KCNJ11, IL6, TNFα,* and *SERPINE1* genes). Sequences of primers pairs, labelled with fluorescein and LC Red 640 are available on request from the authors. One SNP in *IGF2* (−6815 A>T) and one in *CART* (−3608 T>C) were genotyped by direct sequencing. No genotyping could be carried out in seven out of the 771 participants enrolled in the study, and in five out of those completing the intervention. The genotyping success rate was 92.8%–98.8% except for *WAC* (89.2%) and *SERPINE1* (86.6%).

### Sample Size

For a single analysis, the least detectable effect (WL, in kg) was estimated by the programme QUANTO version 1.0 (see http://hydra.usc.edu/gxe) with the assumptions of a statistical power of 0.80, a sample size of 642 individuals, and a mean WL of 6.8 kg with a SD of 3.5 kg. For allele frequencies ranging from 0.05–0.50, the least detectable WL of main effects of genes ranged from 1.30–0.78 kg (0.89 kg for an allele frequency of 0.50) for dominant models, from 7.72–0.89 kg for recessive models, and from 1.25–0.55 kg for additive co-dominant models. Assuming, in addition, equal distribution of participants between the diets, the least detectable effects on WL of gene-diet interaction, modelled as specified above, ranged from 2.59–1.54 kg (1.77 kg for allele frequency of 0.50) for dominant models, from 15.5–1.77 kg for recessive models, and from 2.49–1.07 kg for additive co-dominant models.

When assuming that testing for each SNP in each genetic model is a testing of the same null hypothesis multiple times, the needed sample size increases. Thus, as an example, detection of a significant difference of 1.5 kg between the low-fat and high-fat diets in heterozygotes and a difference of 3.0 kg between the diets in homozygotes versus that observed in noncarriers at an alpha level of 0.05, and a statistical power of 0.80 with four genetic models for 42 SNPs (168 tests of the same null hypothesis) would require sample sizes ranging from 5,729 through 1,008 participants in the trial for allele frequencies ranging from 0.05 through 0.50, respectively.

### Randomisation

Stratified block randomisation was used with centre, gender, and three age groups (20–29 y, 30–39 y and 40–50 y) as strata and a block size of 12. The randomisation list was computer-generated and the block size was unknown to the clinical centres. Individual randomisation was carried out at the co-ordinating centre following transfer from the clinical centres of the relevant baseline information on the participants.

### Statistical Methods

Examination of Hardy-Weinberg equilibrium was carried out by applying a global test for all genotypes (using the HWE.GENEPOP programme) [56], and a test for each genotype by taking into account centre differences by summing up Pearson χ^2^ statistics for each centre and comparing with a χ^2^ distribution with 8 degrees of freedom. Genotype distributions in various subgroups (eligible versus noneligible, women versus men, randomised diet groups, and completers versus noncompleters of the intervention) were compared by the Pearson χ^2^ statistics as appropriate. The primary outcome in the present analysis was mean WL (in kg) with 95% confidence intervals (CI), conditional on genotype and assigned diet. The WL was calculated as the difference between weight recorded immediately before randomisation and the weight at the completion of the intervention programme. Differences in body weight changes were compared by regression models, in which we controlled for centre (Gaussian random effect), gender, age (linear and squared), and baseline weight (linear) separately for men and women. The distributions of the residuals were compatible with the normal distribution and this was verified by the Shapiro-Wilk test.

The regression model addressing the effects of the genotypes on WL—main effects assessed in one model for each genotype—included the genotypes for each variant as covariates and as a separate covariate, the diet group to which the participants had been randomised. The gene–diet interaction analysis was carried out by estimating for each genotype the difference in WL, adjusted by the regression model as described, between the low-fat group (lf) and the high-fat (hf) group, and then comparing the differences in WL by diet group for a particular genetic variant (ga) and the noncarrier (gn); using the indicated notation, the outcome variable expressing the interaction would be derived from this equation: [WL(lf, ga) – WL (hf, ga)] – [WL(lf, gn) – WL(hf, gn)]. Hence, a positive value of an estimated WL difference indicates a greater WL on the low-fat diet than on the high-fat diet in individuals carrying the particular gene variant than in noncarriers of the variant.

For all the genes in which genotyping was carried out for more than one SNP (see Table 2), haplotype-based analysis was carried out by the programme GENECOUNTING (version 1.3) [57], which implements an Expectation Maximisation algorithm for estimation of the haplotype probabilities in unrelated participants without reference to the phenotype. Subsequently, we estimated the relation between the possible haplotypes on each chromosome and WL by using haplotype pairs for each participant as covariates in regression models with WL as outcome. This was carried out by multiple imputations. In each of 1,000 imputations, a new dataset was generated where the haplotype pairs for each participant were drawn at random according to the haplotype probabilities for all the possible haplotypes for each individual as estimated by the GENECOUNTING programme. The imputed datasets are each analysed by the same univariate model; the WL differences and its variances were estimated based on the method of imputation, and a test for departure from no association was constructed [58]. This approach exploits the entire information on possible haplotypes for each participant in contrast to conventional methods selecting for each individual only the most probable haplotype, implying that all other less likely haplotypes are discarded.

The statistical significance was set at *p* < 0.05, but as mentioned above, the interpretation of the significance level must take into account that testing is performed for two different types of hypotheses (gene effects on WL as such, and gene × diet interaction), four different types of genetic models (unspecified, dominant, co-dominant, and recessive) and 42 different SNPs (i.e., in 2 × 168 = 336 partially dependent analyses). Although no adequate formal correction for multiple testing is available for this scenario, any apparently statistically significant outcome of single tests must be considered tentative, requiring independent replication in other studies.

The statistical software SPSS version 11.5 (SPSS, Chicago, Illinois, United States); SAS version 8.2 (SAS Institute, Cary, North Carolina, United States); and STATA version 8.0 (Stata, College Station, Texas, United States), were used as appropriate.

## RESULTS

### Patient Flow and Numbers Analysed

The outcome of the dietary intervention trial has been reported in detail elsewhere [13]. Out of the 771 obese individuals enrolled in the trial, 648 completed the dietary intervention, and genotype data were available in 643 of these participants because of lack of DNA or genotyping failure in 5 participants.

**Figure 1:**
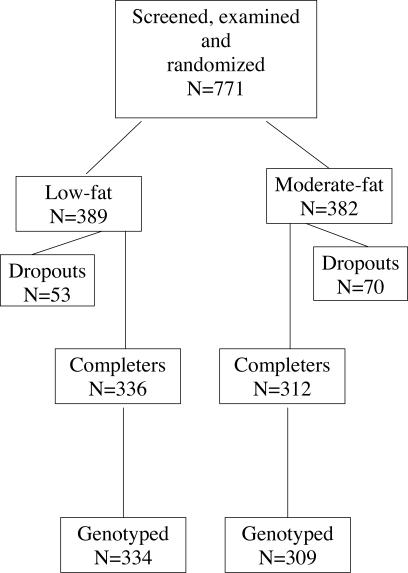
Patient Flow in the Trial

In this group, 129 individuals (20.1%) exhibited characteristics deviating from the recruitment criteria, predominantly because of lower baseline BMI (range 26.4–29.9, mean 29.2) in 35 individuals (5.5%) or weight instability during the last 3 mo before enrolment in 73 (11.4%), but they were all kept in the present analysis because there were no prior reasons to assume that this would affect the results of the analyses. One participant was considered an outlier by having a combination of an excessively great BMI (66.1 kg/m^2^; all others were below or equal to 56 kg/m^2^) and increase in body weight during the intervention, which affected the underlying basic regression model. Therefore, the present analyses were based on 642 individuals. The relation between the originally recruited 771 participants and the distribution on the randomised groups are shown in Figure 1.

### Baseline Data

There were only minor differences in the distributions of genotypes between this group and those who did not complete the intervention (unpublished data). The distribution of the genotypes on noncarriers, heterozygotes and homozygotes, the allele frequencies, and the outcome of testing for Hardy-Weinberg equilibrium are shown in Table 2. All allele frequencies exceeded 0.05 except for *GSHR* −447 C>G, which, however, was retained in the analysis. As expected, it appears that the frequencies of homozygotes for a number of alleles were so small that analysis of associations with specific genetic models would be impossible or very uncertain as also indicated in the estimated least detectable effects above. The global test for Hardy-Weinberg equilibrium of all genotypes showed no significant departures within each centre. As seen in Table 2, the test for each individual genotype showed that all SNPs barring three (*GAD2*, +83897 T>A; *ADIPOQ*, +276 T>G; and *TNFα*, −308 G>A) were in Hardy-Weinberg equilibrium, but in view of the unsuspicious and minor departure from the equilibrium, these SNPs were kept in the analysis. The genotype distributions showed no or small differences (none of which was statistically significant after appropriate adjustment for multiple testing), between men and women, between the two diet groups, or between the (in retrospect) eligible and noneligible groups (unpublished data), and it was therefore not considered necessary when testing each of the genotypes to adjust for the other genotypes.

Total energy intake and dietary fat energy percent during the interventions were within the targeted intervals (Table 1). Mean WL was 6.9 kg in the low-fat group, and 6.7 kg in the high-fat group, with no significant group difference (mean 0.3 [95% CI, −0.3 to 0.8] kg), and this corresponds closely to the WL expected from the dietary energy restriction. The two diet groups did not differ in baseline and decrease in fat body mass, lean body mass, waist circumference, hip circumference and fasting insulin, nor in glucose levels [13]. The blood-lipid profiles differed as expected from the differences in diet composition (the low-fat diet group had greater decline in plasma cholesterol, plasma low-density lipoprotein cholesterol, and plasma high-density lipoprotein cholesterol, and less decline in plasma triacylglycerol), which supports that the dietary intervention did achieve the desired difference in the diet consumed [13]. There was, however, a considerable unexplained interindividual variation in WL within both groups, with standard deviations of 3.4 kg and 3.5 kg in the low-fat and high-fat groups, respectively (Table 1), which could be caused by genetic differences.

### Outcomes and Estimation

#### Genetic effects on WL.

Table 3 shows the results of the analysis of effects of genotypes on WL while controlling for the diet group (main effects of genotypes) without specifying genetic models. The table presents the difference in WL between the groups of participants who are heterozygous and homozygous for the risk SNPs and those who are homozygous noncarriers of the risk alleles, which thus is the reference group and therefore not depicted. None of the genotypes had statistically significant influence on WL in general. The effects in heterozygotes, compared with noncarriers, ranged from −0.6 to 0.8 kg, and all the CI's overlapped or included 0.0 kg. The effects in homozygotes ranged from −0.7 to 3.1 kg, and all CIs overlapped 0.0 kg. All analyses of the general effects of the haplotypes on WL were nonsignificant (unpublished data), except for those of *ENPP1* (*p* = 0.04), see below.

**Table 3:**
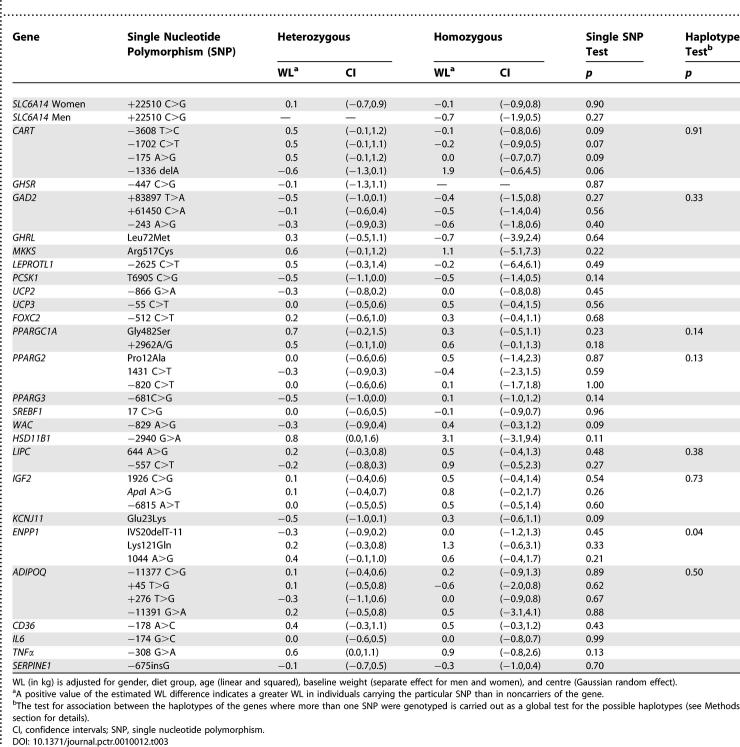
Differences in Weight Loss (WL) by Genotypes (with the Noncarrier Reference) following 10-wk Dietary Intervention, Aiming at Reducing the Energy Intake by 600 kcal in 642 European Obese Women and Men

#### Gene-diet interaction effects on WL.

Table 4 shows the outcome of the analyses of effects of gene-diet interaction on WL, i.e., the possible effects of genotype on the efficacy of the particular dietary intervention. If there were no interaction between the particular genotype and the dietary intervention, then the differences in WL between the dietary groups for each genotype would be the same, and the figures in the table would be 0.0 kg, whereas a value for the estimated WL significantly departing from 0.0 kg would indicate a gene-diet interaction. Positive figures mean that the WL is greater on the low-fat than with the high-fat diet when the risk SNP is present than when it is not present (noncarrier), and vice-versa for negative figures. As in Table 3, those who are noncarriers of the risk SNPs are the references groups and thus not depicted.

**Table 4:**
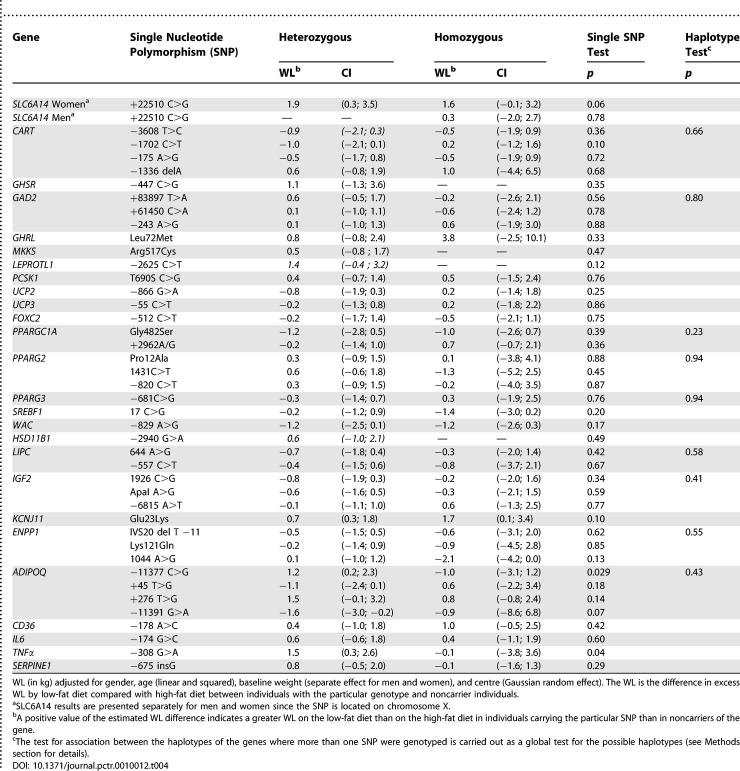
Effects of Genotype–Diet Interactions on Weight Loss (WL) following 10-week Dietary Intervention, Aiming at Reducing the Energy Intake by 600 kcal Either by a Low-Fat or a High-Fat Diet Allocated at Random to 642 European Obese Women and Men

The genotype-dependent excess WL on low-fat diet compared with high-fat diet ranged from 1.9 to −1.6 kg in heterozygotes, and from 3.8 kg to −2.1 kg in homozygotes, but, except for association with one SNP in *ADIPOQ* (−11377 C>G) and the SNP of the *TNFα*, none were significant. These two test results would not be statistically significant if the multiple testing related to Table 4 was taken into account.

None of the haplotype analyses showed any overall statistically significant effects, and judged from the CI, none of the individual haplotypes was statistically significant.

### Ancillary Analyses

#### Specific genetic models and specific haplotypes.

In single tests significant association (*p* < 0.05) with WL was found for the SNPs of the genes *PCSK1, WAC*, *HSD11B1,* and *TNFα*, when assuming specific genetic models, and these findings are summarised in Table 5.

**Table 5:**
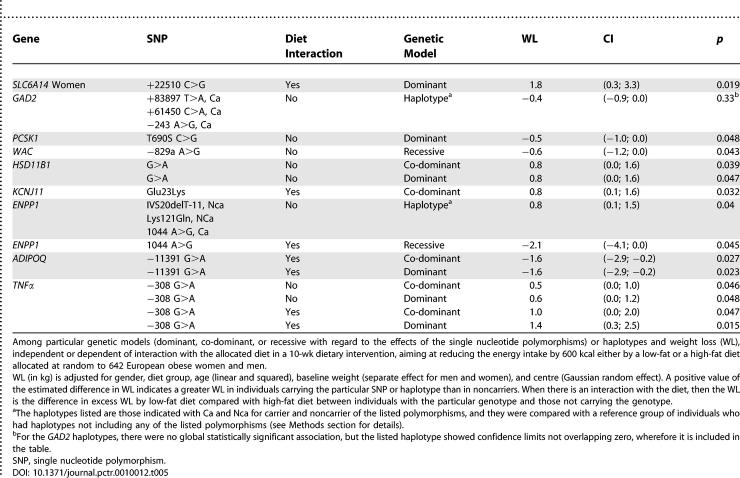
Statistically Significant Associations (without Adjustment for Multiple Testing Effects)

For the *GAD2* gene, the global analysis of the haplotypes was not significant, but there was one haplotype, composed of a combination of all three risk SNPs, which compared with the haplotype without any risk SNPs, showed a borderline effect according to the CI (Table 5). The effect of the haplotype in which only the *ENPP1* SNP 1044 A>G was present with the other loci without the risk SNPs appeared to be the main contributor to the significant effect compared with the haplotype including none of the risk SNPs (Table 5).

When assuming specific genetic models in the analyses of the dietary interactions with the genotypes, we found few statistically significant (*p* < 0.05) interaction effects for variants in the *SLC6A14*, *KCNJ11*, *ENPP1* (1044 A>G)*, ADIPOQ* (−11391 G>A) and *TNFα* genes (Table 5).

None of the results shown in Table 5 was adjusted for multiple testing effects, which would have made all results statistically insignificant.

### Adverse Events

As reported previously, there were no adverse events [13]. Dropouts were because of changes in personal circumstances, dislike of the diets, and emerging health problems unrelated to the diets.

## DISCUSSION

### Interpretation

This study aimed at utilising the randomised dietary intervention as an experimental approach to investigate whether the effects of a panel of 42 SNPs in 26 candidate genes are associated with diet-induced WL, and whether these effects are sensitive to the fat (versus the carbohydrate) content of a hypo-energetic diet. All the genes are known or presumed to be associated with obesity-related phenotypes or to be involved in pathophysiological processes implicated in obesity. The overall conclusion from the analyses is that there are no indications in these results of a major influence of any of these genetic polymorphisms on the clinical outcome of the intervention. When considering the several dimensions of multiple testing carried out to address this subject, the few statistically significant results, obtained without any adjustment for the multiple testing, should only be considered as leads to new hypotheses about effects of specific genetic polymorphisms that need to be addressed in subsequent studies. Thus, the study suggests that SNPs in the *PCSK1*, *WAC, HSD11B1,* and *TNFα*, genes and possibly haplotypes of the *GAD2* and *ENPP1* genes may modulate diet-induced weight changes, and that the effect of dietary fat content may depend on SNPs in the *SLC6A14*, *KCNJ11*, *ENPP1, ADIPOQ*, and *TNFα* genes.

The problem of multiple testing of the genotype-phenotype relationships has no unambiguous quantitative solution. On one hand, there are clearly elements of testing of null hypotheses that could not be distinguished on prior reasons that make it unjustifiable to consider each single statistical test independently of all the others. On the other hand, it may not be justified to consider all tests as tests of the same null hypothesis, in which case conventional quantitative adjustment by, for example, the Bonferroni method would be justified. However, the number of statistically significant tests at *p* = 0.05 was about what would be expected under repetitive testing of the same null hypothesis. This suggests that any of the statistically significant findings must be cautiously interpreted and only used as basis for new studies of the possible role of these genetic polymorphisms.

The present study has limitations regarding both the genotypes and the phenotypes. The analysis of SNPs in the candidate genes took place without control of the possibly associated variation in the genomic context. Despite the control for population origin by centre, there may still be population stratification that can produce false effects or obscure the true effects. Moreover, any findings of genotype effects may reflect that the particular SNP is in linkage disequilibrium with other SNPs within the locus or even in the neighbouring chromosomal region, extending beyond the haplotypes of the particular genes tested; and these other SNPs may, in fact, be responsible for the observed effects. The criteria for selection of the SNPs were not based on prior evidence suggesting a specific functional role in dietary-induced WL, which was not available. For several SNPs, the homozygotes were so uncommon that estimates of recessive effects or distinction between dominant and co-dominant effects were impossible, which is also reflected in the width of the CIs. For these SNPs, the analytical focus is on the general nonspecified and/or the dominant genetic models. The speculative possibility that the effect of the SNPs in one gene may depend on genetic sex differences or on the presence of particular SNPs in another gene (gene-gene interactions or epistasis) could not be adequately addressed in the present study, but the genotype distributions in men and women and in the two randomised diet groups were almost similar. Finally, the selection of SNPs in the candidate genes may not provide a comprehensive coverage of the steadily evolving knowledge about the genetic variation in these genes, so we cannot exclude the possibility that other SNPs, not in linkage disequilibrium with those investigated, in some of the genes may have an influence on the clinical outcomes.

Not all individuals enrolled in the NUGENOB trial completed the dietary intervention or strictly met the recruitment criteria, but previous analysis, using imputation of the missing values and sensitivity analysis have shown that the overall results of the two diets on WL are quite robust [13]. Moreover, the genotype distributions were almost similar in these subgroups. We therefore find it unlikely that the inclusion of the participants not fulfilling the recruitment criteria or the restriction of the present study to those completing the dietary intervention has caused any bias in assessment of the role of the genetic variation on WL. On average, targets set for dietary changes were achieved, as also verified by the average WL and the diet-dependent differences in changes in blood lipids [13], but there were considerable individual differences in reported dietary intake, both with regard to energy restriction and dietary composition (see Table 2). It is possible that differences in actual intake have obscured some main effects or interaction effects of the SNPs on the WL, but the likely measurement errors in reported dietary intake prohibit a valid search for such effects. The study period is limited to 10 wk, and the energy restriction was moderate, so it is possible that the more prominent gene-diet interactions may emerge by more severe energy restriction (e.g., by very-low-calorie diets), or during more prolonged intervention and weight maintenance diet. On the other hand, current management strategies for obesity do aim at rather moderate WL as clinically relevant goals [59,60]. Furthermore, it is possible that interactions between genes and diet composition may depend on whether or not there is a concomitant dietary energy restriction. The phenotype analysed is a composite phenotype, and although the average changes in anthropometrics and body composition were similar in the two diet groups [13], the genotype effects on the reduction of adipose tissue mass at various sites (i.e., general versus peripheral and abdominal sites) may be different. The study size did not allow a more detailed analysis of differential effects of the genotypes by differences in other characteristics, including those adjusted for in the analysis, (e.g., gender, age and baseline body weight).

### Generalisability

The study population originates from various parts of Europe that have different obesity prevalence rates. It can thus be assumed to be rather heterogeneous with respect to the genetic and/or the environmental factors that may influence body weight regulation. Analysis of the allele distribution of the genes tested in the present study has shown that there is great genetic heterogeneity between the populations from the various participating sites (C. Dina et al., unpublished data). We adjusted the analysis (using random effects models) for confounding because of such differences, which, however, assumes that the gene-diet interaction as such is not modified by factors that differ among the various parts of Europe.

The fairly strict inclusion criteria as well as the recruitment basis and procedures may have implications for the generalisability of the results. One obvious such limitation is illustrated by the predominance of women in the trial. On the other hand, this and other aspects of the characteristics of the participants in the trial are likely to represent the patient population seeking dietary treatment for their obesity problem. We have no prior reasons to believe that any of the constraints on the study population would modify the results of the study.

### Overall Evidence

In the NUGENOB trial, we found almost equal mean WL in the two diet groups, but there was a considerable interindividual variation in WL [13], to which genetic variation may contribute. However, the observed effects of the SNPs in the genes were moderate and the findings need to be further investigated before they can be considered contributing to the individual variation in WL during dieting.

Thus, these results do not contribute evidence to future optimisation of dietary treatment of obesity by tailoring the diet to the individual patients according to genotypes that may predict the outcome of the treatment. Although the range of observed WL differences does have clinical significance [59,60], the findings in the present study do not, by themselves, lend support to such use of the genotypes in the panel of genetic polymorphisms tested. When judged to be clinically indicated, either of the dietary regimens investigated may be used for inducing WL in obese individuals in accordance with the previously reported results [13].

Most effects were compatible with dominant and/or additive co-dominant effects, and only SNPs in the *ENPP1* and *WAC* genes had recessive effects, which also were less likely to be discovered because of the lower frequency of homozygotes for the particular variant. The SNPs in the *HSD11B1, TNFα,* and the *WAC* genes and a haplotype of *ENPP1* increased the WL, whereas the SNPs in the *GAD2* and *PCSK1* genes reduced the WL. The SNPs in the *SLC6A14, KCJN11,* and *TNFα* lead to an increased WL by a low-fat diet compared to a high-fat diet, whereas SNPs of the *ENPP1* and *ADIPOQ* had the opposite effect.

The genes in which significant effects of the SNPs were observed belonged to several different categories of presumed function in the obesity-related phenotypes (see Table 2). The *SLC6A14* and *PCSK1* genes and possibly *GAD2* are presumed to influence hypothalamic regulation of appetite [15,17–19]. The *WAC* gene may be involved in regulation of adipocyte differentiation and function [38]. Several of the genes presumed to be associated with regulation of lipid and glucose metabolism (the *HSD11B1, IGF2, KCJN11,* and *ENPP1* genes) [39,42–46] and the production of adipocytokines (the *ADIPOQ* and *TNFα* genes) [47,48,52,53] were involved. Surprisingly, the SNPs in the candidate genes affecting efficiency of energy expenditure (*UCP2* and *UCP3*) showed no effects. Clearly, a better understanding of the specific roles of the SNPs of these genes in altering the function of the multiple, possibly interacting pathways, involved in producing the WL by the particular diets, will require further exploration of how the SNPs affect the intermediate phenotypes, possibly also during overfeeding and weight gain. The differences in effects on WL and the statistical strength of the various SNPs were rather small, so the impact of the SNPs on dietary-induced WL may primarily depend on the frequency of the variant allele, which was highest for the *WAC*, *SLC6A14,* and *KCNJ11* genes, which, for this reason, may deserve particular attention.

The problems in interpretation of the findings in the present study encourage considerations of how the potential of the modern access to the genomic information may be used in the future in such clinical studies, as also exemplified in a recent large-scale study of candidate genes associations to type 2 diabetes [61]. In addition to the obvious request of greater sample sizes, possibly by replication of the study (which may be very challenging and expensive) it should be considered to reduce the statistical noise in the assessments of the correlations and associations between genotypes and phenotypes. A variety of approaches may be employed, including refined definitions of genotypes and phenotypes, better control of background genetic and environmental factors also influencing the phenotype at interest, better study designs and methods for measurements of phenotypes, and further developed statistical tools for handling multiple testing. Identification of intermediate phenotypes closer to the pathway steps to the genes combined with better knowledge about both within and between locus interactions of the genetic variants may both reduce the statistical noise and yield a deeper insight into how the genes operate and how the genetic variation eventually influences the clinically relevant phenotype.

In conclusion, the results of the present study suggest that polymorphisms in several genes associated with obesity-related phenotypes did not have any major impact on weight reduction during short-term either high-fat or low-fat hypo-energetic diet in obese participants, as implemented in the present study. Thus, the results as such do not provide evidence to support revision of current dietary treatment regimens for obesity on the basis of individual genotyping. The results gave tentative leads that some genetic polymorphisms may modulate the diet-induced WL, but this needs to be confirmed and further explored in future studies.

## SUPPORTING INFORMATION

CONSORT Statement Click here for additional data file. (50 KB PDF)

Trial Protocol Click here for additional data file. (567 KB PDF)

Annexes to the Trial Protocol Click here for additional data file. (538 KB PDF)

Alternative Language Abstract S1 Click here for additional data file. Translation of the abstract into Czech.
(28 KB DOC)

Alternative Language Abstract S2 Click here for additional data file. Translation of the abstract into Danish.
(21 KB DOC)

Alternative Language Abstract S3 Click here for additional data file. Translation of the abstract into Dutch.
(21 KB DOC)

Alternative Language Abstract S4 Click here for additional data file. Translation of the abstract into French.
(21 KB DOC)

Alternative Language Abstract S5 Click here for additional data file. Translation of the abstract into Spanish.
(21 KB DOC)

Alternative Language Abstract S6 Click here for additional data file. Translation of the abstract into Swedish.
(21 KB DOC)

## Abbreviations

CI: confidence intervals
NUGENOB: Nutrient-Gene Interactions in Human Obesity: Implications for Dietary Guidelines
WL: weight loss
