# Correction: Genetic Polymorphisms and Weight Loss in Obesity: A Randomised Trial of Hypo-Energetic High- versus Low-Fat Diets

**DOI:** 10.1371/journal.pctr.0010023

**Published:** 2006-08-25

**Authors:** Thorkild I. A Sørensen, Philippe Boutin, Moira A Taylor, Lesli H Larsen, Camilla Verdich, Liselotte Petersen, Claus Holst, Søren M Echwald, Christian Dina, Søren Toubro, Martin Petersen, Jan Polak, Karine Clément, J. Alfredo Martínez, Dominique Langin, Jean-Michel Oppert, Vladimir Stich, Ian Macdonald, Peter Arner, Wim H. M Saris, Oluf Pedersen, Arne Astrup, Philippe Froguel, The NUGENOB Consortium

In *PLoS Clinical Trials*, volume 1, issue 2: DOI: 10.1371/journal.pctr.0010012

The numbering of the reference citations in Table 2 is incorrect. The correct numbering is shown in the table below.

## 

**Table 2:**
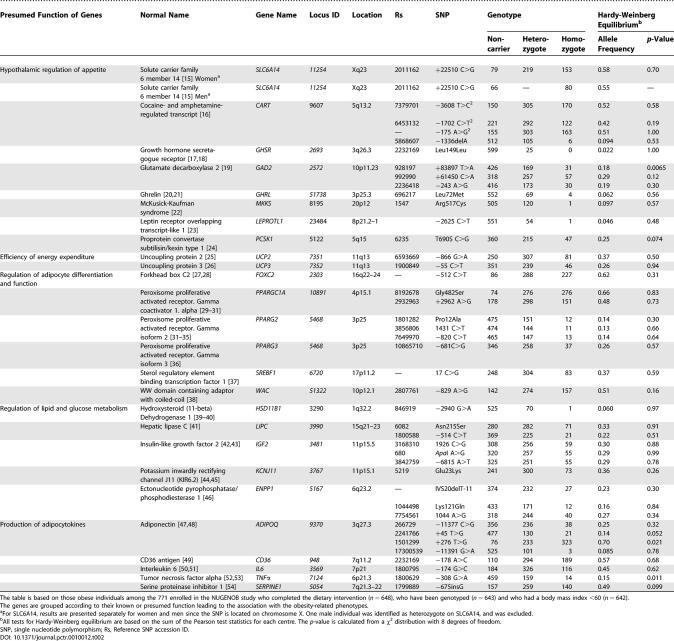
Genotype Distribution and Allele Frequencies in 642 European Obese Women and Men Enrolled in the NUGENOB Study

