# Supplementary material for: Genetic Polymorphisms and Weight Loss in Obesity: A Randomised Trial of Hypo-Energetic High- versus Low-Fat Diets
Source: PLoS Clin Trials. 2006 Jun 30;1(2):e12. doi: 10.1371/journal.pctr.0010012 (PMC1488899; doi:10.1371/journal.pctr.0010012)
Supplement: Trial Protocol [file pctr.0010012.sd002.pdf]

Quality of Life and Management of Living Resources

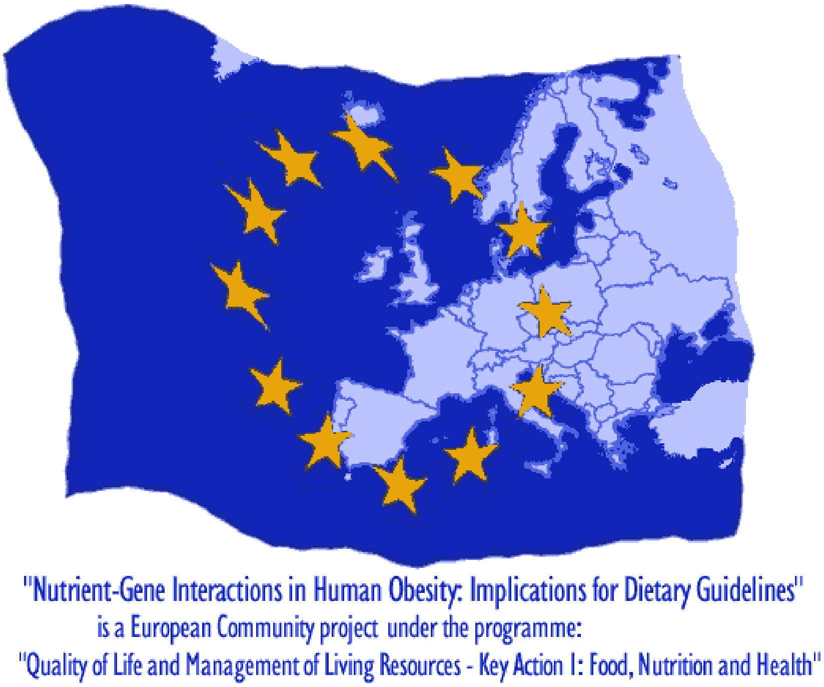

NUTRIENT-GENE INTERACTIONS IN HUMAN OBESITY: IMPLICATIONS FOR DIETARY GUIDELINES

Acronym: NUGENOB

Key Action 1: Food, Nutrition and Health

Original Protocol of 16.01.2001
Notes of 30.03.2006

Table of Contents

1. OBJECTIVES AND EXPECTED ACHIEVEMENTS 3

2. PROJECT WORKPLAN 4

2.1 INTRODUCTION 4

2.2 PROJECT STRUCTURE, PLANNING AND TIME TABLE 6

2.3 DESCRIPTION OF WORKPACKAGES 17

3. ROLE OF PARTIPANTS
(Redundant Chapter - Deleted for convenience reasons)

4. PROJECT MANAGEMENT AND COORDINATION 42

5. EXPLOITATION AND DISSEMINATION ACTIVITIES 45

6. ETHICAL ASPECTS AND SAFETY PROVISIONS 46

30-03-2006

Notes and guidelines for reading this document:

The original Protocol document is dated the 16th of January 2001. The Nugenob project formally started the 1st of March 2001.

All original text (except for Chapter 3 and person-month distributions by partners in the work-packages) is kept in with no marking. In addition to the intervention trial, the study included a number of ancillary studies reported separately. Amendments to the plan for the intervention trial are marked with grey marking.

The relevant SOPs are shown in annex to this document.

1. OBJECTIVES AND EXPECTED ACHIEVEMENTS

Scientific objectives

The overall objective of the project is to elucidate the role in human obesity of interactions between macronutrient composition of the diet with particular emphasis on fat intake and specific genetic variants. It aims at combining clinical/physiological variables to the effects of a very high-fat test meal challenge and a long-term hypoenergetic low-fat or hypoenergetic high-fat diet with knowledge of genetic make up and expression levels of individual genes.

This objective can be divided into the following specific aims:

1. Identification and characterisation of novel nutrient-sensitive candidate genes for obesity, i.e. genes in which variants result in differential responses in obesity-related physiological functions and in adipose tissue to nutrient challenges such as a high-fat meal and a long-term hypoenergetic alteration of dietary fat content.
2. Assessment of the effects of the variants and combinations of variants in known and novel nutrient-sensitive genes on the response in obese subjects to a high-fat test meal in the physiological functions: appetite and satiety, energy expenditure and nutrient partitioning, and circulating obesity-related substrates, hormones and metabolites.
3. Assessment of the combined effects the variants of novel and known nutrient-sensitive genes and a short- and long-term alterations in dietary fat content on the differential expression of selected functional genes in adipose tissue.
4. Identification of predictors of the changes in body weight and composition of obese subjects during a long-term hypoenergetic low-fat or high-fat dietary intervention programme. These predictors may be: a) variants or combinations of variants of the nutrient-sensitive genes, b) the obesity-related life style factors, c) the differential physiological functions observed at the test meal challenge, d) the gene expression in adipose tissue, or e) gene-phenotype or gene-environment interactions based on combinations of these predictors.

Expected achievements

By fulfilling these objectives, achievements of a great and useful increase in our understanding of the interaction between dietary fat and the genetic predisposition of obesity are expected. This new knowledge will improve in several ways the basis for the ability to limit the development of the epidemic of obesity by more effective and more precisely targeted prevention and treatment.

More specifically, the expectation is to achieve more precise knowledge about, and improved understanding of:

1. Genomic position and structure, functional variants, and regulation of several novel nutrient-sensitive genes that may be involved in the pathogenesis of obesity.

2. The specific mechanisms underlying the well documented genetic predisposition to obesity, which is polygenic and probably heterogeneous with different genes playing a major role in different subsets of obese subjects.

3. Genes actively involved in the regulation of metabolic efficiency, in excessive accumulation of fat in adipose tissue and in the changes in fat content of the adipose tissue induced by alterations of the dietary fat content.

4. The complex role of fat intake in the pathogenesis of obesity by disclosing the specific nutrient-gene interactions both at a challenge of a single high-fat meal and during a long-term low-fat or high-fat dietary intervention.

5. The inter-individual variation in the response to a fat challenge by evaluating the physiological responses to a high-fat test meal in relation to the specific genotypes of the

obese subjects, which will characterise the obese subjects with regard to their ability to metabolise fat.

6. The inter-individual variation in the changes in body weight and composition during a long-term hypoenergetic low-fat or high-fat dietary intervention.

It is envisaged that the results of this project may lay the grounds for:

7. Development of a new obesity taxonomy, in which new modes of classification of subtypes of obesity are based upon their specific genotypes, and the nutrient-gene interaction emerging during the high-fat test meal or the hypoenergetic low-fat or high-fat dietary intervention programme.
8. Development of diagnostic tools - on the basis of the genotyping and the responses to the challenge to a high-fat meal - that can discriminate obese subjects with respect to effectiveness of a long-term hypoenergetic low-fat or high-fat dietary intervention allowing accurate targeting of this intervention and delineation of obese subjects in whom other means of intervention are needed.

2. PROJECT WORKPLAN

2.1 Introduction

The basic logic of the project design is the comparison of subsets of obese subjects with different specific genotypes with regard to the observed specific phenotypes either naturally occurring or experimentally induced. The possible deviance of the observed phenotypes of the genotype specific subsets of obese is assessed by comparison to a group of reference subjects, representing the underlying population with the same genotype. The investigations will be conducted at eight different sites in Europe where adequate source populations of obese and reference subjects of Caucasian origin are available in combination with the required expertise to conduct the planned investigation programme. Compared to fewer sites in limited parts of Europe, this sampling design will increase the variation in genotypes, in environmental exposures and gene-environmental interactions, and thereby the likelihood of sampling sufficiently sized genotype-specific subsets. The source populations at these sites are described in WP1. The plan is to recruit from these source populations according to specified criteria, a total of 750 obese subjects and 115 reference subjects constituting the study population during a 12 month enrolment period as described in WP2.

When the subjects have been recruited for the study, they will first be invited to an assessment of their food habits by a food frequency questionnaire and a prospective 3-day weighed food recording (WP3). On the same occasion, their physical activity habits, smoking and alcohol drinking habits, drug consumption, clinically managed diseases, and psychosocial profile will be assessed by interviews (WP3). They will be instructed by a dietician about standardisation of their diet to a medium fat content (37% of total energy, though with the local, individual food composition maintained) during a 3-day run-in period prior to the test meal day, whereby the effects of the inter-individual variation in habitual diet on the results of the physiological investigations carried out along with the test meal are minimised (WP3).

On the test meal day (WP4), the subjects will first undergo anthropometry and body composition assessment, blood will be drawn and prepared for the DNA analysis (WP6), and biopsies will be

taken from the abdominal subcutaneous fat tissue. The subjects will be given a high-fat liquid test meal (60% of energy as fat) in order to challenge the physiological reactions to such fat load (WP4). Before, and repeatedly during the three hours after ingestion of the meal, we will assess appetite (hunger/satiety scoring), energy expenditure and respiratory quotient (indirect calorimetry by ventilated hoods), and circulating obesity-related hormones and metabolites (WP4). Each blood analysis will be carried out in one laboratory to secure standardization of the analyses. After the test meal, another biopsy will be taken from the abdominal subcutaneous fat tissue.

Following the test meal and clinical examination day, the obese subjects will be randomised and enrolled into a randomised two-arm open label 10-weeks dietary intervention consisting of a diet either low or high in fat (20-25% or 40-45% energy as fat) and with a 600 kcal/d less energy content than estimated as the daily requirements for the individual (WP5). After the 10 weeks, the baseline assessment of the dietary record, anthropometry, body composition, and abdominal subcutaneous fat biopsies will be repeated (WP4).

With the purpose of screening for variants of the relevant candidate genes of the subjects of the study population, blood will be sampled for DNA extraction and establishment of a DNA bank by Partner 3 (WP6).

There will be three sources of putatively relevant nutrient-sensitive candidate genes. Using different settings and study population, Partner 4/13 is conducting linkage disequilibrium mapping and fine mapping to identify genomic loci containing putative candidate genes (WP7). Both Partners 3 and 4 contribute with selection of positional candidate genes inside or outside these loci on the basis of bioinformatics and homology to other known genes, and among these genes functional candidate genes will be selected based upon the presumed function of the gene products in relation to nutrient metabolism (WP7). Partners 6a and 11 will use the adipose tissue mRNA before and after the dietary challenges to identify putatively relevant candidate genes (WP11).

Partners 3 and 4 will conduct analysis of the candidate genes by cloning and sequencing, and scanning for variants, i.e. single nucleotide polymorphisms (SNPs) or insertions and deletions, by PCR based detection of single strand conformational polymorphism and by a new dHPLC system and capillary sequencing equipment (WP8).

When relevant candidate genes have been selected and investigated for variants, Partners 3 and 4 will carry out the genotypic screening of the study population after PCR amplification. Currently available techniques, such as restriction length fragment polymorphism (RFLP) analysis, micro-sequencing, allele-specific oligonucleotide hybridisation (ASO), or restriction-site generating PCR (RG-PCR) in combination with RFLP analysis will be used, together with the novel Invader™ technology allowing genotyping without the need for prior PCR-amplification (WP9).

The adipose tissue biopsies taken before and 3 hours after the fat load (WP4) and again after the dietary interventions (WP5) will be shipped to Partner 11 by the Partners conducting the intervention programmes. The mRNA will be prepared from these tissue biopsies and will be utilised for two purposes, quantitative assessment of expression of selected genes (WP10), and identification of putatively relevant nutrient-sensitive candidate genes (WP11).

The quantitative assessment of gene expression, i.e. the steady state mRNA levels, in adipose tissue will be assessed by already established technology by Partners 6a and 11 together with a newly established commercial laboratory under a subcontract, who will use solution

hybridisation for highly expressed genes and the quantitative reverse transcriptase PCR (RT-PCR) technique for low-level expressed genes (WP10).

Analysis of variations in mRNA expression will be performed using a combination of cDNA arrays and RDA (representational difference analysis) technologies (WP11), allowing for the identification of previously unknown genes. Information on the novel DNA sequences produced may also be used to motivate the scanning for gene variants and genotyping of the entire study population (WP8-9), either using biopsy-derived cDNA or genomic DNA available on all subjects (WP8-9).

All the data generated on the subjects enrolled into the study population will be forwarded to Partner 1 and integrated into a databank from which data will extracted for statistical analysis addressing the questions derived from the specific aims (WP12). The plans for statistical analysis will be thoroughly discussed and decided on by the Steering Committee and thereafter conducted by a professional statistician applying the techniques described in WP12. The results of the statistical analysis will be submitted in written reports to the members of the consortium.

2.2 Project structure, planning and timetable

List of Partners

Partner 1 (IPM): Professor, Dr.Med.Sci. Thorkild I.A. Soerensen
Institute of Preventive Medicine, Copenhagen Hospital Corporation
H:S-Kommunehospitalet, opg 23a
DK-1399 Copenhagen K, Denmark
Tel.: (45) 33383860, Fax: (45) 33324240, E-mail: tias@ipm.hosp.dk

Partner 2 (KVL.RDHN): Professor, Dr.Med.Sci. Arne Astrup
The Royal Veterinary and Agricultural University
Research Dept. of Human Nutrition
Rolighedsvej 30
DK-8 Frederiksberg C
Tel: (45) 35282476, Fax. (45) 35282483, E-mail ast@kvl.dk

Partner 3 (NN.SDC): Professor, Dr.Med.Sci. Oluf Borbye Pedersen
Steno Diabetes Center, Novo Nordisk A/S Denmark
Niels Steensens Vej 2,
DK-2820 Gentofte, Denmark
Tel. (45) 44439050, Fax: (45) 44438232, E-mail: Oluf@steno.dk

Partner 4/13:
Partner 4 (CNRS.LGMM) and Partner 13 (UL2.GMMF): Professor Philippe Froguel, MD, Ph D
Head of the Department of Human Genetics, Institute of Biology,
CNRS 8090, Institut Pasteur de Lille,
1 rue Calmette, 59000 Lille, France
Tel.: (33) 320877954 and 79 11, Fax: (33) 320877229,
E-mail: Philippe.Froguel@mail-good.pasteur-lille.fr

Partner 5 (RULIMB.NTRIM): Professor Wim H.M. Saris, MD, PhD
Dept. of Human Biology, Nutrition Research Centre NUTRIM
Maastricht University
PO Box 616, NL-6200 MD Maastricht
The Netherlands
Tel.: (31) 43 881619, Fax: (31) 433670976, E-mail: W.Saris@HB.Unimaas.nl

Partner 6a (KI.DM): Professor, Dr.Med. Peter Arner
The Lipid Laboratory at the Dept. of Medicine
Karolinska Institute, Huddinge University Hospital
S-141 86 Huddinge, Sweden
Tel.: (46) 858582342, Fax: (46) 8 58587223, E-mail: Peter.Arner@medhs.ki.se
Partner 6b (KI.DM): Professor, Dr.Med. Stephan Roessner (Partner 6a is Contract holder)
The Obesity Unit at the Dept. of Medicine
Karolinska Institute, Huddinge University Hospital
SE-141 86 HUDDINGE, Sweden
Tel.: (46) 8 58586997, Fax: (46) 87749962, E-mail: Stephan.Rossner@medhs.ki.se

Partner 8 (UNOTT.SBSS): Professor Ian A. Macdonald,
School of Biomedical Sciences,
University of Nottingham Medical School, Queen's Medical Centre, Clifton Boulevard
Nottingham, NG7 2UH, UK
Tel.: (44) 115 9709465, Fax: (44) 115 9709259, E-mail: ian.macdonald@nottingham.ac.uk

Partner 9 (UCPRA.SP): Vladimir Stich, MD
Dept. of Sports Medicine, Center of Preventive Medicine,
Third Faculty of Medicine, Charles University
Ruská 87, 100 00 Praha 10, Czech Republic
Tel.: (420) 2 67102209 or 67102483, Fax: (420) 2 67102263 (8-14), 14 - (420) 2 72730776
E-mail: vladimir.stich@lf3.cuni.cz

Partner 10 (UPMC.DM): Professeur Bernard Guy-Grand
Dept. of Nutrition, Hôtel-Dieu
1, Place du Parvis Notre-Dame
75181 Paris 04, France
Tel.: (33) 1 42348451, Fax: (33) 1 405 0057, E-mail: bernard.guy-grand@htd.ap-hop-paris.fr

Partner 11 (INSERM.ILB): Dominique Langin, D.V.M., Ph.D.
Directeur de recherches, INSERM U317
Institut Louis Bugnard
Bat. L3, CHU Rangueil
F-31403 Toulouse Cedex 4
Tel.: (33) 5 62172950, Fax: (33) 5 61331721, E-mail: Dominique.Langin@toulouse.inserm.fr

Partner 12 (UNAV.DFN): Professor J. Alfredo Martínez
Dpto. Fisiología y Nutrición, Universidad de Navarra
C/. Irunlarrea s/n.
31008 - Pamplona (Navarra), Spain
Tel.: (34) 948 425600, Fax: (34) 948 425649, E-mail: jalfmtz@unav.es

Subcontractor: Dr. Hubert VIDAL, Directeur de Recherche INSERM, EZUS-LYON1, Lyon, France Hubert.Vidal@laennec.univ-lyon1.fr (Subcontractor to Partner 1)

Workpackage list

Workpackage N°	Workpackage Title	Responsible partners n°[1]	Person-months	Start month	End month	Deliverable n°
1	Source populations	1	1.0	1	14	D1.1.-4.
2	Recruitment of study population	1	1.0	2	14	D2.1.-4.
3	Habitual and run-in diet, life style factors	5	46.3	3	15	D3.1.-3
4	Clinical Investigation Day	5	152.6	3	16	D4.1.-6.
5	Dietary intervention programme	2	112.4	3	19	D5.1.-4.
6	Sampling and processing of DNA	3	7.0	3	15	D6.1.-2.
7	Identification of candidate genes	4/13	14.0	1	24	D7.1.-2.
8	Analysis of candidate genes	3	73.0	1	24	D8.1.-3.
9	Genotypic screening	3	43.9	15	30	D9.1.-5.
10	Adipose tissue mRNA analysis	11	31.0	8	30	D10.1.-4.
11	Gene-expression profiling in adipose tissue	6	69.0	1	32	D11.1.-5.
12	Databank and statistical analysis	1	18.0	1	36	D12.1.-17.
		**TOTAL**	569.2			

[1] Workpackage leader to be listed first

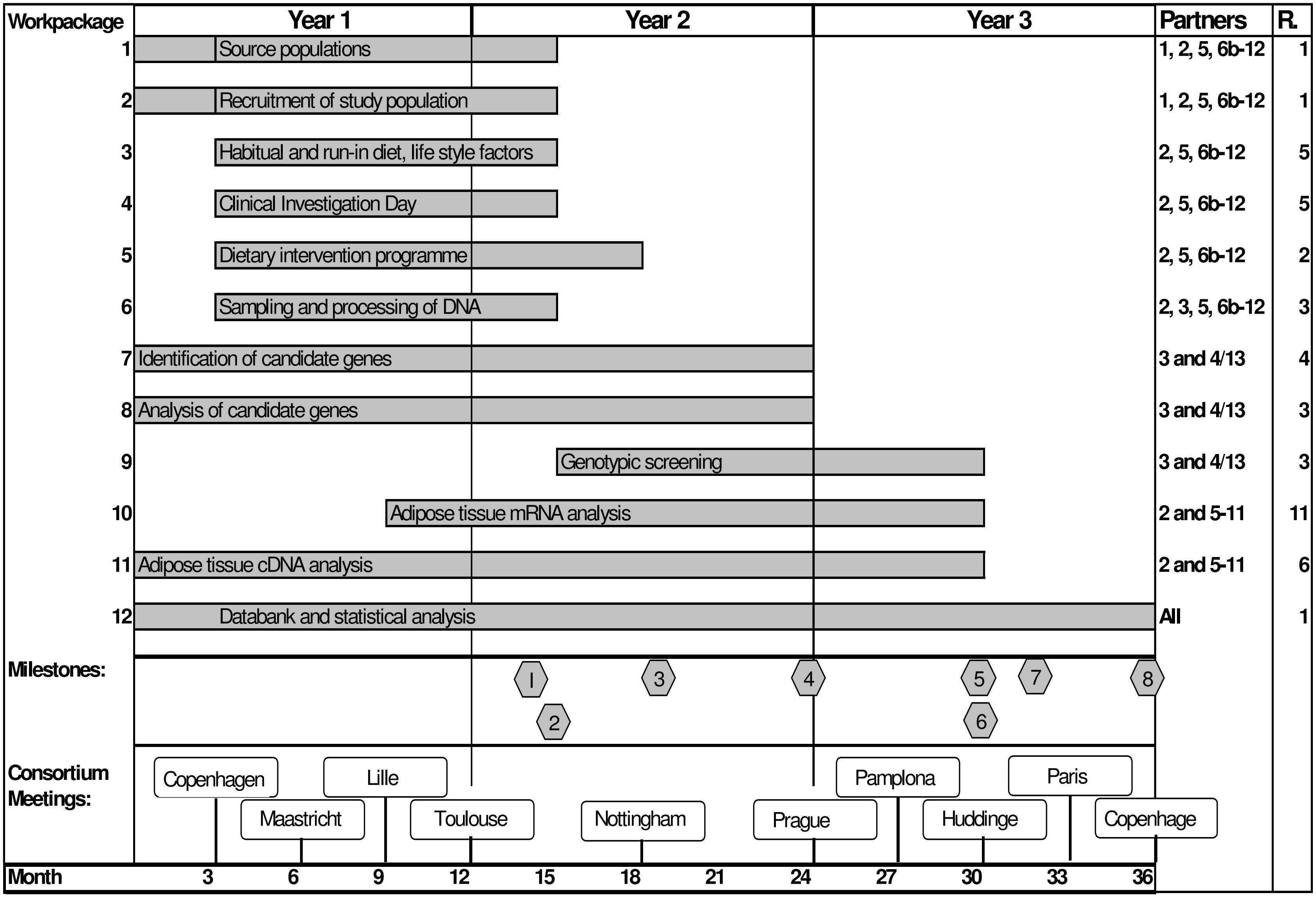

Workpackage
Year 1
Year 2
Year 3
Partners
R.
1
Source populations
1, 2, 5, 6b-12
1
2
Recruitment of study population
1, 2, 5, 6b-12
1
3
Habitual and run-in diet, life style factors
2, 5, 6b-12
5
4
Clinical Investigation Day
2, 5, 6b-12
5
5
Dietary intervention programme
2, 5, 6b-12
2
6
Sampling and processing of DNA
2, 3, 5, 6b-12
3
7
Identification of candidate genes
3 and 4/13
4
8
Analysis of candidate genes
3 and 4/13
3
9
Genotypic screening
3 and 4/13
3
10
Adipose tissue mRNA analysis
2 and 5-11
11
11
Adipose tissue cDNA analysis
2 and 5-11
6
12
Databank and statistical analysis
All
1
Milestones:
1
2
3
4
5
6
7
8
Consortium Meetings:
Copenhagen
Lille
Maastricht
Toulouse
Nottingham
Prague
Pamplona
Huddinge
Paris
Copenhage
Month
3
6
9
12
15
18
21
24
27
30
33
36

List of Milestones

Milestone N°	Title	Delivery date Months	Partners	Description
1	Identifying and recruiting the study population	14	1, 2, 5, 6b-12	Clinical epidemiological work identifying and recruiting the study population of 750 obese and 115 reference subjects (WP1-2)
2	High-fat test meal and clinical investigation	16	1, 2, 5, 6b-12	Single high-fat test meal challenge and synchronous clinical investigation following baseline assessment and a standardised diet run-in period (WP 3-4)
3	Dietary intervention programme	19	1, 2, 5, 6b-12	A 10-week hypoenergitic either low-fat or high-fat dietary intervention programme for the obese study population (WP 5)
4	Nutrient-sensitive candidate genes	24	1, 3, 4/13	Molecular genetics studies on identification, analysis, and screening of putatively relevant, known or novel, nutrient-sensitive candidate genes (WP 6-8)
5	Genotypic screeing	30	1, 3, 4/13	Genotypic screening of the entire study population for selected functional variants of putatively nutrition-sensitive genes (WP 9)
6	Adipose tissue mRNA assays	30	1, 6, 11	Adipose tissue sampling and quantitative assessment of gene expression by mRNA assays (WP 10)
7	Adipose tissue gene expression profiling	32	1, 6, 11	Adipose tissue gene expression profiling by cDNA arrays for nutrient-sensitive genes (WP 11)
8	Database and conduct of statistical analyses	36	1-13	Collection and integration of the generated data in a database and conduct of statistical analyses of these data (WP 12)

List of Deliverables

Deliverable N°	Title	Delivery date Month	Nature	Dissemination level[1]	Dissemination target²
D1.1.	Source population list, structure.	3	R	CO	WP2
D1.2.	Local source population list for local recruitment, continuously built up, at least 50% expected to be identified.	9	R	CO, RE	WP2
D1.3.	Local source population list for local recruitment, continuously built op, 100% expected to be identified	14	R	CO, RE	WP2
D1.4.	Source population list, completed.	14	R	CO, RE	WP12
D2.1.	Study population list, structure.	3	R	CO	WP3
D2.2.	Local source population list for local recruitment, continuously built up, at least 50% expected to be identified.	9	R	CO, RE	WP3
D2.3.	Local source population list for local recruitment, continuously built op, 100% expected to be identified.	14	R	CO, RE	WP3
D2.4.	Study population list, completed.	14	R	CO, RE	WP12
D3.1.	Study subjects prepared for the Clinical Invistigation Day.	3-15	Subjects	CO, RE	WP4
D3.2.	Results of each subject investigation continuously transferred to the databank, 40% expected to be delivered.	10	R	CO, RE	WP12
D3.3.	Results of each subject investigation continuously transferred to the databank, 100% expected to be delivered.	15	R	CO, RE	WP12
D4.1.	Obese study subjects to be transferred to theDietary intervention programme.	3-15	Subjects	CO, RE	WP5
D4.2.	Blood samples delivered to biochemical analysis and a biobank in Maastrict	3-15	Subjects	CO, RE	Laboratory of Partner 5 and sub-contractor for WP4 analysis
D4.3.	Blood samples delivered to sampling and processing of DNA and to a biobank in Copenhagen	3-15	Subjects	CO, RE	Partner 5 for WP6
D4.4.	Adipose tissue biopsy samples delivered to mRNA analysis and to a biobank in Toulouse	3-15	Subjects	CO, RE	Partner 11 for WP10, WP11, WP12
D4.5.	Results of each subject investigation continuously transferred to the databank, 40% expected to be delivered.	10	R	CO, RE	WP12
D4.6.	Results of each subject investigation continuously transferred to the databank, 100% expected to be delivered.	15	R	CO, RE	WP12

Deliverable N°	Title	Delivery date Month	Nature	Dissemination level[1]	Dissemination target[2]
D5.1.	Results of each subject investigation continuously transferred to the databank, 40% expected to be delivered.	13	R	CO, RE	WP12
D5.2.	Results of each subject investigation continuously transferred to the databank, 100% expected to be delivered	19	R	CO, RE	WP12
D5.3.	Blood samples delivered to biochemical analysis and a biobank in Maastrict	6-18	Samples	CO, RE	Laboratory of Partner 5 and sub-contractor for WP4 analysis.
D5.4.	Adipose tissue biopsy samples delivered to mRNA analysis and to a biobank in Toulouse	6-18	Samples	CO, RE	Partner 11 for WP10, WP11, WP12
D6.1.	DNA samples in the DNA bank from the study population to be collected and processed continuously during month 3-15, 40% expected to be achieved.	9	R	CO, RE	WP7, WP8, WP9, WP12
D6.2.	DNA samples in the DNA bank from the study population to be collected and processed continuously during month 3-15, 100% expected to be achieved.	15	R	CO, RE	WP7, WP8, WP9, WP12
D7.1.	List of identified candidate genes based on: mapping linkage disequilibrium regions on chromosomes 10, 2, 5, and 20; definition of 1-5 cM regions where the putative obesity genes map; physical mapping in the 1-2 cM linked region.	12	R	CO, RE	WP8, WP9, WP10<br>Scientific reports
D7.2.	List based on: screening of all gene banks for genes in the linkage disequilibrium region to find and test novel candidate genes; and clone full-length cDNA and gene organisation of novel genes.	24	R	CO, RE	WP8, WP9, WP10<br>Scientific reports
D8.1.	List of gene variants corresponding to markers (SNPs and others) of relevant candidate genes.	12	R	CO, RE	WP9<br>Scientific reports
D8.2.	List of gene variants corresponding to markers (SNPs and others) of relevant candidate genes.	18	R	CO, RE	WP9<br>Scientific reports
D8.3.	List of gene variants corresponding to markers (SNPs and others) of relevant candidate genes.	24	R	CO, RE	WP9<br>Scientific reports
D9.1.	Genotypes of the entire study population of variants in the selected candidate genes.	18	R	CO, RE	WP10, WP12
D9.2.	Genotypes of the entire study population of variants in the selected candidate genes.	21	R	CO, RE	WP10, WP12
D9.3.	Genotypes of the entire study population of variants in the selected candidate genes	24	R	CO, RE	WP10, WP12

Deliverable N°	Title	Delivery date Month	Nature	Dissemination level[1]	Dissemination target²
D9.4.	Genotypes of the entire study population of variants in the selected candidate genes	27	R	CO, RE	WP10, WP12
D9.5.	Genotypes of the entire study population of variants in the selected candidate genes	30	R	CO, RE	WP10, WP12
D10.1	Quantitative gene expression data on 10 selected known and novel candidate genes.	12	R	CO, RE	WP12
D10.2.	Quantitative gene expression data on 10 selected known and novel candidate genes.	16	R	CO, RE	WP12
D10.3.	Quantitative gene expression data on 10 selected known and novel candidate genes.	24	R	CO, RE	WP12
D10.4.	Quantitative gene expression data on 10 selected known and novel candidate genes.	30	R	CO, RE	WP12
D11.1.	Development of the RDA and the DNA chips technology.	7	R	CO, RE	Scientific reports
D11.2.	Completion of development of the technology	12	R	CO, RE	Scientific reports
D11.3.	List of genes of which gene expression depends on the nutritional experimental manipulations.	18	R	CO, RE	WP7, WP8, WP10, WP12
D11.4	List of genes of which gene expression depends on the nutritional experimental manipulations.	24	R	CO, RE	WP10, WP12
D11.5.	List of genes of which gene expression depends on the nutritional experimental manipulations.	32	R	CO, RE	WP12
D12.1.	Descriptive statistics on the accumulated information in the databank.	3	R	CO	Consortium feedback
D12.2.	Descriptive statistics on the accumulated information in the databank.	6	R	CO	Consortium feedback
D12.3.	Descriptive statistics on the accumulated information in the databank.	9	R	CO	Consortium feedback
D12.4.	Descriptive statistics on the accumulated information in the databank.	12	R	CO	Consortium feedback
D12.5.	Descriptive statistics on the accumulated information in the databank.	15	R	CO	Consortium feedback
D12.6.	Descriptive statistics on the accumulated information in the databank.	18	R	CO	Consortium feedback
D12.7.	Descriptive statistics on the accumulated information in the databank.	21	R	CO	Consortium feedback

Deliverable N°	Title	Delivery date Month	Nature	Dissemination level[1]	Dissemination target[2]
D12.8.	Descriptive statistics on the accumulated information in the databank.	24	R	CO	Consortium feedback
D12.9.	Descriptive statistics on the accumulated information in the databank.	27	R	CO	Consortium feedback
D12.10.	Descriptive statistics on the accumulated information in the databank.	30	R	CO	Consortium feedback
D12.11.	Descriptive statistics on the accumulated information in the databank.	33	R	CO	Consortium feedback
D12.12.	Results of statistical analysis of the accumulated data.	21	R	CO, RE	Assigned authors of scientific reports
D12.13.	Results of statistical analysis of the accumulated data.	24	R	CO, RE	Assigned authors of scientific reports
D12.14.	Results of statistical analysis of the accumulated data.	27	R	CO, RE	Assigned authors of scientific reports
D12.15.	Results of statistical analysis of the accumulated data.	30	R	CO, RE	Assigned authors of scientific reports
D12.16.	Results of statistical analysis of the accumulated data.	33	R	CO, RE	Assigned authors of scientific reports
D12.17.	Results of statistical analysis of the accumulated data.	36	R	CO, RE	Assigned authors of scientific reports

[1]PU=Public, RE =restricted to a group specified by the consortium (including EC services), CO = confidential, only for members of the consortium (including EC services)
[2]Indicate the target audience or the potential users/beneficiaries of such a deliverable

PERT DIAGRAM

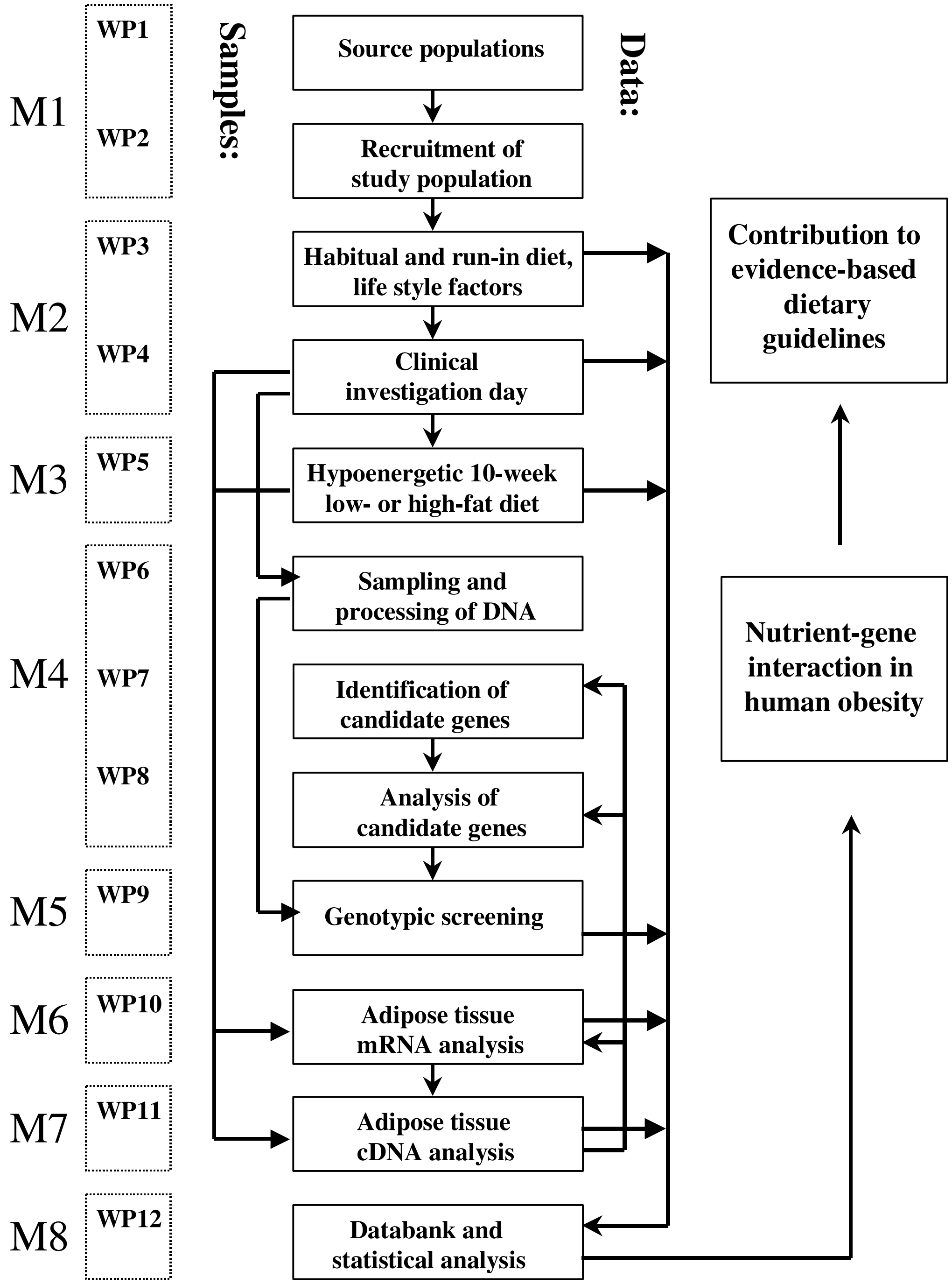

Pert Diagram:

The M numbers refer to the Milestones and the WP numbers to the Workpage 2 numbers.

Work package protocol summary of the conduction of the study

Workpackage number: 1: Source populations

Objectives
To identify at the participating partners 2, 5, and 6b-12 suitable source populations of obese subjects and background populations from which the subjects may be selected for the study population of obese and reference subjects.

Methodology and study material
The eight participating centres contributing obese subjects, will identify, explicitly define and describe their source populations of obese subjects with particular emphasis on how the subjects become members of the source populations and what kind of pertinent information about their obesity and related aspects is already available. For most of the partners, the source populations will consist of obese patients referred to their units for treatment of obesity. The referral procedures will to the extent possible be described in such a way that reference subjects may be randomly sampled from the same background population. Some of the centres (partners 2, 5 and 10) will in addition have access to ongoing or recently carried out population-based surveys through which both the obese and the reference subjects may be drawn. These population surveys will be similarly described. The quantitative dynamics of the source populations will be given particular attention due to the implications for current recruitment of subjects throughout the study period of 1 year. In each centre, there will be set up similarly formatted rosters of identified members of the source populations from which the selection for the study population can take place (WP2). These rosters will contain the relevant information for the selection procedure, i.e. ethic origin, gender, age, height, weight (combined to body mass index, weight/height^2), known clinically diagnosed and treated diseases. Blood samples for isolating genomic DNA - drawn in the setting of other studies conducted on these source populations - will be made available for initial genetic studies (WP7-8).

Deliverables
- D1.1. Source population list, structure, delivered to WP2 at Month 3
- D1.2. Local source population list for local recruitment, continuously built up, at least 50% expected to be identified by Month 9, Confidential list supplying WP2.
- D1.3. Local source population list for local recruitment, continuously built up, 100% expected to be identified by Month 14, confidential list supplying WP2.
- D1.4. Source population list, completed, Month 14. Confidential list contributing to WP12 and as anonymous statistics delivered to assigned authors of scientific reports.

Milestones
We expect that this workpackage results in eight well-characterised source populations suitable for the recruitment of the study population. Thereby, the workpackage contributes (together with WP2) to *Milestone 1*: Clinical epidemiological work identifying and recruiting the study population of 750 obese and 115 reference subjects.

Modifications of the original WP plan

From the beginning it became clear that many subjects referred for treatment of obesity had concomitant health problems, and were therefore not eligible for inclusion. It was therefore not feasible to define and describe their source populations of obese subjects with particular

emphasis on how the subjects become members of the source populations and what kind of pertinent information about their obesity and related aspects is already available.

Instead subjects were recruited from different sources, including advertising in the local press, existing waiting lists for treatment of obesity and ongoing population studies, self-referral or referral from general physicians or other clinical units, local obesity organisations, and through personal communication. Lean subjects were recruited through personal communication (among colleagues, families and friends), and through advertising in the local press. The lean subjects were recruited in order to match the age and sex distribution of the obese subjects. This matching for age and sex was conducted centre-wise.

SOPs developed for WP1

None

Workpackage number: 2: Recruitment of study population

Objectives
To recruit the subjects from the source populations (WP1) for the study population to be enrolled into the clinical investigation and intervention programme (WP3-5). The study population will include 750 obese subjects and, as a reference group, 115 subjects representative of the general population from which the obese has been selected. The obese study population will be recruited independent of their genotypes, but only among subjects of Caucasian origin.

Methodology and study material
As described in WP1, there will be established rosters of the subjects of the source populations of obese subjects and of randomly selected subjects from the general populations within the same gender and age strata at the eight partners 2, 5, and 6b-12. On the basis of these rosters, the local project secretary will identify on a weekly basis the candidates to be selected for the study population to be enrolled in the investigation and intervention programme. Obesity will be defined as body mass index exceeding 30 kg/m^2. Caucasians (by selfreport) of both genders between the ages 20-50 years will be enrolled. Every week, 2-3 obese subjects will be selected, invited, and appointed for the programme. This will be running for about 40 weeks in eight centres, of which seven (Partners 2, 5, 6b-10, 12) recruit 100 and Partner 11 recruits 50 obese subjects, thus providing in total 750 obese subjects. In each of the seven centers 15 and in Partner 11 centre 10 reference subject will be enrolled for the clinical investigation programme (WP3-4) and hence 115 reference subjects will be recruited for the study population. Continued selection and invitation from the rosters of the source populations will cope with rejection of participation so that the planned number of subjects appointed per week will be achieved. The basic selection criteria for both the obese and the reference subjects will be that they provide informed consent, are collaborative, non-pregnant, not abusing alcohol or drugs, and are feeling so healthy that they are capable of working full time. The women should be pre-menopausal, and any use of oral contraceptives will be recorded and taken into account in the analysis. Specifically, the subjects may not suffer from clinically diagnosed and treated diabetes, hypertension, hyperlipidaemia, thyroid diseases, or other diseases, which by themselves or via their treatments are suspected of influencing the results of the investigations. Both the obese and reference subjects will be selected without regard to the their specific genotypes.

Deliverables
- D2.1. Study population list, structure, delivered to WP3 at Month 3.
- D2.2. Local study population list indicating local recruitment, continuously built up, at least 50% expected to be identified by Month 9, Confidential list supplying WP3.
- D2.3. Local study population list indicating local recruitment, continuously built up, 100% expected to be identified by Month 14, Confidential list supplying WP3.
- D2.4. Study population list, completed, Month 14. Confidential list contributing to WP12 and as anonymous statistics delivered to assigned authors of scientific reports.

Milestones
This workpackage is expected to result in a well defined and well characterised series of 750 obese and 115 reference subjects representing the background population, whereby the workpackage contributes, together with WP1, to completion of *Milestone 1*: Clinical epidemiological work identifying and recruiting the study population of 750 obese and 115 reference subjects.

Modifications of the original WP plan
None

SOPs developed for WP2

SOP for Inclusion and Exclusion of Subjects ***(Included in annex)***

SOP for anthropometry ***(Included in annex)***

SOP for bioimpedance

Workpackage number: 3: Habitual and run-in diet, life style factors

Objectives
To assess daily food intake, physical activity habits, and other obesity-related life style factors of the subjects at baseline of the study. To standardize food intake during a 3-day dietary run-in period prior to the Clinical Investigation Day (WP4).

Methodology and study material
The subjects recruited (WP2) will be contacted by a dietician 2-3 weeks before the Clinical Investigation Day (WP4) and informed that the following questionnaires will be mailed to their address within a few days: a) food intake (Ocke et al, Int J Epidemiol 1997;26(Suppl 1):S37-48; b) physical activity (Baecke et al, Am J Clin Nutr 1982;36(5):932-42; c) life style (Ware JE et al, Medical care 1992;30(6):473-83); d) full medical history including daily medication, smoking and alcohol habits. Also during this contact an appointment is settled for a visit to the research centre approximately 10 days before the Clinical Investigation Day. At this visit the questionnaires are checked by a dietician and the subject instructed about: a) how to perform a 3-day weighed food record (two weekdays and one weekend day) - for which a calibrated standard food scale for weighing their foods will be handed out; b) a standard diet for the final 3-day dietary run-in period prior to the Clinical Investigation Day (WP4). The standard diet is based on information from their individual food habits and should maintain body weight (Energy and protein requirement. WHO Technical report series 724. Geneva 1985). The diet should provide a macronutrient composition close to 15 E% (% of energy) protein, 35 E% fat, and 50 E% carbohydrate.

Deliverables
- D3.1. Study subjects prepared for WP4 , the Clinical Investigation Day.
- D3.2. Report at Month 10 on results of each subject investigation, continuously transferred to the databank, 40% of the reports expected to be delivered to WP12.
- D3.2. Report at Month 15 on results of each subject investigation, continuously transferred to the databank, 100% of the reports expected to be delivered to WP12.

Milestones
This workpackage is expected to result in a thorough characterisation of habitual food intake, physical activity habits, and other obesity-related life style factors, and psychosocial condition for the obese and reference study population. In addition, an adequate standardisation of the diet through a 3-day run-in period is expected to prepare the subjects for the Clinical Investigation Day. Thereby, this workpackage contributes, together with WP4, to completion of *Milestone 2:* Single high-fat test meal challenge and synchronous clinical investigation following baseline assessments and a standardised diet run-in period.

Modifications of the original WP plan

It was decided to leave out the 3-day dietary standardisation period prior to the clinical investigation day. The subjects were therefore instructed to eat as they do normally.

SOPs developed for WP3

The Nugenob Questionnaire

SOP for food recording is included as part of the SOP for the Dietary Intervention ***(included in WP5)***

Workpackage number: 4: Clinical Investigation Day

Objectives

To develop and execute a standardised clinical investigation protocol characterising obese and reference subjects phenotypically during a one-day visit to the clinic. The protocol includes measurements characterising in detail the obese vs lean subjects with respect to body composition and fat distribution, biochemical indices (circulating hormones, substrates, and metabolites). Obesity is characterised by various degrees of impaired fat oxidation capacity. Therefore, a functional fat challenge test meal has been developed in order to characterise the subject's ability to metabolise an acute high fat load. This includes measurement of the thermogenic and oxidation pattern, the biochemical profiles in blood, together with hunger/satiety score. In addition, a fat biopsy will be taken from the subcutaneous periumbilical adipose tissue preprandial and 3 hours postprandial for mRNA studies (WP10 and WP11)

Methodology and study material

The subjects arrive at the research centre at 7.30 a.m. after a 12 hours overnight fast and a preceding 3-day dietary run-in period (WP3). After the subjects have voided the bladder, weight, height, and waist/hip ratio will be determined in underclothes. Body composition will be determined by bio-impedance with the subjects in supine position. A catheter is inserted into an antecubital forearm vein for blood sampling. The room is kept thermoneutral at 25°C and the subjects stay in bed for the next 3.5 hours. They are allowed to watch light movies or listen to the radio, to keep them relaxed. To study the thermogenic capacity and nutrient partitioning, the response to a saturated fat load is measured. This liquid meal consists of 60 E% (% of energy) saturated fat, P/S ratio 0.3, 30 E% carbohydrate, and 10 E% protein. Based on the predicted Basal Metabolic Rate (WP 3) the energy content is fixed at 75% of the calculated 24 h BMR. Energy expenditure and respiratory quotient is measured by indirect calorimetry by an open-air circuit ventilated hood system during 30 minutes, preprandial and repeatedly in 1 hour blocks for 3 hours postprandially, standardization of both equipment and procedure will take place at each clinical center before the subjects are included. Pre- and postprandial (every 60 minute) blood samples are taken for determination of hormones (insulin, cortisol; only baseline measurements for: insulin-like growth factor 1, leptin), substrates and metabolites (glucose, free fatty acids, triglycerides, cholesterol, HDL-cholesterol, VLDL-triglycerides). All blood analyses will be performed in one laboratorium either by Partner No. 5 (Insulin, Leptin, Cholesterol, HDL-cholesterol, Glucose, Triglycerides, free fatty acids) or at a subcontracted commercial laboratory (Cortisol, insulin-like growth factor 1, VLDL-triglycerides). Subjects will score their hunger/satiety state on a visual analogue scale every hour pre- and postprandial. (Flint A et al, IJO,2000 Jan;24(1):38-48). Before and after the test meal a subcutaneous adipose tissue biopsy is taken from the abdominal region using a modification of the procedure described by Kolaczynski et al. (IJO 1994;18:161-6): After skin desinfection, the biopsy of abdominal subcutaneous adipose tissue is performed in the periumbilical triangle with a 2.6-mm-diameter needle (12G), after intradermal anesthesia with 500 µl 1% lidocaine. Adipose tissue is drawn by successive suctions into a 10 ml syringe containing 2 ml sterile saline solution. Compression of the area is then applied for 2 min. The adipose tissue samples are then shipped to Partner 11 and stored in a biobank until analysis (WP10 and WP11).

Deliverables

- D4.1. Obese study subjects to be transferred to the Dietary Intervention Programme (WP5).
- D4.2. Blood samples delivered to biochemical analysis as described in the WP4 and a biobank in Maastricht with Partner 5 and Subcontractor.
- D4.3. Blood samples delivered to sampling and processing of DNA and to a biobank in Copenhagen, Partner 3 (WP6).

- D4.4. Adipose tissue biopsy samples delivered to mRNA analysis and to a biobank in Toulouse, Partner 11 (WP10 and WP11)
- D4.5. Reports at Month 10 on results of each subject investigation, continuously transferred to the databank, 40% of the reports expected to be delivered to WP12.
- D4.6. Reports at Month 15 on results of each subject investigation, continuously transferred to the databank, 100% of the reports expected to be delivered to WP12.

Milestones
This workpackage is expected to result in a comprehensive and pertinent phenotypic characterisation of the study population with regard to anthropometry and body composition, and before and during a high-fat test meal challenge, circulating hormones, substrates and metabolites, thermogenic and nutrient partitioning capacity, and hunger/satiety scoring. Thereby the workpackage contributes, together with WP3, to *Milestone 2:* Single high-fat test meal challenge and synchronous clinical investigation following baseline assessments and a standardised diet run-in period.

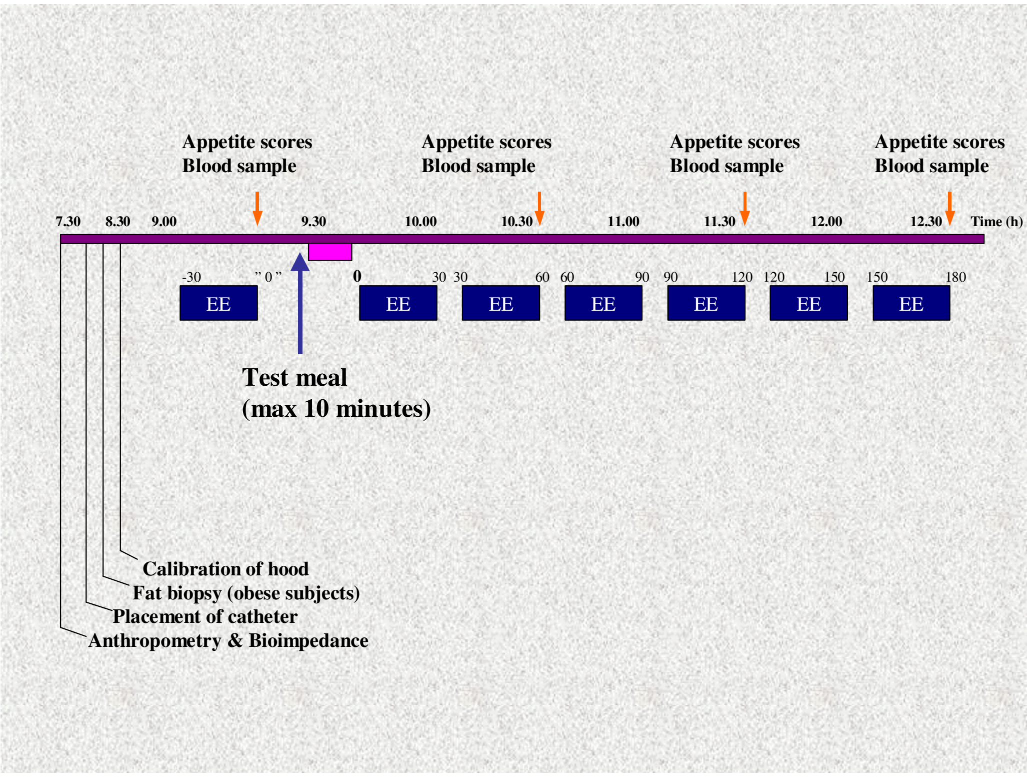

The figure above illustrates the timeframe and measurements of the CID. EE: Energy Expenditure.

After 10 weeks of dietary intervention, the following measurements were performed on the fasting obese subjects: Fasting body weight, waist and hip circumference, body composition, abdominal subcutaneous fat biopsies, and fasting blood sampling.

Measurement of energy expenditure in intervals of 20 minutes at the clinical day, Standardization of the method was secured by a standard operating procedure (SOP), followed by education when needed.

Modifications of the original WP plan

Composition of the test meal
Based on the pilot studies it was decided that the test meal should cover 50% of the estimated BMR instead of the originally foreseen 75% of BMR, and that the test meal should be double cream (for two centres double cream and butter, because of low fat content in the cream locally available). The energy distribution in the test meal is 95% fat, 3% carbohydrate and 2% protein.

Adipose tissue biopsy
Due to the difficulties in performing abdominal subcutaneous fat biopsy on lean reference subjects, and the increased risk of bleeding and damaging the underlying muscular structures, it was decided that abdominal fat biopsy should not be performed in these subjects.

Postprandial fat biopsy was only performed in a sub-sample due to the fact that it might not be comfortable for the subjects to have two biopsies on the same day.

Well being
The questionnaire on well-being following the test-meal was developed The use of this was optional, and the questionnaire was used in three of the clinical centres.

SOPs developed for WP4

A long list of SOPs was developed in relation to overall and specific aspects of the investigation procedure (all SOPs are available on the project website). The measurement procedures related to the clinical investigation day (CID) are:

Blood sampling and barcode system:
SOP Blood Sampling ***(Included in annex)***
Barcode_system.doc
Manual_bloodsampling.doc ***(Included in annex)***
Materials.doc***(Included in annex)***
Samplecodes.xls

Storage and shipment of blood and biopsy samples:
Boxinlay, box 1 steiner.xls
Boxinlay, box 2 steiner.xls
Boxinlay Buffy coat.xls
Boxinlay, plasmaserum.xls
Boxinlays.xls
cardform 1.xls
cardform 2.xls
SOP for storage
Fat biopsy:
SOP Needle Subcutaneous Fat Biopsy ***(Included in annex)*** (this was supplemented by a Video)
NUGENOB biopsies box inlay

NUGENOB biopsy handling protocol
NUGENOB SOP Adipose Tissue Preparation

Anthropometrics (body composition estimated by bioimpedance etc)
See WP2

Hood measurements
SOP Ventilated Hood System ***(Included in annex)***
Spreadsheet for Alcohol Burning
Spreadsheet for Repeated Human Measures

Test meal and Visual Analogue Scale scores (VAS-scores) (appetite, thirst)
SOP for VAS 0701
SOP Liquid Test Meal***(Included in annex)***

Overview
SOP for CID in lean reference subjects
SOP_Flowchart_CID_obese ***(Included in annex)***
SOP for the 2nd CID in obese subjects***(Included in annex)***
SOP_Flowchart_CID_lean
Timeline for CID***(Included in annex)***

Others
SOP for Dropout and Counting of Subjects ***(Included in annex)***
SOP for recording well being and vomiting during the CID (subproject) ***(Included in annex)***

Workpackage number: 5: Dietary intervention programme

Objectives
The objective is to assess responsiveness in the study population of 2 x 375 obese subjects with regard to body weight, body composition, and adipose tissue gene expression (WP10) to a hypo-energetic either low- or high-fat dietary 10-weeks intervention.

Methodology and study material
The 750 obese subjects of the study population, who have passed the Clinical Investigation Day (WP4), will be offered enrolment into a two-arm open label 10-weeks dietary intervention consisting of a hypoenergetic diet with either high fat/low carbohydrate or low fat/high carbohydrate content. Three days ahead of the Clinical Investigation Day, all subjects have been on a 3-day standard body weight maintenance diet (WP3). The intervention starts immediately after the Clinical Investigation Day (WP4) has been finalised and the subjects have been randomised to either the high-fat or low-fat diet. The subjects will receive a 3-hour dietary instruction in the diet programme by a trained dietician, and the instructions will be reinforced and body weight and composition controlled on a weekly basis until the end of the intervention. The aim is that both diets shall provide 600 kcal/d less than the individually estimated energy requirement based on the baseline measurement (WP4) multiplied by 1.3 to account for physical activity. The composition of dietary energy in the two diets will be: a) 20-25 en% fat, 15 en% protein, and 60-65 en% from carbohydrate; b) 40-45 en% fat, 15 en% protein, and 40-45 en% from carbohydrate. To enhance compliance to the diet the subjects will receive a dietary guideline developed at each participating Partner for this purpose. The food items will be purchased by the subjects themselves. Emphasis will be placed on the consumption of common local food items. Alcohol will be recommended to be a minimum. A 3-day weighed food record will be required after 3, 7 and 10 weeks. After 10 weeks the baseline measurements of fasting body weight and composition, fasting blood sampling (same as in WP4), and abdominal subcutaneous fat biopsies will be repeated (as in WP4). Seven Partners will manage 100 obese subjects and one Partner 50.

Deliverables
- D5.1. Report at Month 13 on results of each subject investigation, continuously transferred to the databank, 40% of the reports expected to be delivered to WP12.
- D5.2. Report at Month 19 on results of each subject investigation, continuously transferred to the databank, 100% of the reports expected to be delivered to WP12.
- D5.3. Blood samples delivered to biochemical analysis and a biobank in Maastricht, Partner 5 (Described in and part of WP4).
- D5.4. Adipose tissue biopsy samples delivered to mRNA analysis and to a biobank in Toulouse, Partner 11 (WP10 and WP11)

Milestones
This workpackage is expected to result in a clear characterisation of the obese study population with regard to how they respond in terms of body weight and composition and adipose tissue gene expression to a 10-week energy restriction low fat/high carbohydrate or high fat/low carbohydrate diet, which provides an important phenotypic trait in the study of nutrient gene interactions, and eventual differences in the effect of low or high fat energy restriction in obesity treatment. The workpackage thereby contributes to completion of *Milestone 3:* A 10-week hypoenergetic low-fat or high-fat dietary intervention programme for the obese study population.

Modifications of the original WP plan

Evaluation of the implementation of the dietary intervention during year 1:
It was reinforced that the major targets in the dietary intervention are to a) achieve a 600 kcal/day reduction in energy intake and b) achieve a difference in the fat energy percentage between 15 and 25. The targeted energy intake for the high fat group was 40-45 E% from fat, and the targeted energy intake for the low fat group was 20-25 E% from fat. When analysing data from the first (~250) subjects included in the intervention, it became evident that the low fat group tended to have a slightly higher fat intake that targeted, and that the high fat group tended to report slightly lower fat intake than targeted. Hereafter the centres were instructed to aim for a fat E% at 20% on the low-fat diet and at 45% on the high-fat diet. Thus, the overall targets and the principles for the dietary counselling were not changed, and the only modification was that the goal set for the subjects were modified.

The key SOP 'Dietary intervention proposal' was completed before the first subjects started the intervention. As shown in the SOP, minor amendments, related to additional targets and dietary assessments, were included in the early phase of the trial.

SOPs developed for WP5

SOP dietary intervention proposal ***(Included in annex)***
SOP for randomisation ***(Included in annex)***

Workpackage number: 6: Sampling and processing of DNA

Objectives

The objectives are to collect blood samples from participants of the study population undergoing the Clinical Investigation Day (WP4), to extract the genomic DNA, and to establish a common DNA bank to be used for 1) screening of candidate genes identified from WP7 for the presence of novel gene variants (WP8), and 2) genotyping the study population for selected gene variants.

Methodology and study material

Blood drawing and processing of blood samples will be performed according to the guidelines from The National Heart, Lung, and Blood Institute Working Group on blood drawing, processing, and storage for genetic studies (Am J Epidemiol 1996;144:437-41). In brief:

- From each subject enrolled in the Clinical Investigation Day programme (WP4), a total of 3 x 10 ml whole blood is drawn in sterile vacutainer tubes with ethylenediaminetetraacetic acid (EDTA). The blood samples are stored at room temperature.
- Within 3 days the samples are centrifuged at about 1500 x g for 30 minutes.
- The plasma is removed, and the white cell layer (the buffy coat) is transferred to cryovials (buffy coat from 2 x 10 ml EDTA blood to one cryovial, buffy coat from 1 x 10 ml EDTA blood to a second cryovial). The samples are then frozen at -80ºC.
- Samples are send to Partner 3 on dry ice, where genomic DNA is extracted from 2 x 10 ml EDTA blood by a modified salt precipitation method. Buffy coat from 1 x 10 ml EDTA blood is stored at - 80ºC.
- Extracted DNA samples are diluted in Tris/EDTA buffer to a stock DNA solution of 100 mg/ml and a working DNA solution of 20 mg/ml. Stock solutions are stored at -80°C, working solutions are stored at 4°C. DNA is stored and handled in locations free of contaminating PCR products.
- DNA samples are labelled by a study number in a person non-identifiable manner (see WP12). The key linking the study number and person identification data are only known and secured by the separate Partners collecting the samples.

Partner 3 will process 75 samples/month during a period of 12 months.

Deliverables

- D6.1. Report Month 9 on DNA samples in the DNA bank from the study population, to be collected and processed continuously during Month 3-15, 40% expected to be achieved. Delivered as confidential material to WP7, WP8, WP9 and WP12
- D6.2. Report Month 15 on DNA samples in the DNA bank from the study population, to be collected and processed continuously during Month 3-15, 100% expected to be achieved. Delivered as confidential material to WP7, WP8, WP9 and WP12.

Milestones

This workpackage results in establishment of a common DNA bank for the entire study population of obese and reference subjects. The workpackage thereby contributes, together with WP7-9, to completion of the *Milestone 4:* Molecular genetics studies on identification, analysis, and screening of putatively relevant, known or novel, nutrient-sensitive candidate genes, and *Milestone 5:* Genotypic screening of the entire study population for selected functional variants of putatively nutrition-sensitive genes.

Modifications of the original WP plan

The strategy for obtaining the buffy coat samples was adjusted from the original procedure described in the Techan document: Four tubes of 6 mL EDTA-blood have been drawn repeatedly at four time points during the examination day (time = 0, 60, 120, 180) and for the obese subjects also once after the completion of the 10 week weight loss intervention. One samples was initially used for DNA extraction. When the amount extracted was less than 25 µg two additional samples were pooled and DNA was extracted.

SOPs developed for WP6

Buffy coat ***(Included in annex)***

Workpackage number: 7: Identification of candidate genes

Objectives

The objective is to identify novel candidate genes involved in pathogenesis of obesity. Novel genes will be identified in collaboration between Partners 3 and 4, mainly on the basis of genetic linkage results obtained in obese families (positional candidates). Positional candidate genes will also be selected based on published chromosomal loci showing linkage to obesity. Selected novel candidate genes will be analysed for variants by WP8.

Methodology and study material

Linkage disequilibrium (LD) mapping. Linkage studies in Caucasian obese families have revealed several loci controlling BMI and leptin levels (3 'major' loci and more than 5 'minor' loci). Ongoing studies involving linkage analysis of 200 obese families with juvenile onset obesity (previously established as an independent project by Partners 4/13 and 10 outside the present study population) might confirm some regions and identify others. Fine mapping and LD mapping in obese families, trios, and cohorts of cases and controls will permit the definition of 1-5 cM regions, where the putative obesity genes map. Partner 3 is conducting a QTL mapping in another independent study of 63 Caucasian families with type 2 diabetes and obesity, which may identify loci controlling BMI, leptin levels and fat mass, relevant for the present application.
Selection of functional candidate genes. Within a linkage region of 5 cM, more than 100 genes may reside. Putative candidate genes will be identified in these regions showing linkage disequilibrium with obesity from public databases containing mapping information (for example Genemap at NCBI). Moreover, new candidate genes or ESTs (expressed sequence tags) will be selected based on information of their function or homology to other known genes. Gene products predicted to play a role in pathogenesis of obesity or for which the expression may depend on dietary fat exposures will be investigated.
Selection of differentially regulated genes. Using either cDNA microarrays or direct RT-PCR based techniques adipose tissue genes that are consistently regulated by the change in diet imposed on the participating subjects will be identified through wp10 and 11. It could be hypothesised that these genes play a significant role in adaptation to changes in nutrient intake, and therefore genetic variations in these genes as well as in other genes known to regulate these gene (such as transcription factors) may influence the degree to which individuals respond to changes in nutrient intake. Thus, gene products predicted to play a role in the pathogenesis of obesity or for which the expression may depend on dietary fat exposures will be investigated.
Cloning of relevant gene sequences. Cloning of human genomic sequences not available in genebanks, for example from cDNA/EST sequences identified through WP11, will be performed by placing cDNA-derived primers to identify DNA segments from human genomic DNA using PCR. A human genomic clone identified by PCR will be purchased from commercial suppliers and sequenced to determine intron-exon boundaries.
Functional characterisation of gene variants. Gene variants with predicted impact on the biological function of the gene product will be tested by relevant in-vitro transfection studies, using in-vitro cultured cell lines.

Deliverables

- D7.1. Report at Month 12 containing list of identified candidate genes based on: mapping linkage disequilibrium regions on chromosomes 10, 2, 5, and 20; definition of 1-5 cM regions where the putative obesity genes map; physical mapping in the 1-2 cM linked region. To be delivered to WP8, WP9 and WP10.

- D7.2. Report at Month 24 containing a list based on: screening of all gene banks for genes in the linkage disequilibrium region to find and test novel candidate genes; and clone full-length cDNA and gene organisation of novel genes. To be delivered to WP8, WP9 and WP10.

Milestones
This workpackage is expected to result in identification, cloning, sequencing and functional characterisation of a large number of novel putatively nutrition-sensitive, obesity-related candidate genes, from which selection for mutational analysis and genotyping of the study population may take place. The workpackage thereby contributes, together with WP6 and WP8-9, to completion of *Milestone 4:* Molecular genetics studies on identification, analysis, and screening of putatively relevant, known or novel, nutrient-sensitive candidate genes, and *Milestone 5:* Genotypic screening of the entire study population for selected functional variants of putatively nutrition-sensitive genes.

Modifications of the original WP plan
None

SOPs developed for WP7
None

Workpackage number: 8: Analysis of candidate genes

Objectives
The objective is to identify relevant functional variants of candidate genes, identified by WP7 and selected on the basis of their association with obesity, pathogenesis of obesity, or nutrient metabolism.

Methodology and study material
Selection of relevant candidate genes using bioinformatics. Based on knowledge of chromosomal regions showing linkage to obesity and related metabolic variables, available public databases will be searched for positional candidates within linkage areas. Identified positional candidate genes or ESTs will be prioritised based on their presumed functional role in relation to obesity, adipogenesis, and nutrient metabolism. Already identified single nucleotide polymorphisms (SNPs) will be identified in public databases.
Detection of gene variants by mutational analysis. Mutational analysis will be performed on candidate genes identified from WP7 and the present workpackage. Partner 3 will perform PCR-SSCP (single stand conformational polymorphism) and heteroduplex analysis at two different conditions as well as dHPLC analysis. Partner 4/13 will use and evaluate dHPLC and capillary sequencer equipment for mutation detection and sequencing. The dHPLC system from Transgenomic using the Temperature Modulated Heterozygote Analysis (TMHA) is a gel-independent system where samples are automatically loaded and analysed. The detected variants will be sequenced using automated sequencers to determine the nucleotide change.
Population to be subjected to mutational analysis. As screening basis we will initially use genomic DNA from subjects in the source populations examined in other settings (WP1), and after 2 months we will use obese subjects from the present study population. As source material we will use either genomic DNA isolated from subjects if the genomic structure of the candidate gene has been determined, or use adipose tissue derived cDNA (WP7 and WP11) for screening cDNA sequences for genes expressed in this target tissue. The purpose will be to screen for prevalent gene variants, i.e. SNPs, insertions, and deletions, associated with obesity per se or with the phenotypic measurements of WP3-5.

Deliverables
- D8.1. Report at Month 12 containing a list of gene variants corresponding to markers (SNPs and others) of relevant candidate genes. To be delivered to WP9.
- D8.2. Report at Month 18 containing a list of gene variants corresponding to markers (SNPs and others) of relevant candidate genes. To be delivered to WP9.
- D8.3. Report at Month 24 containing a list of gene variants corresponding to markers (SNPs and others) of relevant candidate genes. To be delivered to WP9.

Milestones
This workpackage is expected to result in the identification and characterisation of a series of variants of selected putatively nutrition-sensitive obesity-related candidate genes, which thereafter can be screened for in the entire study population. The workpackage thereby contributes, together with WP6-7 and 9, to completion of *Milestone 4:* Molecular genetics studies on identification, analysis, and screening of putatively relevant, known or novel, nutrient-sensitive candidate genes, and *Milestone 5:* Genotypic screening of the entire study population for selected functional variants of putatively nutrition-sensitive genes.

Modifications of the original WP plan
None

SOPs developed for WP8

None

Workpackage number: 9: Genotypic screening

Objectives
The objective is to perform genotyping in the total study population (750 obese group and 115 reference group) of a large number of functional variants identified in the candidate genes by Partner 3 and 4 (WP8). The resulting genotypes will be included in the common databank (WP12) used in the final analysis of interactions between genotypes and measured phenotypes of the study (WP3-5 and 10).
Methodology and study material
Variants of the putatively nutrition-sensitive candidate genes are selected for genotyping of the total study population. These gene variants are identified through 1) mutational analysis of novel genes generated by positional cloning (WP7); 2) mutational analysis of functional candidate genes (WP8); 3) mutational analysis of genes identified by expression profiling of adipose tissue mRNA (WP11); 4) the available pertinent knowledge about already known obesity-related gene variants. For example, variants in the melanocortin 4 receptor (MC4-R) may be the cause of obesity in 5-8 % of the obese cohort. In order to perform association studies relating gene variants with the phenotypic variables, the study population investigated by Partners 2, 5, and 6 (WP3-5) will be genotyped for a large number of gene variants, using relevant molecular genetic techniques. These could involve restriction fragment length polymorphisms (RFLP) analysis, micro sequencing, allele-specific oligonucleotide hybridisation (ASO), or restriction-site generating PCR (RG-PCR) in combination with RFLP analysis and could be performed on genomic DNA or on cDNA derived from adipose tissue biopsies (WP11). Partner 4/13 has established The INVADER™ Technology. This technology detects SNPs directly from genomic DNA without PCR. Most analysis will be performed in 96-well microtiter format, and DNA pipetting will be done using a pipetting robot. Genotyping data will be submitted to the data bank for performing the statistical analysis of interactions between genotype and phenotypes (WP12).

Deliverables
- D9.1. Report at Month 18 containing genotypes of the entire study population of variants in the selected candidate genes. To be delivered to WP10 and WP12.
- D9.2. Report at Month 21 containing genotypes of the entire study population of variants in the selected candidate genes. To be delivered to WP10 and WP12.
- D9.3. Report at Month 24 containing genotypes of the entire study population of variants in the selected candidate genes. To be delivered to WP10 and WP12.
- D9.4. Report at Month 27 containing genotypes of the entire study population of variants in the selected candidate genes. To be delivered to WP10 and WP12.
- D9.5. Report at Month 30 containing genotypes of the entire study population of variants in the selected candidate genes. To be delivered to WP10 and WP12.

Milestones
This workpackage is expected to result in complete genotyping of the study population of 750 obese and 115 reference subjects for all selected gene variants of the putatively nutrition-sensitive obesity-related candidate genes. The workpackage thereby contributes to completion of *Milestone 5:* Genotypic screening of the entire study population for selected functional variants of putatively nutrition-sensitive genes.

Modifications of the original WP plan
None

SOPs developed for WP9
None

Workpackage number: 10: Adipose tissue mRNA analysis

Objectives

The objective is to determine whether the expression of 40 putatively nutrient-sensitive genes in human adipose tissue from obese subjects is altered in response to a high-fat meal, to a long-term hypoenergetic low-fat diet and, to a long-term hypoenergetic high-fat diet.

Methodology and study material

Subcutaneous adipose tissue from the abdominal region is obtained by biopsy before and after a high-fat meal and after a long-term hypoenergetic low-fat diet or a long-term hypoenergetic high-fat diet (WP 2 #4-5) by Partners 2, 5, and 6b-12. The tissue is stored at -70°C by the clinical centres and delivered on dry ice to Partner 11. To increase the output from WP10, analyses will be performed by Partners 6a and 11 and a Subcontractor which is a newly created start-up company specialised in mRNA assays in human tissues. The three centres have a recognised expertise in the quantitative analysis of mRNA levels from limited amounts of adipose tissue. Total RNA is prepared using a standardised technique. Gene expression (defined as steady state mRNA levels) is quantified by solution hybridisation for highly expressed genes or quantitative reverse transcription PCR (RT-PCR) for genes expressed at low levels. We will select 4 subgroups of 25 obese subjects for each diet for the implementation of this workpackage. The partners will quantify on the different subgroups of subjects mRNA levels of 40 potentially nutrient-sensitive genes:

Partner 6a. Paracrine/secretory function of the adipocyte: Leptin, tumor necrosis factor-a, tumor necrosis factor-a receptors 1 and 2, angiotensinogen, plasmin activator inhibitor-1, interleukin-6, endothelial nitric oxide synthase, inducible nitric oxide synthase, ß-3 adrenergic receptor.

Partner11. Lipolysis: hormone-sensitive lipase, β_2-adrenoceptor, α_{2A}-adrenoceptor, phosphodiesterase 3B, G-protein αi2 subunit. Energy metabolism : uncoupling protein-2, nuclear respiratory factor 1, mitochondrial transcription factor A, cytochrome *c* oxidase subunits 2 and 4.

Subcontractor (of Partner 1). Lipogenesis: fatty acid synthase, acetyl coA carboxylase 1, glucose transporter 4, insulin receptor and insulin receptor substrate 1. Fat oxidation: carnityl palmitoyl transferase 1 and 2, lipoprotein lipase, fatty acid transporter 1, acetyl coA carboxylase 2, acylation-stimulating protein. Adipocyte differentiation: peroxisome proliferator-activated receptors α, β, γ1 and γ2, sterol-responsive element binding protein 1a and 1c, phosphatidylinositol 3-kinase p85α, p110α and p110β subunits.

The assays for these genes have already been validated by the Partners and the Subcontractor. The combined expertise of the Partners and the Subcontractor will allow to rapidly develop (1 month) and use assays for novel candidate genes with putative sensitivity to nutrition that will be discovered during the course of the project (WP11 and WP7-9).

Deliverables

- D10.1. Report at Month 12 containing quantitative gene expression data on 10 selected genes. To be delivered to WP12.
- D10.2. Report at Month 16 containing quantitative gene expression data on 10 selected genes. To be delivered to WP12.
- D10.3. Report at Month 24 containing quantitative gene expression data on 10 selected genes. To be delivered to WP12.
- D10.4. Report at Month 30 containing quantitative gene expression data on 10 selected genes. To be delivered to WP12.

Milestones

This workpackage is expected to result in the generation of 40 different quantitative mRNA assays for human adipose tissue, and characterisation of the dietary-induced changes in these candidate genes. The workpackage thereby contributes to completion of *Milestone 6:* Adipose tissue sampling and quantitative assessment of gene expression by mRNA assays.

Modifications of the original WP plan

None

SOPs developed for WP10

None

Workpackage number: 11: Gene-expression profiling in adipose tissue

Objectives

The objective is to identify genes in adipose tissue that respond to alterations of fat content of the diet in obese subjects using novel and advanced cDNA array technology. It is of particular importance to discover the nutrition-sensitive genes of human adipose tissue since this tissue is the major site for lipid storage and mobilization.

Methodology and study material

Subcutaneous adipose tissue will be obtained before and after a high-fat test meal and after a long-term hypoenergetic low-fat diet or a long-term hypoenergetic high-fat diet (WP 2 #4-5) by Partners 2, 5, and 6b-12. Tissue is stored at -70° C and shipped on dry ice to Partner 11. Expression of genes that either are increased or decreased following nutritional intervention will be identified in these adipose tissue samples. Pools of total RNA from 5 obese subjects will be used as probes after conversion of mRNA into cDNA and labelling. 50 pools will be prepared from 250 subject fat samples at different biopsy occasions. Probes will be hybridised to DNA chips consisting of cDNA clones micro-arrayed into glass slides or nylon-based cDNA arrays (Partners 6a and 11). Partner 11 will use commercially available DNA arrays for screening of 2000 genes. In parallel, Partner 11 will develop specific DNA chips that will contain probes corresponding to the main genes of metabolic pathways (lipogenesis, lipolysis, insulin signaling, transport and oxidation of fatty acids, glucose metabolism, thermogenesis and energy metabolism). Known transcription factors, nuclear receptors and co-factors involved (or suspected to be involved) in the nutritional control of intermediate metabolism will be included on the chip. Human cDNA clones will also be obtained by cDNA subtractions (representational difference analysis, RDA) by Partner 6a. RDA and commercial array systems for high throughput screening will identify a number of novel differentially regulated adipose tissue genes. Some of these genes may be unknown and will be selected for further analysis including cloning of full-length cDNAs. Highly validated specific DNA chips will allow a global view at nutrient-mediated regulation of genes important in metabolism. The changes in mRNA levels of newly discovered nutrient-sensitive genes will be confirmed on 25 subjects through the set-up, validation and use of specific quantitative RT-PCR assays as described in WP10. The analysis of genetic variations in novel genes identified in this WP, especially in regions known to regulate gene expression, will be done by Partners 3 and 4 according to methods described in WP7-9. At this stage, Partner 6a and Partner 11 have already made subtractive cDNA libraries and used cDNA arrays in other studies on human adipose tissue. It should be stressed that the micro-array part of the project is highly explorative using novel techniques, which for the moment are very resource demanding in terms of personnel and supply. On the other hand, unique information on gene-nutrient interactions can be obtained by these type of studies.

Deliverables

- D11.1. Report at Month 7 on development of the RDA and the DNA chips technology. Confidential or suitable for dissemination as scientific report dependent on achievements.
- D11.2. Report at Month 12 on completion of development of the technology. Confidential or suitable for dissemination as scientific report dependent on achievements.
- D11.3. Report at Month 18 containing a list of genes of which gene expression depends on the nutritional experimental manipulations. To be delivered to WP7, WP8, WP10 and WP12.
- D11.4. Report at Month 24 containing a list of genes of which gene expression depends on the nutritional experimental manipulations. To be delivered to WP10 and WP12.
- D11.5. Report at Month 32 containing a list of genes of which gene expression depends on the nutritional experimental manipulations. To be delivered to WP12.

Milestones

This workpackage is expected to result in the preliminary characterisation of several novel nutrition-sensitive genes using up-to-date functional genomics technique. This workpackage contributes to completion of *Milestone 7:* Adipose tissue gene expression profiling by cDNA arrays for nutrient-sensitive genes and of *Milestone 4:* Molecular genetics studies on identification, analysis, and screening of putatively relevant, known or novel, nutrient-sensitive candidate genes.

Modifications of the original WP plan

None

SOPs developed for WP11

None

Workpackage number:12: Databank and statistical analysis

Objectives

To establish at Partner 1 a common comprehensive databank including all relevant information on the study population delivered by the project activities by the other partners of the consortium (WP3-5 and WP9-10).

To set up a common professional statistical analysis facility, which is capable of effectively and adequately analysing the data in the databank as determined by the specific research questions posed. The statistical techniques used will be standard techniques as available or such techniques modified by statisticians in accordance with the experimental design of the study and structure of the data.

Methodology and study material

The subjects entered into the study population by selection from the source populations (WP1-2) will be assigned a unique study number linked to the key providing the person identification (full name, address, date of birth, local person identification no.) only at the partners recruiting the subjects. For each workpackage delivering data on these subjects, a local database receiving the data by subject study number will be set up at the site of production. The database will be using similar database software at all partners. There will be established local effective and regular back-up procedures. At regular intervals the data of the database will be transmitted by e-mail to the *central databank* established according to exactly the same rules, thereby allowing easy updating and concordant data cleaning and error corrections. Data will be delivered from the central databank as needed for the statistical analysis.

The fundamental tasks of the *statistical analysis* are the comparisons of pertinent quantitative phenotypic traits (including gene expression measures) between obese subjects with different genotypes (gene variants) in putatively nutrient-sensitive candidate genes and between these different groups of obese subjects and the reference group of subjects. These phenotypic traits may be any results of the clinical investigation and the subsequent dietary intervention program for the obese, and any result of the clinical investigation of the reference group. Distributional assumptions of the statistical methods will be checked and transformations performed as appropriate. Results will be analysed using stratification by centres and adjustment for basic demographic characteristics and *ad hoc* relevant confounders. Single point measurements will be assessed by mean differences and t tests, and repeated time course measures will be modelled and compared by covariance analysis. Significance levels will be adjusted for multiple comparisons. These analyses will be conducted and reported under supervision by a professional statistician at Partner 1.

Deliverables

- D12.1. Report at Month 3 describing the structure of and the interaction with the central databank. Confidential, to be delivered to all Partners.
- D12.2. Report at Month 6 containing descriptive statistics on the accumulated information in the databank. Confidential, to be delivered to all Partners.
- D12.3. Report at Month 9 containing descriptive statistics on the accumulated information in the databank. Confidential, to be delivered to all Partners.
- D12.4. Report at Month 12 containing descriptive statistics on the accumulated information in the databank. Confidential, to be delivered to all Partners.
- D12.5. Report at Month 15 containing descriptive statistics on the accumulated information in the databank. Confidential, to be delivered to all Partners.
- D12.6. Report at Month 18 containing descriptive statistics on the accumulated information in the databank. Confidential, to be delivered to all Partners.

- D12.7. Report at Month 21 containing descriptive statistics on the accumulated information in the databank. Confidential, to be delivered to all Partners.
- D12.8. Report at Month 24 containing descriptive statistics on the accumulated information in the databank. Confidential, to be delivered to all Partners.
- D12.9. Report at Month 27 containing descriptive statistics on the accumulated information in the databank. Confidential, to be delivered to all Partners.
- D12.10. Report at Month 30 containing descriptive statistics on the accumulated information in the databank. Confidential, to be delivered to all Partners.
- D12.11. Report at Month 33 containing descriptive statistics on the accumulated information in the databank. Confidential, to be delivered to all Partners.
- D12.12. Report at Month 27 on results of statistical analysis of the accumulated data. Confidential, to be delivered to assigned authors of scientific reports for dissemination of the study results.
- D12.13 Report at Month 29 on results of statistical analysis of the accumulated data. Confidential, to be delivered to assigned authors of scientific reports for dissemination of the study results.
- D12.14. Report at Month 31 on results of statistical analysis of the accumulated data. Confidential, to be delivered to assigned authors of scientific reports for dissemination of the study results.
- D12.15 Report at Month 33 on results of statistical analysis of the accumulated data. Confidential, to be delivered to assigned authors of scientific reports for dissemination of the study results.
- D12.16 Report at Month 35 on results of statistical analysis of the accumulated data. Confidential, to be delivered to assigned authors of scientific reports for dissemination of the study results.

Milestones

This workpackage is expected to result in establishment of local databases and central databank receiving data from the investigations of the entire study population, and subsequent series of statistical reports that addresses the key scientific questions derived from the objectives of this project. The workpackage thereby contributes to completion of *Milestone 8:* Collection and integration of the generated data and conduct of statistical analysis of these data.

Modifications of the original WP plan

None

SOPs developed for WP12

SOP for data entry and data transfer ***(Included in annex)***

3. ROLE OF PARTNERS

This Chapter was redundant, and has been deleted for convenience reasons

4. PROJECT MANAGEMENT AND COORDINATION

Decision-making structure

The decision-making structure of this project will be based on allocation of responsibility for conduct of the workpackages among the partners, on a Steering Committee, a project Co-ordinator with three assistant co-ordinators, and a pre-specified management structure. The function of each of these components is described below. A written and signed Consortium Agreement including specified allocation of responsibility will be made to secure the effectiveness of the decision-making structure.

Partners, Steering Committee, and project Co-ordinator

The *partner responsible* for the content of each of the workpackages will also be responsible for the implementation, standardization, and quality assurance of the workpackages at the group level, which will include *on site* supervision of particular sensitive procedures (dietary assessment, metabolic investigation, fat tissue biopsy procudure, dietary instructions. At each of the partner centres, there will only be one senior researcher, who carries the local responsibility for the implementation of the workpackage(s) assigned to the partner, but temporary responsibility may be delegated to a local substitute.

Decision-making on any important aspect of the project will be made by a *Steering Committee*. Any aspects of the projects without influence on the integrity of the project as such may be dealt with within the centres of the partners under the responsibility of the Steering Committee member. This Steering Committee consists of 12 senior researchers, one from each of the partners 1 – 12, who are responsible for the performance of the workpackages, and it will be chaired by the representative for Partner 1, the project Co-ordinator. A substitute of the Co-ordinator as chairman of the Steering Committee will be assigned on an ad hoc basis.

The Co-ordinator will be assisted by three *assistant co-ordinators,* one at the PostDoc level of clinical and genetic epidemiology expertise to be employed full time throughout the study at Partner 1, and two employed as members of permanent/local staff at the departments of other partners in Copenhagen: an Associate Professor (Dr Soeren Toubro) from Partner 2 with special expertise in the clinical investigation components, and a PostDoc researcher (Dr Soeren Echwald) from Partner 3 with special expertise in the molecular genetics components of the project. The assistant co-ordinators are ex officio members of the Steering Committee.

A major task of the Steering Committee will be to make decisions on patenting, licensing, and publication strategies including sharing of the merits and royalties. With regard to the share of merits by authorship of the scientific papers, the Steering Committee will aim at adhering to the rules set forth in the Consortium Agreement.

Management structure

The Co-ordinator will be responsible for: a) management of the overall strategic direction of the project; b) liaison with the Commission Services through the assigned Scientific Officer, Dr Barend Verachtert; c) co-ordination of the meetings, monitoring progress towards targets

collation of the scientific reports, and minutes of the meetings; d) submission of the technical reports and other reports, distribution of the financial reports, and programme evaluation. The flow of deliverables, with those to and from WP12 as the core ones, will be the basis for monitoring the progress of the work including supervision and feed-backs. The co-ordinator will be assisted in performing these tasks by the local assistant co-ordinator and the local administrative staff of the Partner 1.

The day-to-day decision-making by the Co-ordinator is based on input from all partners: an initial decision is to be taken by the Co-ordinator after consultation with the assistant co-ordinators according to their respective expertise and any relevant Steering Committee members. After communicating the provisional decision to all partners and following and adapting to any feedback, a final decision will be taken and communicated to all partners.

The Co-ordinator and the assistant co-ordinators will have a Co-ordination office at Partner 1 to assist in the co-ordination and steering activities. This Co-ordination Office will be supported by the local administrative staff on part-time basis and will include an experienced administrator and a professional secretary who are able to communicate in English. Local meetings with the Co-ordinator, the staff and the assistant co-ordinators will be held at least monthly.

Meetings of the Steering Committee

In order to present and discuss the current status and possibly to decide on any adjustments of the project on the basis of the very first practical experiences of workpackage implementations by the partners, the Steering Committee will have pre-scheduled physical meeting on the following occasions (conf. the Gantt-chart):

1. Early in the preparatory phase, month 1 (WP1-2).
2. Shortly after the first series of patients have passed the clinical investigation day, month 3 (WP4).
3. Shortly after the first series of patients have finished the dietary intervention programme, month 6 (WP5).
4. Shortly after the start-up of the mRNA analyses of the fat biopsies, month 8 (WP10-11).
5. Shortly after the start-up of the genotypic screening, month 15 (WP9)
6. Shortly after the start of statistical analysis of the database, month 18 (WP12).

Meetings 2, 3, 5 and 6 may be plenary meetings involving relevant members of the involved staff at each partner, whereas meetings 1 and 4 may be limited to the Steering Committee. In order to maximise the competent utilisation of the collected data, the Steering Committee will have regular physical meetings every other month during the last 6 month of the project, i.e. months 30, 32, 34 and 36, and the first and the last of these meetings will be plenary whereas the two in between are only for the Steering Committee. During these meetings, it is expected that statistical reports prepared by the statistician at Partner 1, and draft manuscripts of scientific articles will be presented and discussed assuming that they will be forwarded to the Steering Committee members about one month before the meetings.

The physical meetings will be hosted by the partners in their respective cities and organised in collaboration with the Co-ordination Office according to the following schedule:

1) Copenhagen, Denmark
2) Maastricht; The Netherlands
3) Lille, France
4) Toulouse, France

5) Nottingham, UK
6) Prague, The Czech Rep.
7) Pamplona, Spain
8) Stockholm, Sweden
9) Paris, France
10) Copenhagen, Denmark

Any of the members of the Steering Committee may request that a problem in the project is so important that it has to be discussed at a meeting in the Steering Committee. The Steering Committee will aim at consensus-based decision-making following thorough discussion. If consensus cannot be achieved, the decision is made by majority voting with the votes of the committee chairman being decisive at balance. If such decision is unacceptable to a partner of the consortium, the Commission Services will be consulted. Meetings of the Steering Committee that are not pre-scheduled may be held as telephone conferences or physical meetings at the choice of the chairman.

Any meeting of the Steering Committee will be performed on calls followed by an agenda sent out from the Co-ordination Office to the committee at least 8 weeks in advance for the members to prepare their participation in the meeting. Every pre-scheduled meeting of the Steering Committee will have as standard agenda to receive a status report from the Co-ordination Office, both on co-ordination businesses, the flow of deliverables, as well as the data flow to the database (WP12).

Subgroups among the Steering Committee members may assemble ad hoc to solve specific problems in the implementation of selected workpackages into which they are involved, and these meetings should be co-ordinated by those responsible for the workpackages in question and be co-ordinated through the Co-ordination Office, with participation of the assistant co-ordinator whose area of expertise covers the problem to be discussed.

Any of the members of the Steering Committee may decide to let a substitute from the partner act under instruction on his/her behalf at any single meeting of the committee.

Minutes will be prepared at the earliest convenience by the assigned assistant co-ordinators of the meetings with the support by the Co-ordination Office. Minutes including decisions on important matters will not be effective until the committee has approved the minutes. The minutes are considered approved if no corrections have been received a week after the delivery of the minutes.

Communication flow within the consortium

The Co-ordination Office of Partner 1 will be responsible for an effective communication flow within the consortium using communication medium as appropriate. All partners are connected by e-mail, so all information can be copied to all partners.

At each partner a named part-time secretary will be allocated to the communication activities and any other local secretarial businesses.

The Co-ordination Office is responsible for selective dissemination of information according to relevance criteria for the receiver of the information. With regard to the communication flow of the research data produced by the project, see description in Workpackage WP12.

5. EXPLOITATION AND DISSEMINATION ACTIVITIES

The longterm goal of exploitation and dissemination of the results of the project depends on the ability of the project to generate new insight into the role of fat intake in development, prevention and treatment of obesity. If this is achieved, then it may contribute to the basis for new dietary guidelines. The results of the project may form the basis for development of new diagnostic tools and drugs for treatment and prevention in targeted high-risk groups may open new avenues for the health care sector in management of the clinical problems of obesity.

Any major new discovery of the consortium will be carefully evaluated with respect to the potentials of securing patents or granting licenses by the Steering Committee under strict confidentiality. Patent offices at the host institutions of the partner(s) where the most significant contribution to the findings are generated will be contacted and asked to help in evaluating the potentials. The signed Consortium Agreement will include internal rules about the procedures and possible shares of patents and licenses.

After the internal evaluation of potentials for commercial exploitation as appropriate, the results of the project will be reported to the international scientific community dealing with nutrition, genetics and obesity. This dissemination will, as is common practice, take place in two phases, first presentation and discussion of the results at relevant conferences and congresses, then publication in international, peer-reviewed scientific journals. The allocation of authorship responsibility will adhere to the latest version of the Uniform requirements for Manuscripts submitted to the Editors of Biomedical Journals (The “Vancouver” Rules). The research consortium will under the name ‘NUGENOB’ appear as last author in all publications and a footnote will list all partners and describe their respective contributions. The signed Consortium Agreement will deal with these matters.

When the results have been communicated to the international scientific community, then the next phase of dissemination of the results will be reporting by the partners in local scientific journals targeting the local health sector and magazines oriented toward the interested general public. Several of the partners of the consortium collaborate with the pharmaceutical industry actively involved in development of treatment for obesity. The results from the project will be presented to the research bodies in the respective companies. If the generated knowledge has not been protected, the conclusions can be used freely by the industry and their scientist after the results have been published in scientific journals.

The project involves two SME’s (small and medium-sized enterprises), namely Artsen Laboratorium Dr. Th. Stein (Medical Laboratory of Dr. Th. Stein) in Maastricht, The Netherlands, and EZUS-LYON1/Genalys, Lyon, France. Both companies will benefit from the expansion of their networks of expertise throughout the participating countries of the European Community in which the partners of the projects reside. The Medical Laboratory of Dr. Th. Stein is a highly experienced laboratory conducting specialized biological analysis on blood samples, and EZUS-LYON1/Genalys is a newly created start-up company specialised in mRNA assays in human tissues.

Several of the partners of the project are directly involved in treatments and advisory functions for obese subjects needing or requesting weight reduction. Results from the project will be exploited within these centres to improve the management of the obese patients seeking help. The same partners also have advisory functions for the general public both in terms of being

opinion leaders and by contributing to formulation of dietary recommendations for government institutions, and useful results will be disseminated through these channels as well.

In order to make the results available in a broader context, a public symposium will be held in connexion with the last planned meeting of the Steering Committee in Copenhagen in Month 36. The symposium invitation profile will be dealt with by the Steering Committee and the Commission Services. European health care professionals, health care manager, policy makers, representatives of the pharmaceutical and food industry, and consumer representatives will be invited to this symposium, not only from the countries in which the study has been conducted. Presentations in the workshop will be on a high level, but understandable for non-scientist. During the workshop conclusions of the project - particularly any potential implications for changes in dietary guidelines - will be presented and discussed. The conclusions from the symposium will be made available for the general public via a press conference and PR-activities carried out by the participating industrial partners.

After the project has come to an end (Month 36), there will be a potentially extremely valuable material left in the local and central biobank (DNA, blood samples, adipose tissue samples) and the local and central databank. The continued exploitation of this material is worthwhile and desirable also from the European Community point of view. In order to secure proper exploitation respecting the intellectual properties of the partners, the decision-making structure will be maintained after the cessation of the project and this will be incorporated in the signed Consortium Agreement.

6. ETHICAL AND SAFETY PROVISIONS

The partners of this project commit themselves to abide by all principles expressed in relevant international text or codes or practices, i.e. the latest version of the Declaration of Helsinki, the Convention of the Council of Europe on Human Rights and Biomedicine and the UNESCO Declaration on the human genome.

The proposal supplemented by a detailed ethical account from each partner has been subject to Ethical Review via the Commission Services and the following address all relevant ethical issues.

Ethical aspects

This project does involve :

- Use of human tissue (adipose tissue biopsies)
- Research on persons (subject members of the study population)
- Research on healthy volunteers (reference subjects)
- Collection of genomic DNA and adipose tissue cDNA.

The project does not involve :

- Human embryos or foetuses
- Use of human embryonic or foetal tissue

- Research on children
- Research on persons unable to consent
- Research on pregnant women
- Use of non-human primates
- Use of transgenic animals
- Use of other animals
- Genetic modification of animals
- Genetic modification of plants

Procedure and authorized centers for clinical investigation

Investigations on obese subjects and healthy volunteers will be performed in authorized clinical investigation centers. During the preparatory phase of the project, the detailed workplan will be submitted to the legally authorised scientific and ethics committees giving permissions to this kind of research within each of the member states in accordance with member state laws. The source populations are already included in other local projects, which are based upon the permissions from the local ethics committees.

WP7 mention research activities that are based on analysis of DNA extracted from blood samples. These are obtained in ongoing separately ethically approved studies of the genetics of obesity in Paris, France and Copenhagen, Denmark in which family members individually volunteered to provide the blood samples for the research purposes after informed consent and secured confidentiality. The role of these WP7 activities in the present project is only to supply the present project with essential information on the results of the search for candidate genes or genomic loci associated with obesity. The subjects will not be contacted again as part of this project.

It is not expected that local national permission to perform the present study will cause any difficulties. Similar kind of projects, including work on genetic information from their DNA, with similar ethically challenging components have been approved in each of the member states of the partners of the project.

Study population and adipose tissue biopsy

Caucasians (by self-report) of both genders between the ages 20-50 years will be enrolled. 100 obese subjects and 15 healthy volunteers per centres (with the exception of one centre, the one in Toulouse where only half the patients will be enrolled) will be selected and appointed for the program. The basic selection criteria for both the obese and the reference subjects will be that they provide informed consent, are non-pregnant, not abusing alcohol or drugs, and are feeling so healthy that they are capable of working full time. The women should be pre-menopausal, and any use of oral contraceptives will be recorded and taken into account in the analysis. Specifically, the subjects shall not suffer from clinically diagnosed and treated diabetes, hypertension, hyperlipidaemia, thyroid diseases, or other diseases, which by themselves or via their treatments are suspected of influencing the results of the investigations.

Microbiopsies of adipose tissue have been performed in all centers involved in the project, and some of the centers (partner numbers 6 in particular) have longstanding and very broad experience in the procedure. A standardized procedure will be used. After skin desinfection, the biopsy of abdominal subcutaneous adipose tissue is performed in the periumbilical triangle with a 2.6-mm-diameter needle (12G), after intradermal anesthesia with 500 µl 1% lidocaine. Adipose

tissue is drawn by successive suctions into a 10 ml syringe containing 2 ml sterile saline solution. Compression of the area is then applied for 2 min. Performed in this way, the procedure causes little and very limted pain if any.

Informed consent, protection of and information about personal data

The subjects will only be accepted for participation in the study if they have provided informed consent. When informing the subjects before consent, it will be emphasised that participation is without any obligations and that they can withdraw from the study at any time, and that they will not suffer in any way from such withdrawal.

Written informed consent will be obtained from all subjects. The form describes in detail the clinical investigations and possible risks (mild pain associated to venous catheterization and local anaesthesia; limited superficial haematomas during local anaesthesia and needle subcutaneous adipose tissue micro-biopsies; possibilities of local infection) as well as the procedures to limit the risks.

It also specifies that all personal data are confidential. In respect of legal procedures in each country, subjects may receive an indemnification for the participation in the project: the obese subjects, who will be offered the 10 weeks dietary intervention will receive 50 Euros, and the healthy volunteers, who are not offered any treatment services, 150 Euros. In addition, their transportation expenses will be reimbursed.

The subjects will be informed about personal data if they request the information. It will be specified in the informed consent form that the subjects face therefore the risk to obtain a piece of information that may contribute to the diagnosis of a disease.

If the investigation reveals hitherto undiagnosed diseases that need treatment, the subjects will be encouraged to seek assistance at the local health services. The risks are extremely limited in this project because none of the biological and genetic data collected are known to be predictive for severe unexpected and undiagnosed diseases (i.e., other than obesity). Subjects with diagnosed diseases will be excluded during selection procedure (see above).

With regard to protection of personal data, the legislation setting the regulations of the handling of personal data in each member states will be adhered to and, where appropriate, permissions will be sought from the national data protection authorities.

Each subject will be assigned a project serial number that will be assigned to all information and all samples drawn from the subjects. The key linking this serial number to the person identification will be kept covert and secure at the primary investigation centers. Only encoded information on each subject enrolled into the study population will be transmitted for data analysis to Partner 1.

The present study may result in discovery of gene markers that predict obesity or risk of obesity in the subjects carrying the gene variants. The ethical aspects of recommendations to general use of such markers in the public will have to be carefully considered with due respect to the trade-off between improved possibilities for treatment and prevention versus the risk of stigmatization of the carriers of the gene variants.

Any transmission of genetic or non-genetic information, following requests about the individual results, will only take place in the context of secured appropriate counseling.

Safety provisions

The staffs of the Clinical Investigation Centres are trained and experienced in clinical trials according to Good Clinical Practice. Procedures for rapid intervention of first emergency aid have been planned. All handling of blood will be performed under consideration of the potential risk of infection from the study subjects possibly carrying contagious diseases.

All experiments and research work described in the proposed project will be performed following standards of Good Laboratory Practice based on national guidelines. Whenever appropriate, the laboratories are authorized to handle human adipose tissue, perform total RNA preparation, RNA analysis (quantitative RT-PCR, cDNA array and chip technology), genomic DNA preparation and DNA analysis.
