# Supplementary material for: Genetic Polymorphisms and Weight Loss in Obesity: A Randomised Trial of Hypo-Energetic High- versus Low-Fat Diets
Source: PLoS Clin Trials. 2006 Jun 30;1(2):e12. doi: 10.1371/journal.pctr.0010012 (PMC1488899; doi:10.1371/journal.pctr.0010012)
Supplement: Annexes to the Trial Protocol [file pctr.0010012.sd003.pdf]

"Nutrient-Gene Interactions in Human Obesity: Implications for Dietary Guidelines"
is a European Community project under the programme:
"Quality of Life and Management of Living Resources - Key Action I: Food, Nutrition and Health"

NUGENOB
QLRT-2000-00618
www.nugenob.org

Annex to the Nugenob Protocol – Selected relevant SOPs

WP2

SOP for Inclusion and Exclusion (including medication list)
SOP for anthropometry

WP4

<u>Bloodsampling and barcode system:</u>
SOP Blood Sampling
Manual for bloodsampling
Materials

<u>Fat biopsy</u>
SOP Needle Subcutaneous Fat Biopsy

<u>Hood measurements</u>
SOP Ventilated Hood System

<u>Test meal</u>
SOP Liquid Test Meal

<u>Overview</u>
SOP_Flowchart_CID_obese
SOP for the 2nd CID in obese subjects
Timeline for CID

<u>Others</u>
SOP for Dropout and Counting of Subjects
SOP for recording well being and vomiting during the CID

WP5

SOP Dietary assessment and intervention
SOP Randomisation

WP6

SOP Buffy Coat

WP12

SOP for data entry and data transfer

NUGENOB

SOP for Inclusion and Exclusion of Subjects

General inclusion/exclusion criteria	Page 2
Guidelines for subjects suffering from thyroid disease	Page 3
Drugs Allowed and Drugs Not Allowed	Page 4

General inclusion/exclusion criteria

IN GENERAL:
- **CONFER** with a coordinator if you have any doubts about inclusion.
- **MAKE A REMARK** in the comments whenever there is anything special to report about a subject.

Obese subjects

Inclusion criteria

1) BMI >= 30 (without any upper limit)
2) Intention to include white Europeans (exclusion non-whites by self-report)
3) Age 20-50 years (both 20 and 50 year olds can be included)
4) Pre-menopausal women (women having *regular* menstruations)

Exclusion criteria

5) Drug-treated hypertension
6) Drug-treated diabetes
7) Drug-treated hyperlipidaemia[1]
8) Drug-treated thyroid diseases
9) Use of prescription medication marked with N on medication list below
10) Surgically treated obesity
11) Participation in ongoing drug trials
12) Pregnant women
13) Alcohol or drug abuse (based on clinical judgment)
14) Weight change of > 3 kg within 3 months prior to Clinical Investigation Day

Reference (lean) subjects

The reference subjects should be recruited such that they reflect the obese in sex and age.

Inclusion criteria

1) BMI 18.5-25.0
2) Intention to include white Europeans (exclusion non-whites by self-report)
3) Age 20-50 years
4) Pre-menopausal women (women having *regular* menstruations)
5) Healthy and no regular medication (exception oral contraceptives)

Exclusion criteria

6) Weight history with BMI >25, except pregnancy weight
7) Participation in other clinical studies within the last 3 months
8) Pregnant women
9) Alcohol or drug abuse (based on clinical judgment)
10) Weight change of > 3 kg within 3 months prior to Clinical Investigation Day

[1] See guidelines for subjects suffering from thyroid disease (page 3)

Guidelines for subjects suffering from thyroid disease

Having followed the various arguments pro et con, following has been decided regarding subjects suffering from thyroid disease:

1) Patients who suffer from HYPERthyorid disease are not allowed in the study, even though they may be well treated by drugs, such as methylthiouracil.

2) Patients who have a recently diagnosed, not yet completely stable, HYPOthyroid disease are not allowed in the study.

3) Patients who suffer from a longstanding, stable HYPOthyroid disease, well treated by thyroxin substitution are allowed to be included* (This includes hypothyroidism as a sequelae to definitive treatment of hyperthyroidism by surgery or radioactive iodine).

* Pleas note that because it is still possible to include patient of category 3) making an appropriate comment on the special case in the CRFs.

Drugs Allowed (Y) and Drugs Not Allowed (N) as Concomitant Medications*

No anti hypertensive medications incl. beta blockers is allowed (according to WP2).

All allowed medication must have been prescribed on a stable dose for at least 2 months prior to the clinical day. In general no episodic medication is allowed on the day before the clinical day.

Drug Class	Episodic Use	Chronic Use
Anorexigenic agents**	N	N
Antiepileptic drugs	N	N
Anti-Parkinsonian drugs	N	N
Barbiturates	N	N
Benzodiazepines	Y	N
Beta- blokkers	N	N
Butyrophenones	N	N
Carbonic anhydrase inhibitors (e.g., acetazolamide, dichlorphenamide, dorzolamide, methazolamide)	N	N
Diuretics (thiazid and loop diuretics)	N	Y
Dopamine reuptake inhibitors (e.g. bupropion)	N	N
Digoxin	N	Y
Fibrates	N	N
Fish oil supplement	N	N
Glucocorticoids	N	N
Heparin	N	N
Immunosuppressives	N	N
Insulin	N	N
Laxatives (non-fiber preparations)	Y	N
Monoamine oxidase (MAO) inhibitors	N	N
Niacin (>150 mg/day)	N	Y
Nicotine (e.g., gum, patch)	N	Y
Oral hypoglycemics	N	N
Orlistat	N	N
Phenothiazines	N	N
Serotonergic agents	Y***	Y
Selective serotonin reuptake inhibitors (SSRIs)	N	N
Serotonin and norepinephrine reuptake inhibitors (SNRIs)	N	N
Statiner	N	N
Thyroid hormone	N	Y****
Triamterene	N	N
Tricyclic antidepressants	N	N
Warfarin	N	N
Zonisamide	N	N

* Drugs and Drug Classes not specified are generally allowed. Further questions should be directed to st@kvl.dk.

** Including, but not limited to, St. John's Wort, phentermine, fenfluramine, hypericum, and phenylpropanolamine

*** Episodic migraine treatment

**** See guidelines for subjects suffering from thyroid disease (page 3)

SOP for waist/hip ratio.

Waist circumference.

The subject wears little clothes so that the tape may be correctly positioned. The measurement should not be made over clothing.
The subjects stands erect with the abdomen relaxed, the arms at the sides and the feet together. The measurer faces the subject and places an inelastic tape around the subject, in a horizontal plane, in the mid-way between the lowest rib and the iliac crest.
An assistant is needed to help position the tape in a horizontal plane.
The measurement should be taken at the end of a normal expiration, without the tape compressing the skin. It is recorded to the nearest mm.

Measurement is done two times but seperated by hip circumference measurement.

Hip circumference.

The subject should wear only nonrestrictive briefs or underwear. The subject stands erect with arms at the sides and feet together. The measurer squats at the side of the subject. Measurement takes place at the level of the greater trochanter without compressing the skin. An assistant is needed to help position the tape on the opposite side of the subject's body. The measurement is recorded to the nearest mm.

Measurement is done two times but seperated by waist circumference measurement.

Formula: WHR = waist circumference / hip circumference.

Reference:

* Lean MEJ, Han TS, Morrison CE. Waist circumference as a measure for indicating need for weight managment. BMJ 1995;311: 158-61
* Han TS, van Leer EM, Seidell JC, Lean MEJ. Waist circumference action levels in the identification of cardiovascular risk factors: prevalence study in a random sample. BMJ 1995;311: 1401-5.

SOP for body height and body weight.

Body height.

Body height is measured with a calibrated stadiometer.
The subject stands erect with the abdomen relaxed, the arms at the sides and the feet together. Measurement is done three times to the nearest cm..
The mean of the three measurements should be written down as the body height.

Body weight.

Body weight is measured with a calibrated scale.
The subject stands still over the center of the platform with the bodyweight evenly distributed between both feet. Subjects have to be weighed in underwear without shoes. Body weight is measured to the nearest 0.1 kg after the subject holds his breath shortly after a normal expiration. Measurement is done three times.
The mean of the three measurements should be written down as the body weight.

Determination of substrates and hormones in plasma

Recommendations for collection, processing and storage of specimen.

Don't throw away the tubes after plasma collection. The buffy coat is also needed!

Collection

Tubes: 3x vacutainer (4ml, 7.2mg K2 EDTA), Becton Dickinson, ref.no. 368861

- Chill the collection tubes on ice prior to sampling
- Draw at least 4 mL blood in each tube
- Store the tube on ice until plasma can be seperated

It is imperative that no heparin is in the blood sample. Draw at least 2 ml of blood from the catheter in a syringe prior to blood sampling. This can be thrown away.

Processing

- Centrifuge the tube as soon as possible after collection for 15 minutes at 1200g (3650 RPM), 4° C.
- Transfer the plasma to a clean, labeled cryovial. *Leave the phases close to the buffy coat (over and below)*
  (Microtube with cap, 2ml. Sarstedt BV,. ref.no. 72.694)

T = 0: Put 0.5 ml plasma in 9 cryovials with the barcode **80 XX XXX 1 EDTA**

T = 60: Put 0.5 ml plasma in 9 cryovials with the barcode **80 XX XXX 2 EDTA**

T = 120: Put 0.5 ml plasma in 9 cryovials with the barcode **80 XX XXX 3 EDTA**

T = 180: Put 0.5 ml plasma in 9 cryovials with the barcode **80 XX XXX 4 EDTA**

T =10 wks: Put 0.5 ml plasma in 9 cryovials with the barcode **80 XX XXX 5 EDTA**

*** Buffy coat:** See the SOP for processing of buffy coat.

Storage

- Store the plasma at < -70° C
- Ship on dry ice (organized by UM)
- Avoid repeated freeze-thawing

Store the cryovials in the boxes the way as described in the SOP, storage blood samples!

Buffy Coat

Recommendations for processing and storage of specimen.

Processing

- After transfer of the plasma, take the buffy coat between the plasma and the blood (this is a small white layer) with a small pipet (P100).
  It doesn't matter if you have a bit of plasma or blood with it.

- Transfer the buffy coat of two or three tubes in one cryovial.
  (Microtube with cap, 2ml. Sardstedt BV. ref. no. 72.694)

*** T = 0:**	2 or 3 EDTA-tubes, buffy coat in cryovial	**80 XX XXX 1 buf coat**
*** T = 60:**	2 or 3 EDTA-tubes, buffy coat in cryovial	**80 XX XXX 2 buf coat**
*** T = 120:**	2 or 3 EDTA-tubes, buffy coat in cryovial	**80 XX XXX 3 buf coat**
*** T = 180:**	2 or 3 EDTA-tubes, buffy coat in cryovial	**80 XX XXX 4 buf coat**
*** T = 10wks:**	2 or 3 EDTA-tubes, buffy coat in cryovial	**80 XX XXX 5 buf coat**

Storage

- Store the plasma at < -70^{0} C
- Ship on dry ice (organized by UM)
- Avoid repeated freeze-thawing

Store the cryovials in the boxes the way as described in the SOP, storage blood samples!

Determination of cortisol, VLDL and IGF-I in serum

Recommendations for collection, processing and storage of specimen.

Collection

Tubes: 2x vacutainer (5ml, SST Gel and Clot Activator), Becton Dickinson ref.no. 368986.

- Draw at least 5 mL blood in each tube
- Store the tube at roomtemperature (at least during 30 minutes) until serum can be seperated

Processing

- Centrifuge the tube within 1 hr of collection for 15 minutes at 1200g (3650 RPM), 20 - 25° C.
- Transfer the serum to a clean, labeled cryovial.
  (Microtube with cap, 2ml. Sarstedt BV. ref.no. 72.694)

**T = 0:**	Put 0.5 ml serum in **7** cryovials with code (3 cryovials will have the real barcode)	**80 XX XXX 1 serum**
**T = 60:**	Put 0.5 ml serum in **6** cryovials with code (2 cryovials will have the real barcode)	**80 XX XXX 2 serum**
**T = 120:**	Put 0.5 ml serum in **6** cryovials with code (2 cryoials will have the real barcode)	**80 XX XXX 3 serum**
**T = 180:**	Put 0.5 ml serum in **6** cryovials with code (2 cryovials will have the real barcode)	**80 XX XXX 4 serum**
**T = 10 wks:**	Put 0.5 ml serum in **7** cryovials with code (3 cryovials will have the real barcode)	**80 XX XXX 5 serum**

Storage

- Store the serum at < -70° C
- Ship on dry ice (organized by UM)
- Avoid repeated freeze-thawing

Store the cryovials in the boxes the way as described in the SOP, storage blood samples!

Manual for the bloodsampling, storage and barcodelabels.

At the Clinical Investigation Day you have to start collecting bloodsamples. To make the use of the different SOP's more easy, this manual is produced.

The collection of the bloodsamples take place during 5 different timepoints. These are:
T = 0
T = 60
T = 120
T = 180
T = 10 wks

These timepoints are also on the barcodelabels. In that way we always will know at what timepoint the samples were taken.

Timepoint:	Barcodelabel:
T = 0	80XXXXX 1
T = 60	80XXXXX 2
T = 120	80XXXXX 3
T = 180	80XXXXX 4
T = 10wks	80XXXXX 5

This is according to the **SOP of bloodsampling**.
We will use EACH timepoint:

3 vacutainers of 4 ml (EDTA,lavendel top)
2 vacutainers of 5 ml (SERUM, yellow top)

After the collection and after the tubes are centrifuged the plasma and serum will be transfered to the cryovials (microtube with cap, 2 ml, Sarstedt)

EDTA

The 3 vacutainers of plasma (EDTA) will be divided over 9 cryovials.

You will have 9 times the barcodenumber 80XXXXX 1 for T = 0
EDTA

You will have 9 times the barcodenumber 80XXXXX 2 for T = 60 etc.
EDTA

That means you will have 9 cryovials each timepoint with exactly the same barcodenumber.

SERUM

The 2 vacutainers of SERUM will be divided over 7 cryovials at T = 0 and T = 10wks.

4 of them will have the barcodenumber, i.e. 80XXXXX1 and 80XXXXX 5
SERUM SERUM

3 of them will have the **REAL** barcodelabel, i.e.

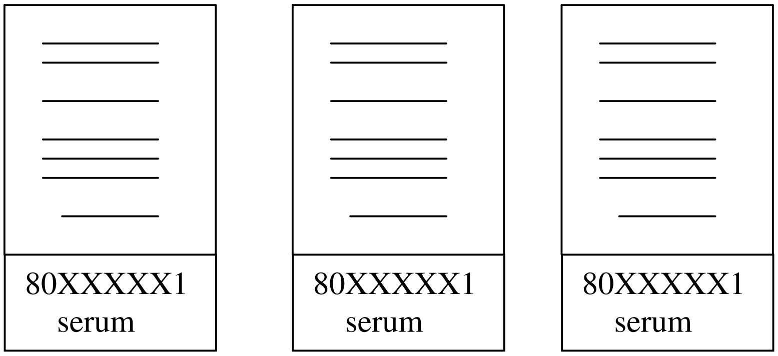

(or 80XXXXX5) (or 80XXXXX5) (or 80XXXXX5)

At timepoint 2, 3 and 4, you will have 6 cryovials.

4 of them will have the barcodenumber, ie.

80XXXXX 2 or 80XXXXX 3 or 80XXXXX 4
SERUM SERUM SERUM

2 of them will have the **REAL** barcodelabel.

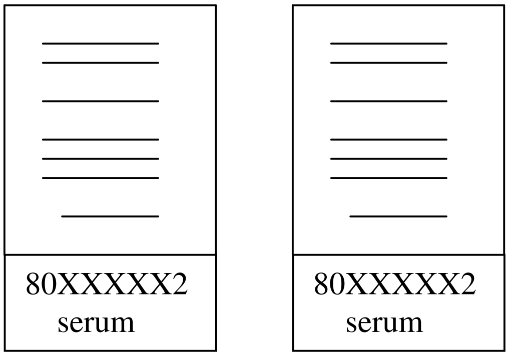

(or 80XXXXX3) (or 80XXXXX3)

(or 80XXXXX4) (or 80XXXXX4)

BUFFY COAT

After the transfer of the plasma from the EDTA-tube you can take the buffy coat.
You will have 3 tubes of EDTA
You take 1 cryovial with barcodenumber for each timepoint:

80XXXXX 1 for T = 0
Bufcoat

80XXXXX 2 for T = 60
Bufcoat etc.

At the Clinical Investigation Day you have had already four timepoints and all the samples from these timepoints are in the liquid nitrogen.

You will notice that some **REAL** barcodelabels and some barcodenumbers are left on the page of the booklet. (fatbiop not taken into account).

If you have done everything right there will be left:

(for timepoint 1 and 5)

2 **REAL** barcodelabels with the text CARD 1 and CARD 2
1 barcodenumber with the text EDTA
1 barcodenumber with the text SERUM
1 barcodenumber with the text BUFCOAT

(for timepoint 2, 3 and 4)

2 REAL barcodelabels with the text CARD 1 and CARD 2
1 REAL barcodelabel with the text SERUM
1 barcodenumber with the text EDTA
1 barcodenumber with the text SERUM
1 barcodenumber with the text BUFCOAT

These are spare in case you need one more label.

(NB. For the reference there are also labels for fatbiopsy and that is also true for the timepoints 2, 3 and 4. They don't have to be used !!! Timepoint 5 is only for a subgroup, if you decide on that. For the reference, there is also no timepoint 5.)

STORAGE

Then we have to go on with the storage of the samples.
Because of the different partners and subcontractors whom will analyse the samples, there will be seperate boxes for a certain amount of cryovials. It is important that you take care that enough and the correct cryovials go in to the right boxes.

For this I will also refer to the **SOP for storage.**

Boxes for Maastricht containing EDTA and SERUM samples

In a box for Maastricht you have to put 7 cryovials of EDTA of EVERY timepoint.
Start with seven samples of timepoint 1 (T=0), than seven samples of timepoint 2 (T=60), etc. Leave some space for timepoint 5 (T=10wks)
The next row in the box will be SERUM samples.
Put 2 cryovials of SERUM of every timepoint, also starting with two samples of timepoint 1, two samples of timepoint 2, etc.

The SERUM samples in this box have only the barcodenumber !!

Boxes for Steno, Kopenhagen containing BUFFY COAT.

Put the cryovials containing buffy coat into a seperate box. Starting with one sample of timepoint 1, one sample of timepoint 2, one sample of timepoint 3, **NO** timepoint 4,
and leave 1 space for one more sample at timepoint 5.
Then you will have 1-2-3-5-1-2-3-5-1-2-3 etc.

Boxes for subcontractor **STEINER 1**, Maastricht.
(The content of this box refers to barcodelabel CARD 1, see later and boxinlay)

In this box there will be the samples with the **REAL** barcodelabel.
For timepoint 1 and timepoint 5, there will be 2 samples which go in the box.
For timepoint 2, 3 and 4 there will go 1 cryovial in the box.
So you will have 1-1-2-3-4-5-5-1-1-2-3-4-5-5 etc.
Again, leave some space (2 spaces) for timepoint 5, if you should have samples of a second subject.

Boxes for subcontractor **STEINER 2**, Maastricht.
(The content of this box refers to barcodelabel CARD 2, see later and boxinlay).

In this box there will also be samples with the **REAL** barcodelabel.
For every timepoint there will be 1 cryovial in the box.
So you will have 1-2-3-4-5-1-2-3-4-5 etc.
Again, leave space for timepoint 5.

All the samples that are left are for your centre, you can store them the way you like.

BOXINLAYS

The boxes are filled but what to do with the boxinlays?

For a quick and easy way of searching the samples it is useful to know in which box what samples are.
Therefore we developed the boxinlays.

When a box is complete, you take the correct boxinlay (in duplo !) and write down what samples are in the box. You also, and that is important as well, give the box on the outside a number, doesn't matter what number as long as they are not the same (start with partnernumber, f.e. 5-01 and the next 5-02).
The same number is written down on the boxinlay.
You have to put ONE boxinlay in the box itself, the duplo is collected and sent together with the shipmentpapers (only if you sent that box of course).

And then there are the barcodelabels with the text CARD 1 and CARD 2.
When you finish the boxinlay for the box from Steiner (Steiner 1 and/or Steiner 2) you have to finish the cardform 1 and/or cardform 2.

For every **REAL** barcodelabel that is in the box goes the same **REAL** barcodelabel with the text CARD 1 on the cardform 1.
(Notice that you will have two samples of timepoint 1 and 5, and just one barcodelabel for timepoint 1 and one barcodelabel for timepoint 5. Just one barcodelabel on the cardform is enough for these timepoints (1 and 5)).
Put the boxinlay in the box of Steiner 1, the duplo AND the cardform 1 together will be sent together with the shipmentpapers.

The same goes for Steiner 2.
For every **REAL** barcodelabel that is in the box goes the same**REAL** barcodelabel with the text CARD 2 on the cardform 2.
(No double samples at a timepoint).
Put the boxinlay in the box of Steiner 2, the duplo AND the cardform 2 together will be sent together with the shipmentpapers.

I hope this manual will give a clear overview of the meaning of all the SOP's concerning thes handlings.
Always look at the original SOP's for the correct instructions. These final versions can be found on the internet www.nugenob.com at the membersite (WP4).
This is just a manual, and NO SOP.

Good luck !

Vacutainers for bloodsampling:

Becton Dickinson BV.

vacutainer 4ml, 7.2mg K2 EDTA **ref. no. 368861**

all partners (exc. 11) **2200** vacutainers total
partner 11 **1200** vacutainers total

vacutainer 5ml, SST Gel and Clot activator **ref. no. 368986**

all partners (exc. 11) **1500** vacutainers total
partner 11 **800** vacutainers total

Info on: www.bd.com

Becton Dickinson UK Limited
Vacutainer systems
Between towns road, Cowley, Oxford OX4 3LY
Tel. 01865 748844 Fax. 01865 781523

Cryotubes for plasma/serum/buffy coat.

Sarstedt BV.

microtube 2 ml with cap **ref.no. 72.694**

all partners 9exc. 11) **9000** tubes (18 sacs) total
partner 11 **5000** tubes (10 sacs) total

Info on : www.sarstedt.com

All adressess on the webpage available.

Boxes for storage.

Nalgene labware

SYSTEM 100 cryobox **ref. no. 5026-1010**

all partners (exc. 11) **121** boxes
partner 11 **63** boxes

Info on : www.nalgenunc.com

Standard Operation Procedure
Needle Subcutaneous Fat Biopsy

Peter Arner,
Britt-Marie Leijonhufvur

Huddinge University Hospital
Karolinska Instituttet
Sweden

Peter.Arner@medhs.ki.se

The procedure for taking the abdominal fat biopsy is described in the video made by Peter Arner, Britt-Marie Leijonhufvur. Pleas look the video over carefully and flow it regarding the procedure for **taking** the fat biopsy.

This written SOP is not supposed to be used instead of the video!!

Rather, it serves as summary and a documentation of the procedure.

1) Wash the area on the abdomen with surgical sprit
2) Anaesthetise the skin and the subcutaneous fat tissue with lidocain
3) Sterilise the area with coloured surgical sprit containing chlorine hexidine
4) Incision with a knife at the place of the injection of the anaesthetisation
5) Special needle for fat biopsy adapt it to the Hepafex syringe
6) Put the pistil of the syringe to the bottom and put in the needle in the anaesthetised area
7) Create a vacuum by backing the pistil as much as you can
8) Take a grip in the skin, so that you hold on the end of the needle, and move it slowly out-and-in, while rotating it at the same time
9) Go on for 1 minute or so, until no more tissue or blood is coming into the syringe, and you have lost your vacuum.

Regarding left vs. right position of fat biopsy:

Use both sides of abdominal wall for fat biopsy. Use one side as first biopsy and the other side for second biopsy after the diet period. Star with left or right side in a randomized fashion.

In case there is an additional biopsy immediately after the test meal during first examination use the same side as the first biopsy.

Instruction procedure for Ventilated Hood measurements. (REE).

Calibration:
Calibrate the systems before every measurement, according to the "Instruction manual".
This procedure requires gas mixture as prescribed in the manual. For Jaeger Oxycon a 5 vol.% CO_2/N_2 is used. The volume is calibrated by a 2-litre calibration syringe (Jaeger Oxycon).
Remember to save and print the calibration report.

Alcohol tests:
At least every fortnight a 30 min alcohol-burning test (10 grams of alcohol) is performed.
Before the test, the system is calibrated as described above.

PROCEDURE:
An alcohol burner filled with 99.9 % ethanol is weighed with an accuracy of ± 0.01 gram and then placed and burned under a special canopy.
The size of the flame or the flow is adjusted to obtain a CO_2-concentration between 0.6-1.0 %.
After the alcohol flame is turned on, the recording is started in the usual mode.
The test is running for about 30 minutes and after that the alcohol burner is weighed again.
As the first 5 min is used as an equilibration period only mean values ± SD of the VO_2, VCO_2, RQ and energy expenditure from minute 5-30 are calculated and stored.
From the combustion of alcohol the theoretic values for VO_2, VCO_2, RQ (0,667) and EE are calculated and then compared with the measured values.
An O_2- and CO_2 factor is calculated by which all later measurements are corrected (an example will follow).

Hood measurements:
The subject must rest comfortably for at least 15 min. before the first measurement is initiated.
Any tissue biopsy procedure should be terminated at least 30 minutes before an energy expenditure measurement is initiated.
PROCEDURE:
Calibrate the system before each period (see "Calibration").
Place the canopy over the subjects' head and connect the tube at the top of the canopy to the Blower unit.
Place the room air sampling tube in front of the canopy **never near the Blower unit.**
After zeroing and measurement of room air the recording is started.
Measure in intervals of 25 min. using the first 5 min. as an equilibration period.
If necessary use the equilibration period to adjust the flow rate in order to obtain a CO_2-concentration between 0.6 - 1.0 %.

Comments:
The subject is not allowed to: Listen to the radio, watch television, talk or sleep under the measurements.
If possible only the operator should remain in the room under the measurements.
The VAS scores are filled in during the calibration period
In the breaks between the measurements the subject is requested to be as relaxed as possible, but is allowed to sit up or go to the toilet.
Before any calibration, alcohol test or hood measurement the operator must insure the ventilation of the room.

Before initiating the NUGENOB study are all centres instructed to:

a) **Mail technical details (brand name, type and manufacturer)**
b) **Provide O2, CO2, RQ and EE results from**
    1) **The last ten 25 minutes alcohol tests**
    2) **On a group of at least 10 subjects measured twice for at least 25 minutes to st@kvl.dk**

RESULTS

Please save the following data on all subjects for each 20 minutes period during the whole 3½ hours measurement i.e. baseline + 6 post prandial measurements pr subject:

VO2 ± SD (ml/min)
VCO2 ± SD (ml/min)
RQ ± SD
EE ± SD (kcal/min) or (kcal/d)

There might be differences in the equipment equation for energy expenditure (EE) as some are using De V. Wier (1) others Elia & Livesey (2) or others. Based on the VO2 and VCO2 this can be transformed afterwards either locally or centrally. Therefore only VCO2 (ml/min) and VO2 (ml/min) are entered in the database.

1) J.B. de V. Wier J Physiol 109: 1-9, 1949
2) Elia M, Livesey G. Energy expenditure and fuel selection in biological systems: the theory and practice of calculations based on indirect calorimetry and tracer methods. In: Simopoulos AP (ed): Metabolic control of eating, energy expenditure and the bioenergetics of obesity. World Rev Nutr Diet. 70, pp 68-131, 1992. Karger, Basel.

SOP for liquid testmeal

Everybody (except Prague, Pamplona and Maastricht) has to use the cream of 40% fat/100g (So called double cream)

The composition of the test meal per 100 ml will be:

± 40 g fat	± 95 en% fat	
± 2-3 g carbohydrate	± 3 en% carbohydrate	The total energy content:
± 2-3 g protein	± 2 en% protein	375 kcal / 100 g

It is more palatable if you warm the cream a little bit. For the taste, add a few drops of vanille-arome and liquid sweetener (±6 drops / 100g test meal)
(6 drops = 1 thee spoon of sugar)

The energy content is fixed at 50% of the calculated 24 h BMR.
(See WHO-formulas on next page)

For Prague, Pamplona and Maastricht there is no such cream commercially available.

They have to use cream with 35% fat/100 g. **(Prague 33% fat).**
The fat percentage will be adjusted to 40% fat with butter.

The composition of the cream:
35 g fat
3 g carbohydrate
2 g protein

The composition of the butter:
82 g fat
1 g carbohydrate
0.5 g protein

The composition of the test meal is (per 100 g):	**For Prague**:
90 g cream + 10 g butter	85 g cream + 15 g butter

40 g fat	95 en% fat	
3 g carbohydrate	3 en% carbohydrate	Total energy content:
2 g protein	2 en% protein	378 kcal / 100 g

Melt the butter and add it to the cream.
Warm up the cream until the butter dissolves into the cream.
Add some vanille-arome and artificial sweetner (see before)

Serve when it is still warm. Subjects have to consume liquid test meal within 10 minutes.

Equations for the prediction of basal metabolic rate*

Equations for predicting BMR from weight (kg) and height (m)

	Age range (years)	BMR (kcal)
Men	18 - 30	15.4W - 27H + 717
	30 - 60	11.3W + 16H + 901
Women	18 - 30	13.3W + 334H + 35
	30 - 60	8.7W - 25H + 865

*WHO Technical report series 724, Geneva 1985

SOP "Flowchart CID Obese" NUGENOB

This SOP was developed for the NUGENOB project (www.nugenob.org).

Any use or quoting of this document should be acknowledged by a reference to www.nugenob.org

Birth date:_____ Subject initials:______ Date(dd/mm/yy):_______ Subject number:__________

Time*	***Time point***	***Note actual time***	***Action***
7.30			Anthropometry & Bioimpedance
8.00h	T ~ -90	__:__ **h**	Placement of catheter and subsequent
		__:__ **h**	Fat biopsy
8.30h	T ~ -60	__:__ **h**	Calibration of hood/ subject lying on bed with hood
9.00h	T ~ -30	__:__ **h**	Start hood measurement [RMR -30 - 0]
~9.20h		__:__ **h**	End hood measurement [RMR -30 - 0]
			VAS-scale**
		__:__ **h**	Blood sample
9.30h	T~ - 10	__:__ **h**	**TEST MEAL** Test meal ingested within **10** minutes,
	**T=0**	__:__ **h**	start thereafter T = 0
10.10h	T=30	__:__ **h**	End hood measurement [0 -30]
10.40h	T=60	__:__ **h**	End hood measurement [30-60]
			VAS-scale**
		__:__ **h**	Blood sample
11.10h	T=90	__:__ **h**	End hood measurement [60-90]
11.40h	T=120	__:__ **h**	End hood measurement [90-120]
			VAS-scale**
		__:__ **h**	Blood sample
12.10h	T=150	__:__ **h**	End hood measurement [120-150]
12.40h	T=180	__:__ **h**	End hood measurement [150-180]
			VAS-scale**
		__:__ **h**	Blood sample
12.50h	T=200	__:__ **h**	Option for second fat biopsy in subgroup

* Centres may have different meeting times. The starting time is an example

** Pleas note that actual time for VAS is currently n+A25ot reported in the CRF.
However, actual time should be noted on the VAS-score form.

Hood measurement of RMR

Pleas note that each hood measurement should last 20-25 minutes, allowing for a break of 5 minutes for VAS-scores, blood samples and resting of the subject.

Gabby Hull (partner 5)
G.Hul@hb.unimaas.nl
Camilla Verdic (partner 1)
cv@ipm.hosp.dk

SOP for the 2nd CID in obese subjects

Measures at the 2nd CID

After 10 weeks of dietary intervention following measures are performed on the fasting subjects:

- Fasting body weight (as in SOP_for_anthropometry.doc),
- Waist and hip circumference (as in SOP_for_anthropometry.doc),
- Body composition (as described in the manual from BodyStat),
- Abdominal subcutaneous fat biopsies (as in SOP for taking the abdominal fat biopsy).
- Fasting blood sampling (as in Manual_bloodsampling.doc and samplecodes.doc),

Pleas note that these measurements should take place at the same time in the morning as on 1st CID, and in the same order.

Remember that the subject should rest for one hour following the fat biopsy before blood samples are taken!!

Metabolic measures

No metabolic measures are required on the 2nd CID.

Timing of the 2nd CID

Ideally, this should be conducted in the 10th week after start of the dietary programme. It was questioned if CID in the 9th or the 11th week would be acceptable if this is more convenient for the subjects, for example due to work shifts. It was decided that the beginning of the 11th week or the end of the 9th week would be acceptable, but this should not be presented as an option to the subjects, but rather kept as an escape solution. The date of the second CID should be discussed with the subject prior to the start of the intervention, and the subject should be required to confirm their availability.

SOP for timeline for clinical investigation day NUGENOB
This SOP was developed for the NUGENOB project (www.nugenob.org)
Any use or quoting of this document should be acknowledged by a reference to www.nugenob.org

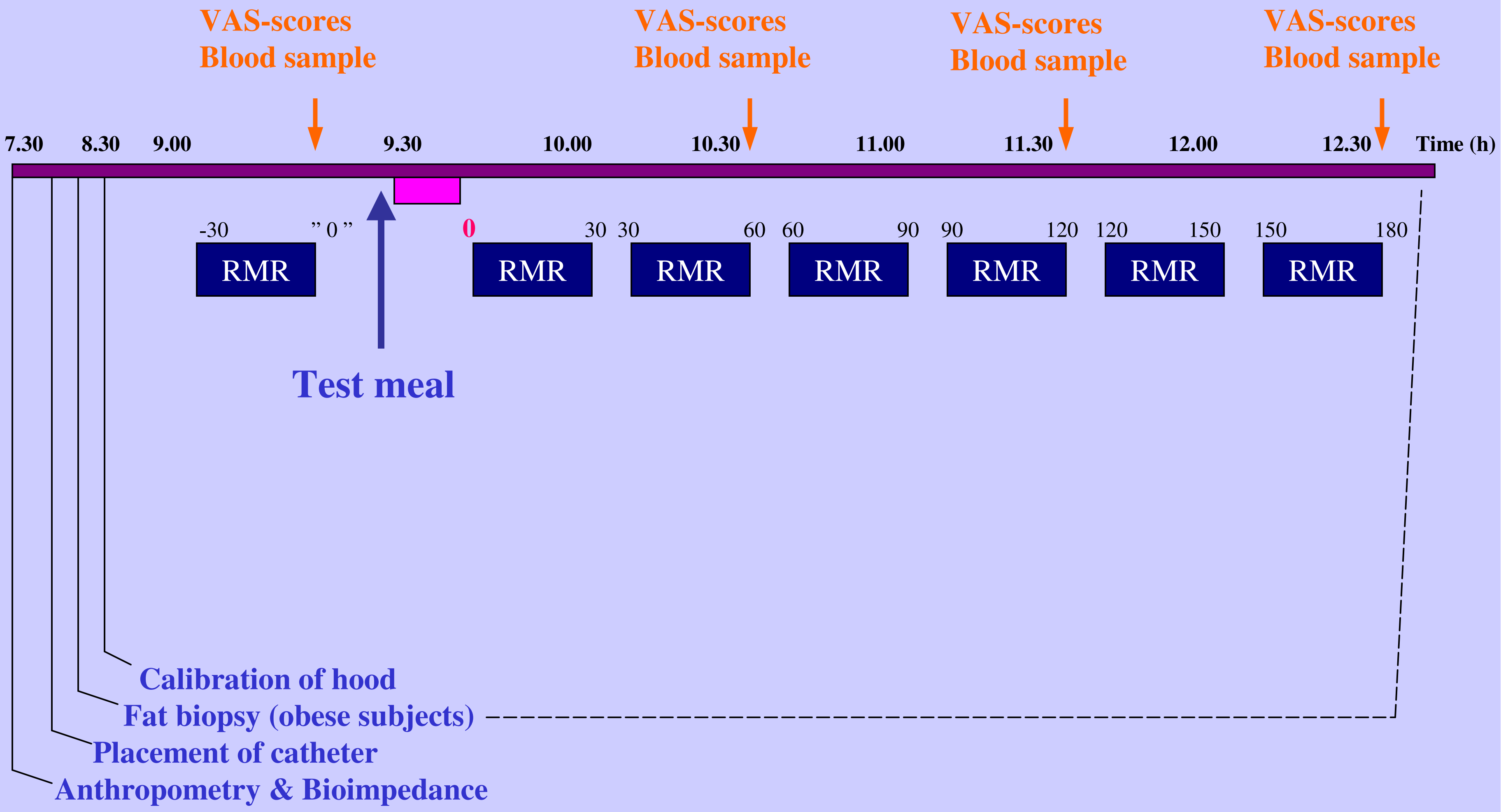

SOP for dropouts and counting of subjects:

Each clinical center has to include 100 (Toulouse 50) obese subject. Only the subjects that actually attend and complete the Clinical Investigation Day are to be counted. Thus, if a subject withdraws from the study at the end of the CID, this subject is to be included as one of the 100 obese. Using this procedure we assure that each center dose not have to perform more than 115 completed CID (100 on obese and 15 on lean subjects, respectively).

If the CID has been completed and the subject drop out at some point here after, the CRF is filled out as long as possible (i.e. minimum to page 14) and page 29 where there is room for filling out why the subject dropped out.

SOP for recording wellbeing and vomiting during the CID

All events of vomiting should be recorded as a remark in the CRF, stating the time of the events and the grade of severity (mild, moderate or severe).

During the CID the subjects should not be asked about their wellbeing, unless, of cause, you suspect that they are feeling very uncomfortable and need some form of help or rest.

At the end of the CID, the subjects may be asked to answer the following questionnaire. The se of the questionnaire is optional and was introduced in January 2002. These data cannot be entered into the CRF for the CID. Later on there will be a separate CRF for these data.

SE SUBJECT QUESTIONAIR ON THE FOLLOWING PAGE

Questionnaire
Wellbeing during the examination day in NUGENOB

Birth data: ________

Subject initials: _________

Date (dd/mm/yy): ___-___-02

Subject number: _____________

Note actual time of this recording: _____h

How would you describe your general well-being throughout the last 5 hours?

Mark one option

Really Good	
All right	
Neither good nor bad	
Not so good	
Really bad	

Do you have any specific symptoms or complaints?

	Yes	No
Nausea		
Stomach pain		
Heartburns		
Upset stomach		
Diarrhoea		
Dizziness		
Fell like fainting		
Palpitations		

Other comments:____
____
____

SOP for dietary assessment and intervention for public domain ©2001
Copyright © by the NUGENOB consortium
Neither this document nor any parts may be reproduced or transmitted in any form or by any means, electronic or mechanical, without permission in writing from the NUGENOB consortium.
Any use or quoting of this document requires notification of and accept from the NUGENOB consortium represented by Professor Thorkild IA Sorensen tias@ipm.hosp.dk

Nutrient-Gene Interactions in human obesity: Implications for Dietary Guidelines (NUGENOB)

Summary of dietary assessment and intervention SOPs for public area of NUGENOB website

Revised 06.07.01
Amended 18.07.01
Amended 03.12.01
Revised for public domain 19.08.04
Moira.Taylor@Nottingham.ac.uk

SOP for dietary assessment and intervention for public domain ©2001
Copyright © by the NUGENOB consortium
Neither this document nor any parts may be reproduced or transmitted in any form or by any means, electronic or mechanical, without permission in writing from the NUGENOB consortium.
Any use or quoting of this document requires notification of and accept from the NUGENOB consortium represented by Professor Thorkild IA Sorensen tias@ipm.hosp.dk

LIST OF CONTENTS AND APPENDICES

1. Dietary Intervention Targets
Primary Targets (NUGENOB protocol- 17.3.01) page 2
Additional Targets- discussed at Maastricht 17.3.01 page 2
Additional Targets – discussed in Pamplona 20.10.01 page 3

2. Guidance for Delivery of Intervention (NUGENOB protocol- 17.3.01) page 3

3. Required Measurements relating to Dietary Intake (revised at Vienna Meeting 2.6.01) page 3

4. Proposed Method of achieving Dietary Intervention targets page 3
4.1. Background
4.2. Methodology page 4
Template design
Aids to compliance
Use of the dietary template system with individual subjects page 5

APPENDIX I page 12
- SOP for weighed food record – training session, written instructions and blank record chart for obese and reference subjects

APPENDIX II page 21
- SOP for analysis of weighed dietary record

APPENDIX IIIa page 22
- Use of the template method – Template Design (UK)

APPENDIX IIIb page 23
- Template – Low fat intervention 2000kcals (UK)

APPENDIX IIIc page 26
- Template – High fat intervention 2000kcals (UK)

APPENDIX IV page 29
- Recipe suggestions for main meal (UK)

APPENDIX V page 31
- SOP for taking a diet history

APPENDIX VI page 36
- SOP for subject information leading up to the clinical investigation day

SOP for dietary assessment and intervention for public domain ©2001
Copyright © by the NUGENOB consortium
Neither this document nor any parts may be reproduced or transmitted in any form or by any means, electronic or mechanical, without permission in writing from the NUGENOB consortium.
Any use or quoting of this document requires notification of and accept from the NUGENOB consortium represented by Professor Thorkild IA Sorensen tias@ipm.hosp.dk

Dietary Intervention Targets

AIM:
To deliver a dietary intervention programme incorporating the individual dietary intake targets specified by the modified NUGENOB protocol

OBJECTIVES:
1. **To provide appropriate dietary information**
2. **To support and motivate subjects in following the diet**

1. Dietary Intervention Targets
Primary Targets

Ten week hypocaloric diet as either:
   a) Low fat/high carbohydrate diet
      20-25 en% fat, 15 en% protein, and 60-65 en% from carbohydrate;
   b) High fat/low carbohydrate diet
      40-45 en% fat, 15 en% protein, and 40-45 en% from carbohydrate.

- Daily energy intake to equal estimated daily energy requirement (measured basal metabolic rate multiplied by 1.3) minus 600kcal.
- Emphasis to be placed on consumption of common local food items which will be purchased and prepared by subjects.
- Where exclusion of alcohol is not possible, intake should be minimal (amount to be decided and reported at each clinical centre). Energy from alcohol should be subtracted from total energy intake then macronutrient intake calculated on the remaining energy (Se amendment from the Pamplona meeting below)

Additional Targets
- Where possible avoid those viscous soluble fibres that are thought to have the greatest impact on glucose and lipid metabolism (ie. oats and guar gum).
- Attempt to standardise other sources of soluble fibre in the two diets (eg. fruit and vegetables especially legumes).
- Encourage subjects to consume equal amounts of polyunsaturated, monounsaturated and saturated fats by ensuring incorporation of olive oil (or equivalent) and sunflower oil (or equivalent) into each day's choices (in addition to saturated fat predominately from meat and dairy products).
- Avoid using food products including specialist margarines which contain added plant sterols, omega-3 fatty acids or soya compounds, and soya based products.
- Encourage consumption of oily fish at least once a week within the fat restriction of the diet.
- Attempt to maintain comparable ratios of simple sugars to complex carbohydrates in the two dietary interventions.
- When exclusion of alcohol is not possible, intake should be minimal, with an upper limit of two glasses (2 x 150mls) total, which is allowed at the weekend. This limit will have to be set and accepted by the subject prior to starting the intervention. Energy from alcohol should be subtracted from total energy intake then macronutrient intake calculated on the remaining energy.

SOP for dietary assessment and intervention for public domain ©2001
Copyright © by the NUGENOB consortium
Neither this document nor any parts may be reproduced or transmitted in any form or by any means, electronic or mechanical, without permission in writing from the NUGENOB consortium.
Any use or quoting of this document requires notification of and accept from the NUGENOB consortium represented by Professor Thorkild IA Sorensen tias@ipm.hosp.dk

- Subjects who are already taking vitamin and mineral supplementation before commencing the project are required to continue taking the same dose throughout the investigation and this intake is to be included in the intake analysis. (It has been pointed out that RDA of certain vitamins was not reached for subjects on a low calorie diet between 1000-1400kcals/day). However, supplementation may affect both gene expression as well as metabolism, and should therefore not be commenced until after the 10-week intervention period.
- For constipation the recommended treatment should be to drink more plain water, less tea and coffee and to use magnesium or other similar over the counter drugs for intermittent use.

Priority will be given to achieving the dietary intervention targets. National healthy eating recommendations will be incorporated where possible. Subjects will, for example, be encouraged to meet national targets for fruit and vegetables consumption but soluble fibre intake must be similar on the two diets. Subjects will be encouraged also to follow national recommendations over meal frequency where practical.

2. Guidance on delivery of intervention from NUGENOB protocol

- Subjects will receive dietary instruction in the diet programme by a trained dietitian for at least 3 hours.
- Instructions will be reinforced and body weight ideally measured on a weekly basis until the end of the intervention.
- Subjects will receive a dietary guideline to aid compliance.
- Results from the analysis of the food record obtained during weeks 2, 5 and 7 should be included in the dietary teaching of the subjects (e.g. in the visit of week 3 and 6).

3. Food recording during the dietary intervention

- For reasons of feasibility, the food recording should be restricted to a 1-day record made on weekdays during weeks 2,5 and 7 following start of the dietary intervention. This is in addition to the 3-day record prior to the intervention and at week 10, which would include two weekdays and one weekend day.
- See Appendix I for SOP teaching subjects how to undertake weighed food records, subject instructions and blank record chart. See Appendix II for SOP for analysis of dietary record.

4. Proposed method of achieving dietary intervention targets

4.1 Background

A review of the previous literature of studies requiring fat intake modulation gives little detail of either method of intervention delivery or intakes achieved. In many cases the success of the intervention is judged by a clinically relevant index (eg serum lipids) and no information is given about actual dietary change. In many interventions, other requirements of this protocol were not addressed and studies were not undertaken in multiple countries.

In this study subjects must receive information allowing them to meet precise targets for dietary fat, carbohydrate and protein intake in order to ensure the appropriate exposure of the genes to the prescribed environmental conditions. In the high fat/low carbohydrate intervention, rather than the more usual instance of reducing the subject’s fat intake and increasing carbohydrate, it

SOP for dietary assessment and intervention for public domain ©2001
Copyright © by the NUGENOB consortium
Neither this document nor any parts may be reproduced or transmitted in any form or by any means, electronic or mechanical, without permission in writing from the NUGENOB consortium. Any use or quoting of this document requires notification of and accept from the NUGENOB consortium represented by Professor Thorkild IA Sorensen tias@ipm.hosp.dk

may be necessary to increase fat intake whilst reducing carbohydrate. This must be achieved whilst maintaining the same percentage of energy from protein in the two diets and achieving an energy deficit of 600 kcals/24h. Subjects are required to select their own diet from readily available local foods as opposed to provision of ready prepared meals from the research centre.

General dietary advice whilst compatible with current dietary practice to achieve lifelong change is not sufficiently quantified to achieve the goals of this study in the limited time period. Exchange systems had proved to be too imprecise and based on regulating only one macronutrient (eg American diabetic association exchanges). The exception to this was a well developed exchange system in Denmark which includes an extensive database of foods making it appropriate for use in that country alone.

It is thus proposed that each country designs a limited number of dietary 'templates' which reflect dietary intake in their region. The templates will indicate when alternative foods can be selected to increase variety. Each country must achieve the same dietary intervention targets but their templates will reflect cultural habits. Some tailoring to individual need will be possible and demonstrated to the subject. It is intended however that the standard dietary templates be used with the majority of subjects so that preparation work can be undertaken by the dietitian prior to recruitment of subjects when the workload will be high.

Subjects with a chaotic eating pattern or excluding major food groups (eg vegetarian) will be urged to modify their eating behaviour in line with the template system in order to achieve the dietary targets. Subjects who indicate unwillingness to comply will be excluded.

4.2 Methodology

4.2a Template design

1. Design a menu for a 24-hour period that reflects cultural habits.
2. Identify the sources of fat in that day (you may find it helpful to use a 5g fat unit).
3. Modify the fat intake in line with the high and low fat diet requirements for the study.
4. Modify the menu so that the other study requirements are met whilst ensuring that you continue to achieve the fat targets.
5. Follow national healthy eating recommendations for foods and meal pattern where practical. Remember that meeting the dietary intervention targets and compliance is your priority.
6. Identify components of the menu that could be varied using 'alternative food lists'. Develop food lists; you might for example give a list of portions of bread, pasta and rice if you have used potato in your 24-hour menu. Remember that you must also maintain the other dietary intervention targets.
7. Repeat this process to take into account different energy requirements and eating patterns (eg. weekend/weekday).

These 'menus' are now your dietary templates.

Please see Appendix IIIa, b, & c for a worked example of this process from the United Kingdom.

4.2b Aids to Compliance

The following are suggestions for improving compliance:

1. Design an advice sheet for eating out.

SOP for dietary assessment and intervention for public domain ©2001
Copyright © by the NUGENOB consortium
Neither this document nor any parts may be reproduced or transmitted in any form or by any means, electronic or mechanical, without permission in writing from the NUGENOB consortium. Any use or quoting of this document requires notification of and accept from the NUGENOB consortium represented by Professor Thorkild IA Sorensen tias@ipm.hosp.dk

2. Create lists of ready prepared foods that could be incorporated into the menu.
3. Design recipes based on the basic food items included in the template and food lists (see Appendix IV for examples from the United Kingdom).
4. Design other information sheets that will help subjects comply in your area (eg. material based on how to cope with local festivals).
5. Develop resources based around cognitive behavioural therapy.

SOP for dietary assessment and intervention for public domain ©2001
Copyright © by the NUGENOB consortium
Neither this document nor any parts may be reproduced or transmitted in any form or by any means, electronic or mechanical, without permission in writing from the NUGENOB consortium. Any use or quoting of this document requires notification of and accept from the NUGENOB consortium represented by Professor Thorkild IA Sorensen tias@ipm.hosp.dk

4.2c Use of the dietary template system with individual subjects

1. Assess the subject's 3-day food record and select the most suitable template. It is intended that the template will be used with little modification once adjusted to achieve appropriate energy intake.

2. At first dietetic intervention session:

- Explain that the programme has been designed to meet the individual's needs based on their 3-day food record and energy expenditure measurement. Emphasise the importance of following instructions precisely so that they will lose weight **and** because they are involved in **an experimental protocol.**
- Show the individualised dietary template and explain how 'alternative lists' are used.
- Obtain a brief dietary history, if the clinical centre decides this is useful, (See Appendix V – SOP for taking a diet history) to obtain a broader view of dietary intake and encourage the subject to discuss how they will modify their intake in order to adhere to the programme.
- Use information from the dietary history, if obtained, to pre-empt potential problems with compliance, and select appropriate additional support material (eg. information about eating out if appropriate, modification of prepared meals).

3. Subsequent visits:

- Reinforce dietary message using additional support material
- Assess compliance – you may wish to use additional diaries
- Develop cognitive behavioural therapy
- Use telephone follow up for poor compliers

4. Non attendance:

- It has now been decided that illness during the dietary intervention period which might lead to a lower dietary intake should not lead to exclusion. Instead it should be carefully reported in the 'comments box' of the Case Report Form.

It is recommended that where subjects weigh themselves at home, this self reported weight should be recorded in the Case Report Form together with a comment in the 'comment box'.

SOP for dietary assessment and intervention for public domain ©2001
Copyright © by the NUGENOB consortium
Neither this document nor any parts may be reproduced or transmitted in any form or by any means, electronic or mechanical, without permission in writing from the NUGENOB consortium. Any use or quoting of this document requires notification of and accept from the NUGENOB consortium represented by Professor Thorkild IA Sorensen tias@ipm.hosp.dk

5. Summary Flow Diagram of Dietetic Involvement

Recruitment contact (phone or letter)

↓

First dietetic appointment

- Teach techniques for 3-day weighed food record/give food scales. See Appendix I – SOP for weighed food record
- Administer 'background information' questionnaire
- **Instruction in "standard behaviour" 3 days prior to clinical investigation day.** See Appendix VI – SOP for information leading up to the Clinical Investigation Day

↓

Subject completes 3-day weighed food diary

↓

Clinical Investigation Day

- Subject returns 3- day food diary

↓

Randomisation to Low/High fat interventions

↓

Pre- visit preparation by dietitian

- Calculate energy requirement based on 1.3 x measured BMR
- Assess and analyse 3-day food diary.
  See Appendix II – SOP for analysis of food record.
- Select appropriate template
- Make individual adjustments

↓

First dietetic intervention (as soon as practical after the clinical investigation day and not more than 14 days later)

- If first dietetic appointment takes place 7 days or more after the clinical investigation day then weight and bio-electrical impedence must be repeated.
- Introduce subject to the dietary intervention targets, their individualized template and use of alternative food lists
- Undertake dietary history (to be decided at each clinical centre) to encourage subject to think how they will modify their intake and identify potential problems with compliance.
  See Appendix V – SOP for taking a diet history.
- Introduce appropriate support material

↓

Each weekly visit (may be replaced by telephone contact in some weeks)

- Weigh subject if they are attending the centre
- Reinforce dietary message
- Introduce other therapeutic methods (eg. cognitive behavioural therapy)
- Assess weekly compliance with meal pattern, and content of the diet you may wish subjects to keep additional diaries using household measures
- Introduce additional support material as appropriate.
- Those found to be poor compliers may be offered telephone contact between weekly appointments

↓

SOP for dietary assessment and intervention for public domain ©2001
Copyright © by the NUGENOB consortium
Neither this document nor any parts may be reproduced or transmitted in any form or by any means, electronic or mechanical, without permission in writing from the NUGENOB consortium. Any use or quoting of this document requires notification of and accept from the NUGENOB consortium represented by Professor Thorkild IA Sorensen tias@ipm.hosp.dk

During weeks 2, 5, 7 and 10,

- assess compliance
- subject completes 1-day weighed food diary during weeks 2,5 and 7
- subject completes 3-day weighed food diary during week 10
- Analyse as soon as possible. See Appendix II – SOP for analysis
- Transfer required analyses to Excel & Send results to central database

SOP for dietary assessment and intervention for public domain ©2001
Copyright © by the NUGENOB consortium
Neither this document nor any parts may be reproduced or transmitted in any form or by any means, electronic or mechanical, without permission in writing from the NUGENOB consortium.
Any use or quoting of this document requires notification of and accept from the NUGENOB consortium represented by Professor Thorkild IA Sorensen tias@ipm.hosp.dk

APPENDIX I

SOP FOR WEIGHED FOOD RECORD – TRAINING SESSION, WRITTEN INSTRUCTIONS AND BLANK RECORD CHART FOR OBESE AND REFERENCE SUBJECTS

Rationale

A 3-day weighed food record completed prior to the Clinical Investigation Day will be used to obtain baseline data on intake, characterise normal dietary pattern and form the basis for selection of a dietary template for the intervention. Bingham (1987) outlines the complex nature of attempting to measure food intake. Individual centres may decide to include a validated food frequency questionnaire such as EPIC in addition to the weighed food record providing this does not compromise the quality of core data collected.
At the first dietary intervention appointment together with taking a dietary history (optional), the 3-day record will be used as the basis for discussion on typical intake and range of foods eaten. At three points during the intervention, weighed records will be used to assess compliance with the dietary regime; two, one day weighed records during weeks 2 and 5 and a second 3-day weighed record during week 10.

Instruction

It is intended that subjects will have written instructions on the use of dietary scales and the method of recording intake (attached). A practical session will also be arranged to ensure that subjects are competent with weighing and recording food and drink items (see ‘practical teaching session’ below).

Practical Teaching Session for Weighed Food Record

At the first dietetic appointment, the subjects will be given dietary scales. The dietitian will demonstrate how to weigh and record various items of food and drink. The subjects will then be asked to pick approximately three items (food models could be used) and carry out the weighing and recording procedure themselves. The subjects will be encouraged to ask any questions necessary for them to feel confident that they can weigh and record all the usual food and drink items that they would consume.

When subjects have performed the weighing and recording procedures satisfactorily, written instructions would be given together with a recording book and contact number for further queries. If the dietary scales used do not have a tare, the SOP will have to be modified.

After completion of Food Record

When the dietitian receives a completed food record, it should be reviewed within 3 days to highlight any possible errors or lack of adequate information for analysis. Where omissions have occurred, the subject will usually be contacted by telephone to clarify. The diary will then be entered and analysed on computer using a specified dietary analysis package within one week from receipt (See APPENDIX II; SOP for Analysis). The required output would be transferred to Excel then forwarded to the central collection point.

SOP for dietary assessment and intervention for public domain ©2001
Copyright © by the NUGENOB consortium
Neither this document nor any parts may be reproduced or transmitted in any form or by any means, electronic or mechanical, without permission in writing from the NUGENOB consortium.
Any use or quoting of this document requires notification of and accept from the NUGENOB consortium represented by Professor Thorkild IA Sorensen tias@ipm.hosp.dk

HOW TO WEIGH YOUR FOOD AND DRINK

Thank you for helping us. It is important that we know exactly what you have eaten and drunk. Please weigh and record *everything* you eat and drink (including water). Write down what you have to eat or drink in your food diary, giving as much detail as you can about each item. For instance, whether bread is white, brown, or wholemeal; whether fruit is eaten peeled or unpeeled; whether meat is fatty or lean, roasted, stewed, or grilled. Give the number of cups of tea or coffee, slices of bread, rashers of bacon, eggs, biscuits, and sweets (some example descriptions are given on the attached sheet).
Recipes for any special dish are very helpful, and can be written on the 'recipe' pages at the back of your food diary.

Remember to write down

- Everything you eat and drink at home;
- Anything you eat away from home; cups of tea (say whether you had milk and/or sugar);
- Between-meal snacks or nibbles whilst cooking;
- Beer, sherry, or other alcoholic drinks;
- Sweets and chocolates;
- Fruit;
- Crisps and nuts.

To weigh your food

- If you are going to eat a hot meal, warm a suitable plate while you are cooking your food;
- Switch the scales 'ON' and ensure that you are weighing in grammes 'g '.
- When using a 'dinner plate' it will be necessary to place a bowl (or similar) onto the scales first then put the plate on top of this so as not to obscure the display panel.
- Record the time of day in the 'Time eaten' column and where you are eating in the 'Where' column eg friends house, restaurant, kitchen.
- Place your plate or cup on the scales, recording the weight in the 'Wt served' column; Zero the scales; Do not remove the plate before adding a food item.
- Place one item of food on the plate or fluid in the cup, record the weight in the 'Wt served' column, and fill in the 'Food' columns with a full description of the item including the brand name if appropriate; Zero the scales;
- Add the next item of food and record its weight and description; Zero the scales. Repeat until you have served out your meal. Ensure that you do not remove the plate or cup between adding foods eg do not remove bread or toast in order to spread with margarine; spread margarine while bread or toast is still on the scales or alternatively add a small amount or margarine to the plate for spreading later.

Please record each item of food on a separate line.
If you have second helpings or any 'scrapings' from a dish, remember to include these in the same way as above.
After everything on your plate is written down, leave a line blank before your next plate.

SOP for dietary assessment and intervention for public domain ©2001
Copyright © by the NUGENOB consortium
Neither this document nor any parts may be reproduced or transmitted in any form or by any means, electronic or mechanical, without permission in writing from the NUGENOB consortium.
Any use or quoting of this document requires notification of and accept from the NUGENOB consortium represented by Professor Thorkild IA Sorensen tias@ipm.hosp.dk

If you have not eaten all the food on your plate, or if there is waste, like bones:

- Put another plate on the scales and record its weight; Zero the scales;
- Place one item of waste or left food on the plate and record the weight in the 'left-overs' column next to the food already detailed. If left food needs a separate description such as bones, write this in the 'Food' column on a new line and write the weight in the left- over column of that line; Zero the scales;
- Add the next item and record the weight; Zero the scales. Repeat until you have weighed out all the food and waste left.

Start a new page for each day and use as many pages as you need.
Enter the date and day of the week on every page.
Please do not fill in any of the other spaces.

If you have any queries, please ring ……………………………………………………

SOP for dietary assessment and intervention for public domain ©2001
Copyright © by the NUGENOB consortium
Neither this document nor any parts may be reproduced or transmitted in any form or by any means, electronic or mechanical, without permission in writing from the NUGENOB consortium.
Any use or quoting of this document requires notification of and accept from the NUGENOB consortium represented by Professor Thorkild IA Sorensen tias@ipm.hosp.dk

Recipes

We have included 'Recipe' pages at the back of your food diary. Use these to write down the details of any dishes made at home from several ingredients. Write down the name of the dish so that it can be referred to in the main part of your diary, then record details of the ingredients and cooking method on the 'Recipe' pages. Weigh all the ingredients and write down their weights. It may be useful to use a small bowl to weigh ingredients into before adding them to the dish. If you do this, ensure that the display reads 'zero' before adding your next ingredient.

After cooking, the completed dish will need to be weighed again. You may find that the original cooking vessel eg pan/casserole dish together with all the cooked ingredients is too heavy for the scales. If this is the case then the display would show 'Err'. In order to obtain the cooked weight of the dish you could weigh an empty plastic container, set the scales to 'zero' then add the cooked dish and record its weight.

Write down the weight of any leftovers. If you make a dish on one day then add something to it for another meal, include this on another recipe page, referring back to the original dish being added to.

Food Labels

Keep food labels or wrapping from any manufactured foods that you eat so that we can get accurate information about the product.

Food eaten away from home

Always take your food diary with you to record food eaten away from home. Also try to take your scales with you to weigh food but if you are unable do this, it will be necessary to estimate the food portion size in household measures (see list attached) and to keep any labels/wrapping to give more information about the food. It may also be useful to record the price of some items such as crisps or chocolate bars as this will give an indication of their size.

Items eaten or taken from the container

This would include items such as yogurts and other desserts. The full pot should be weighed without its lid and then the container weighed afterwards.

Spilt or dropped food

Try to recover any spilt or dropped food in order to weigh it. If this is not possible, estimate how much was lost in household measures and record in the leftovers column.

SOP for dietary assessment and intervention for public domain ©2001
Copyright © by the NUGENOB consortium
Neither this document nor any parts may be reproduced or transmitted in any form or by any means, electronic or mechanical, without permission in writing from the NUGENOB consortium.
Any use or quoting of this document requires notification of and accept from the NUGENOB consortium represented by Professor Thorkild IA Sorensen tias@ipm.hosp.dk

How to Describe your Food and Drink including Household Measures

We want you to weigh all your food. Should you however on rare occasions find that you cannot, please use the following ways of describing your food. Below some suggestions are given on how to describe certain food and drink items together with their household measures.

FOOD	DESCRIPTION OF FOOD OR DRINK AND BRAND	HOUSEHOLD MEASURE
Bacon	Lean or streaky; fried or grilled rashers, smoked or unsmoked	Number
Bread	Type of bread, eg. white, brown, wholemeal, granary, French stick, ciabatta, currant.<br>Description of slice eg. thin, medium or thick	Number of slices
Canned drinks	Type, brand name<br>For example: 330ml can Diet Coca Cola	Number or full or half can
Crisps	Type, brand name	Packet weight
Fruit	Type and size of fruit eg large granny smith apple<br>For tinned fruit; slices/ halves etc in juice or syrup	Number of pieces or tablespoons
Jams	Type, brand name	Teaspoons, heaped or flat
Milk	Type; full cream, semi skimmed, skimmed	Pints, glasses or cups
Oil	Type eg sunflower oil, corn oil, olive oil<br>Brand name	Tablespoons
Prepacked foods eg beefburgers, pies, biscuits, confectionery	Full name of product including brand name.<br>For example: Bird's Eye cod fish fingers, 10 for 99p.<br>Keep the package.	Number
Sandwiches	Describe fully if homemade (these should be weighed as they are being made) or if bought;<br>Full name, place of purchase and price, describe bread as above and note loaf size.	Number of slices of bread or number of rolls
Spreads on bread or toast	Type eg butter, low fat spread etc<br>Full description, % fat and brand name<br>Keep the package	Number of teaspoons or thinly, average or thickly spread
Sugar	Type	Teaspoons, heaped or flat
Sweets, chocolate and snack bars	Name, size (weight) and price (if known)<br>For example: king size Mars bar 46p<br>Keep the wrapper	Weight of bar or number of sweets
Takeaways	Describe in full, give name of restaurant and price	Portion size and

SOP for dietary assessment and intervention for public domain ©2001
Copyright © by the NUGENOB consortium
Neither this document nor any parts may be reproduced or transmitted in any form or by any means, electronic or mechanical, without permission in writing from the NUGENOB consortium.
Any use or quoting of this document requires notification of and accept from the NUGENOB consortium represented by Professor Thorkild IA Sorensen tias@ipm.hosp.dk

	For example: Large portion chips, The High Street chip shop – 75p	price
Vegetables	Type; fresh, frozen, tinned or dried<br>Brand name	Tablespoons, full or heaped

Copyright © by the NUGENOB consortium
Neither this document nor any parts may be reproduced or transmitted in any form or by any means, electronic or mechanical, without permission in writing from the NUGENOB consortium.
Any use or quoting of this document requires notification of and accept from the NUGENOB consortium represented by Professor Thorkild IA Sorensen tias@ipm.hosp.dk

RECIPE INFORMATION

Write down the details of any dishes made at home from several ingredients. Weigh all the ingredients and write down their weights.

Name of home-made dish: ……………………………………………………………….

When was the dish eaten? Time: …………… Day: …………… Date: ……………

Are you adding ingredients to a dish cooked before? Yes ☐ No ☐ If yes, name of dish: …………………………………...

Weight of container before adding food eg pan/bowl/plastic container ……………
Weight of container and the cooked dish ……………
Weight of container and any leftovers ……………

INGREDIENTS	Weight	OFFICE	OFFICE	INGREDIENTS	Weight	OFFICE	OFFICE

Copyright © by the NUGENOB consortium
Neither this document nor any parts may be reproduced or transmitted in any form or by any means, electronic or mechanical, without permission in writing from the NUGENOB consortium.
Any use or quoting of this document requires notification of and accept from the NUGENOB consortium represented by Professor Thorkild IA Sorensen tias@ipm.hosp.dk

Cooking Method in brief:

Day ………………………………………..			Date ……………………			Code No. (Office)	
Please us a separate line for each item eaten; write in weight of plate; leave a line between different ‘plate’ entries							
		**FOOD COLUMNS**					
Time eaten am/pm	Where	Brand name of each item, in full, (except for fresh produce)	Full description of each item including:<br>- whether fresh, frozen, dried, canned<br>- how cooked; what type of fat food fried in	Weight served (g)	Weight of leftovers	Office	Office

SOP for dietary assessment and intervention for public domain ©2001
Copyright © by the NUGENOB consortium
Neither this document nor any parts may be reproduced or transmitted in any form or by any means, electronic or mechanical, without permission in writing from the NUGENOB consortium.
Any use or quoting of this document requires notification of and accept from the NUGENOB consortium represented by Professor Thorkild IA Sorensen tias@ipm.hosp.dk

Modified from food record used in "The Dietary and Nutritional Survey of British Adults" (ref. No. 5)

SOP for dietary assessment and intervention for public domain ©2001
Copyright © by the NUGENOB consortium
Neither this document nor any parts may be reproduced or transmitted in any form or by any means, electronic or mechanical, without permission in writing from the NUGENOB consortium.
Any use or quoting of this document requires notification of and accept from the NUGENOB consortium represented by Professor Thorkild IA Sorensen tias@ipm.hosp.dk

References

1. Bingham S A, The dietary assessment of individuals; methods, accuracy, new techniques and recommendations. Nutrition Abstracts and Reviews (Series A). 1987, Vol. 57, No. 10.
2. Manual on Methodology for Food Consumption Studies edited by Cameron M E and Van Staveren W A. Oxford University Press 1988.
3. Human Nutrition and Dietetics 10th edition edited by Garrow J S, James W P T, Ralph A. Churchill Livingstone 2000.
4. Anderson A S, An overview of diet survey methodology. British Food Journal. 1995 Vol. 97, No. 7 pgs 22-26.
5. The Dietary and Nutritional Survey of British Adults edited by Gregory J, Foster K, Tyler H, Wiseman M. HMSO 1990.
6. Thanks to Dr M Nelson and Bridget Holmes, King's College, London and Dr S A Bingham, Dunn Nutrition Unit, Cambridge for personal communications.

SOP for dietary assessment and intervention for public domain ©2001
Copyright © by the NUGENOB consortium
Neither this document nor any parts may be reproduced or transmitted in any form or by any means, electronic or mechanical, without permission in writing from the NUGENOB consortium.
Any use or quoting of this document requires notification of and accept from the NUGENOB consortium represented by Professor Thorkild IA Sorensen tias@ipm.hosp.dk

APPENDIX II

SOP FOR ANALYSIS OF WEIGHED DIETARY RECORD

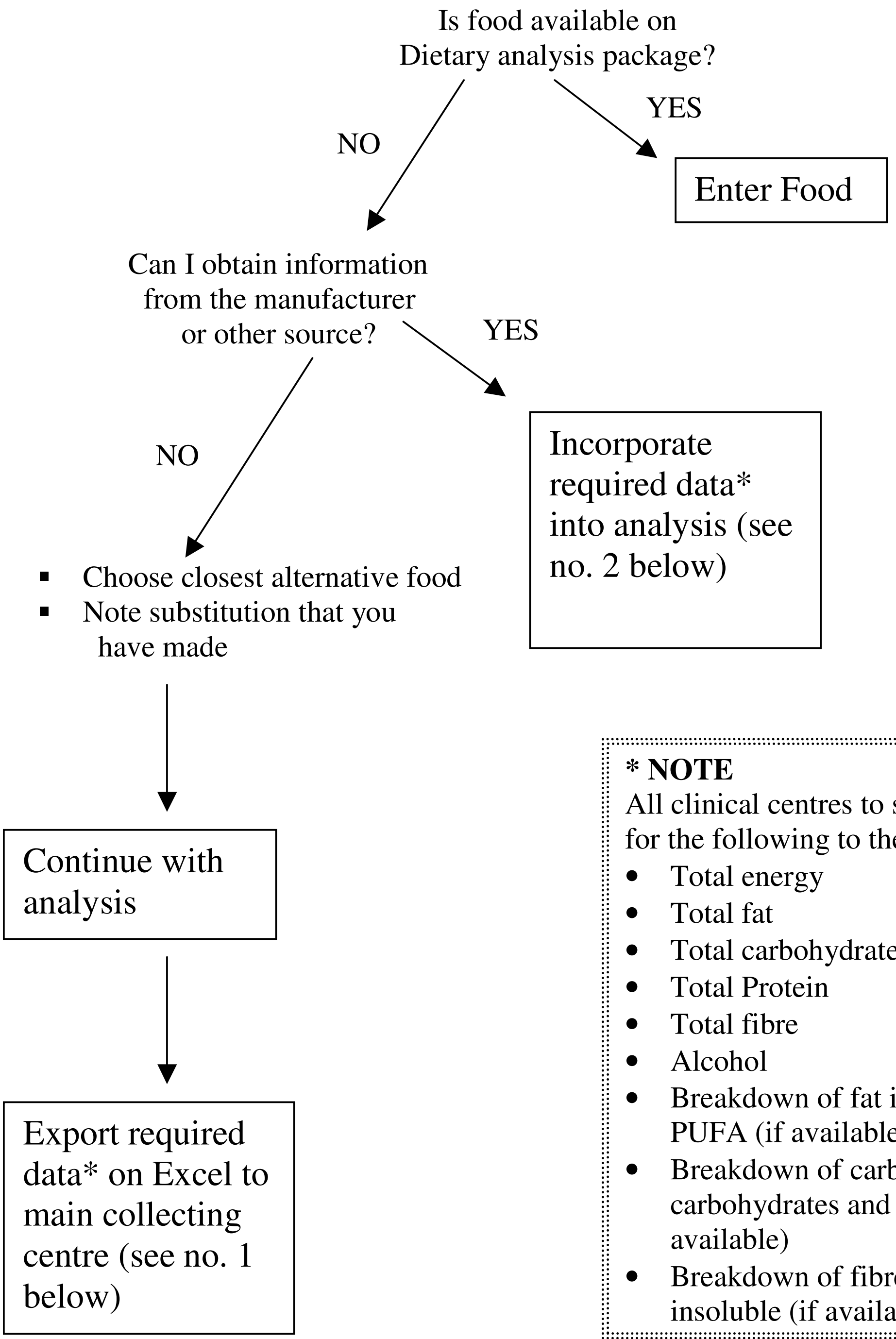

*** NOTE**
All clinical centres to submit dietary analyses for the following to the main centre:

- Total energy
- Total fat
- Total carbohydrate
- Total Protein
- Total fibre
- Alcohol
- Breakdown of fat into SFA, MUFA and PUFA (if available)
- Breakdown of carbohydrate into complex carbohydrates and simple sugars (if available)
- Breakdown of fibre into soluble and insoluble (if available)

SOP for dietary assessment and intervention for public domain ©2001
Copyright © by the NUGENOB consortium
Neither this document nor any parts may be reproduced or transmitted in any form or by any means, electronic or mechanical, without permission in writing from the NUGENOB consortium.
Any use or quoting of this document requires notification of and accept from the NUGENOB consortium represented by Professor Thorkild IA Sorensen tias@ipm.hosp.dk

1. Please keep the total analysis output for each subject as this may be required in the future.
2. Please try to obtain other micronutrients if you have to contact manufacturers for information.

SOP for dietary assessment and intervention for public domain ©2001
Copyright © by the NUGENOB consortium
Neither this document nor any parts may be reproduced or transmitted in any form or by any means, electronic or mechanical, without permission in writing from the NUGENOB consortium.
Any use or quoting of this document requires notification of and accept from the NUGENOB consortium represented by Professor Thorkild IA Sorensen tias@ipm.hosp.dk

APPENDIX IIIa – USE OF THE TEMPLATE METHOD

The following is a worked example of how the United Kingdom group has produced high fat and low fat templates.

TEMPLATE DESIGN

24-Hour Menu used to Develop Templates (Sample for the United Kingdom)

MACRONUTRIENT CONTRIBUTION
17% Protein, 52% Carbohydrate, 31% Fat

	Approximate Fat Content (g)
BREAKFAST	
3 Weetabix	2
Medium glass of unsweetened orange juice	
¼ pt semi skimmed milk	2
Cup of tea	
MID MORNING	
Cup of tea	
1 medium banana	
LUNCH	
Sandwich:	
2 slices of granary bread	2
with low fat spread	8
45g lean chicken	1
cucumber and tomato	
1 bag of potato crisps	11
1 apple	
MID AFTERNOON	
Cup of tea	
Medium slice of madeira cake	8
DINNER	
Stir fry with 90g lean diced pork	6
15g vegetable oil	15
mushrooms, onions and	
150g boiled rice	

SOP for dietary assessment and intervention for public domain ©2001
Copyright © by the NUGENOB consortium
Neither this document nor any parts may be reproduced or transmitted in any form or by any means, electronic or mechanical, without permission in writing from the NUGENOB consortium.
Any use or quoting of this document requires notification of and accept from the NUGENOB consortium represented by Professor Thorkild IA Sorensen tias@ipm.hosp.dk

SUPPER

Cup of tea

ADDITIONAL

½ pt semi skimmed

SOP for dietary assessment and intervention for public domain ©2001
Copyright © by the NUGENOB consortium
Neither this document nor any parts may be reproduced or transmitted in any form or by any means, electronic or mechanical, without permission in writing from the NUGENOB consortium.
Any use or quoting of this document requires notification of and accept from the NUGENOB consortium represented by Professor Thorkild IA Sorensen tias@ipm.hosp.dk

APPENDIX IIIb

TEMPLATE
LOW FAT INTERVENTION – 2000 KCALS
MACRONUTRIENT CONTRIBUTION
15% Protein, 62% Carbohydrate, 23% Fat

	**ALTERNATIVES**	Approximate Fat Content (g)
**BREAKFAST**		
3 Weetabix OR<br>&	60g Branflakes<br>Fruit'n Fibre (no oats) or<br>Shredded Wheat	2
150g orange juice OR	150g grapefruit juice or<br>one medium orange or<br>one medium apple or<br>90g grapes or<br>100g pineapple in juice or<br>100g mandarin oranges in syrup	0
**MID-MORNING**		
Snack from list (see snack lists A & B)		
**LUNCH**		
Granary bread,<br>3 medium slices OR<br>&	3 medium slices of white, brown,<br>wholemeal or Hovis bread or<br>100g french stick or<br>100g pitta bread (one large)	3
20g low fat spread OR<br>&	25g reduced calorie mayonnaise or<br>25g salad cream	8
45g lean chicken OR<br><br>&	45g lean beef, pork or ham or<br>1 egg boiled or<br>60g tinned salmon or<br>60g tuna tinned in brine with<br>10g reduced calorie mayonnaise or salad cream	1
Salad containing any of the following:<br>&	lettuce, cucumber, tomatoes, pickled beetroot,<br>cabbage, carrots, celery, pickled gherkins,<br>mushrooms, mustard and cress, peppers,<br>radish, spring onion, watercress	0
One medium apple OR	one medium orange or 90g grapes or	[illegible]
		0

SOP for dietary assessment and intervention for public domain ©2001
Copyright © by the NUGENOB consortium
Neither this document nor any parts may be reproduced or transmitted in any form or by any means, electronic or mechanical, without permission in writing from the NUGENOB consortium.
Any use or quoting of this document requires notification of and accept from the NUGENOB consortium represented by Professor Thorkild IA Sorensen tias@ipm.hosp.dk

100g pineapple in juice or
100g mandarin oranges in syrup or
150g grapefruit or orange juice or
2 kiwi fruit, clementines or fresh apricots or 200g
blackberries, gooseberries or raspberries or 320g tomato juice

SOP for dietary assessment and intervention for public domain ©2001
Copyright © by the NUGENOB consortium
Neither this document nor any parts may be reproduced or transmitted in any form or by any means, electronic or mechanical, without permission in writing from the NUGENOB consortium.
Any use or quoting of this document requires notification of and accept from the NUGENOB consortium represented by Professor Thorkild IA Sorensen tias@ipm.hosp.dk

Low fat intervention template continued

ALTERNATIVES

MID-AFTERNOON

		Approximate Fat Content (g)
Snack from list (see snack lists A & B)		

DINNER

65g lean pork OR<br>&	65g lean beef, chicken, ham or tofu or<br>2 grilled fish fingers	5
225g boiled rice OR<br>&	250g boiled, baked or mashed potato (made with margarine and milk from allowance)	1
60g onions OR<br>&	60g of one of the following:<br>courgettes, spinach, mixed vegetables, parsnips, cauliflower, Brussels sprouts, broccoli, carrots, swede, turnip or<br>½ 400g tin of tomatoes	0
60g mushrooms OR<br>&	60g of any of the following:<br>aubergine, cabbage, celery, leeks, marrow, pepper, tomatoes	0
15g vegetable oil OR<br>&	15g Sunflower, olive, safflower or corn oil<br>20g mayonnaise or margarine<br>30g double cream	15
Mixed Salad containing any of the following:<br>&	lettuce, cucumber, tomatoes, pickled beetroot, cabbage, carrots, celery, pickled gherkins, mushrooms, mustard and cress, peppers, radish, spring onion, watercress.	0
1 medium banana OR	2 medium apples or oranges or<br>200g grapes or<br>200g mandarins in juice or<br>200g pineapple in juice or<br>one tablespoon raisins.	0

EVENING SNACK

1 medium banana OR	2 medium apples or oranges or<br>200g grapes or	0

SOP for dietary assessment and intervention for public domain ©2001
Copyright © by the NUGENOB consortium
Neither this document nor any parts may be reproduced or transmitted in any form or by any means, electronic or mechanical, without permission in writing from the NUGENOB consortium.
Any use or quoting of this document requires notification of and accept from the NUGENOB consortium represented by Professor Thorkild IA Sorensen tias@ipm.hosp.dk

200g mandarins in juice or
200g pineapple in juice or
one tablespoon raisins

SOP for dietary assessment and intervention for public domain ©2001
Copyright © by the NUGENOB consortium
Neither this document nor any parts may be reproduced or transmitted in any form or by any means, electronic or mechanical, without permission in writing from the NUGENOB consortium.
Any use or quoting of this document requires notification of and accept from the NUGENOB consortium represented by Professor Thorkild IA Sorensen tias@ipm.hosp.dk

Low fat intervention template continued

		Approximate Fat Content (g)
**SNACKS TO BE CONSUMED OVER THE DAY** (1 from list A and 1 from list B)		
**Snack A**		
50g Madeira cake OR	Alternatives to be compiled	9
**Snack B**		
50g Hot cross bun with 20g jam OR	Alternatives to be compiled	3
**DAILY MILK ALLOWANCE**		
293g (½ pt/10 fl oz) semi skimmed milk OR	Alternatives to be compiled	5

SOP for dietary assessment and intervention for public domain ©2001
Copyright © by the NUGENOB consortium
Neither this document nor any parts may be reproduced or transmitted in any form or by any means, electronic or mechanical, without permission in writing from the NUGENOB consortium.
Any use or quoting of this document requires notification of and accept from the NUGENOB consortium represented by Professor Thorkild IA Sorensen tias@ipm.hosp.dk

APPENDIX IIIc

TEMPLATE
HIGH FAT INTERVENTION – 2000 KCALS

MACRONUTRIENT CONTRIBUTION

14% Protein, 45% Carbohydrate, 40% Fat

	ALTERNATIVES	Approximate Fat Content (g)
**BREAKFAST**		
3 Weetabix OR<br>&	60g Branflakes<br>Fruit'n Fibre (no oats) or<br>Shredded Wheat	2
150g orange juice OR	150g grapefruit juice<br>one medium orange or<br>one medium apple or<br>90g grapes or<br>100g pineapple in juice or<br>100g mandarin oranges in syrup	0
**MID-MORNING**		
Snack from list (see snack lists A, B & C)		
**LUNCH**		
Granary bread, 2 medium slices OR<br>&	2 medium slices of white, brown, wholemeal or Hovis bread or<br>75g french stick or<br>75g pitta bread (one small)	2
20g polyunsaturated margarine OR<br>&	20g mayonnaise or<br>50g reduced calorie mayonnaise or<br>50g salad cream	16
45g Cheddar cheese OR<br>&	1 large grilled sausage or<br>2 rashers streaky or back bacon grilled or<br>1 rasher middle bacon grilled or<br>2 eggs boiled or<br>55g tuna tinned in brine with 20g mayonnaise or<br>55g tinned salmon with 10g mayonnaise	15
Salad containing any of the following:	lettuce, cucumber, tomatoes, pickled beetroot, cabbage, carrots, celery, pickled gherkins,	0

SOP for dietary assessment and intervention for public domain ©2001
Copyright © by the NUGENOB consortium
Neither this document nor any parts may be reproduced or transmitted in any form or by any means, electronic or mechanical, without permission in writing from the NUGENOB consortium.
Any use or quoting of this document requires notification of and accept from the NUGENOB consortium represented by Professor Thorkild IA Sorensen tias@ipm.hosp.dk

mushrooms, mustard and cress, peppers,
radish, spring onion, watercress

SOP for dietary assessment and intervention for public domain ©2001
Copyright © by the NUGENOB consortium
Neither this document nor any parts may be reproduced or transmitted in any form or by any means, electronic or mechanical, without permission in writing from the NUGENOB consortium.
Any use or quoting of this document requires notification of and accept from the NUGENOB consortium represented by Professor Thorkild IA Sorensen tias@ipm.hosp.dk

High fat intervention template continued

	ALTERNATIVES	Approximate Fat Content (g)
**MID-AFTERNOON**		
Snack from list (see snack lists A, B & C)		
**DINNER**		
75g lean pork OR	75g lean beef, chicken, ham or tofu or<br>2 ½ grilled fish fingers	5
150g boiled rice OR	180g boiled, baked or mashed potato (with margarine and milk from allowance)	1
60g onions OR	60g of one of the following:<br>courgettes, spinach, mixed vegetables, parsnips, cauliflower, Brussels sprouts, broccoli or ½ 400g tin of tomatoes	0
60g mushrooms OR	60g of any of the following:<br>aubergine, cabbage, carrots, celery, leeks, marrow, pepper, swede, tomatoes, turnip	0
15g vegetable oil OR	15g sunflower oil, olive oil, safflower oil, corn oil<br>20g mayonnaise or margarine<br>30g double cream	15
Mixed Salad containing any of the following:	lettuce, cucumber, tomatoes, pickled beetroot, cabbage, carrots, celery, pickled gherkins, mushrooms, mustard and cress, peppers, radish, spring onion, watercress	0
1 medium banana OR	2 medium apples or oranges or<br>180g grapes or<br>200g mandarins in juice or<br>200g pineapple in juice or<br>one tablespoon raisins	1
**EVENING SNACK**		
Snack from list (see snack lists A, B & C)		

SOP for dietary assessment and intervention for public domain ©2001
Copyright © by the NUGENOB consortium
Neither this document nor any parts may be reproduced or transmitted in any form or by any means, electronic or mechanical, without permission in writing from the NUGENOB consortium.
Any use or quoting of this document requires notification of and accept from the NUGENOB consortium represented by Professor Thorkild IA Sorensen tias@ipm.hosp.dk

High fat intervention template continued

		Approximate Fat Content (g)
**SNACKS TO BE CONSUMED OVER THE DAY** (one from each list)		
**Snack A**		
50g Madeira cake OR	Alternatives to be compiled	8
**Snack B**		
30g bag of potato crisps	Alternatives to be compiled	11
**Snack C**		
30g fully coated chocolate biscuit	Alternatives to be compiled	8

DAILY MILK ALLOWANCE

350g (12 fl oz) semi skimmed milk	Alternatives to be compiled	4

SOP for dietary assessment and intervention for public domain ©2001
Copyright © by the NUGENOB consortium
Neither this document nor any parts may be reproduced or transmitted in any form or by any means, electronic or mechanical, without permission in writing from the NUGENOB consortium.
Any use or quoting of this document requires notification of and accept from the NUGENOB consortium represented by Professor Thorkild IA Sorensen tias@ipm.hosp.dk

APPENDIX IV

RECIPE SUGGESTIONS FOR EVENING MEAL (INDIVIDUAL PORTIONS USING TEMPLATE INGREDIENTS)

1. *Roast Dinner*
   Lean beef, chicken or pork with
   Boiled, baked or mashed potatoes
   15g margarine
   Selection of vegetables
   Instant gravy

2. *Stir fry*
   Lean beef, chicken, pork or quorn stir-fried in
   15g vegetable oil with
   Selection of vegetables and 2 teaspoons soy sauce
   Served with boiled rice

3. *Curry*
   Lean beef, chicken, pork or quorn
   15g vegetable oil
   Curry powder
   Mixed vegetables
   Served with boiled rice

4. *Spicy Beef*
   Lean beef or pork mince
   15g vegetable oil
   chilli powder
   Onion, peppers, mushrooms, tinned tomatoes and courgettes
   Served with boiled rice

5. *Sweet and Sour Pork*
   Lean pork diced
   Sauce including tinned pineapple, tomatoes, onions, vinegar, 15g oil
   Mixed vegetables
   Served with boiled rice

SOP for dietary assessment and intervention for public domain ©2001
Copyright © by the NUGENOB consortium
Neither this document nor any parts may be reproduced or transmitted in any form or by any means, electronic or mechanical, without permission in writing from the NUGENOB consortium.
Any use or quoting of this document requires notification of and accept from the NUGENOB consortium represented by Professor Thorkild IA Sorensen tias@ipm.hosp.dk

6. *Fish fingers and mash*
   Grilled fish fingers
   Potato mashed with 15g margarine
   Mixed vegetables

7. *Salad*
   Lean beef, chicken, pork or ham
   Boiled potatoes OR Rice salad
   Large mixed salad
   Reduced calorie mayonnaise

SOP for dietary assessment and intervention for public domain ©2001
Copyright © by the NUGENOB consortium
Neither this document nor any parts may be reproduced or transmitted in any form or by any means, electronic or mechanical, without permission in writing from the NUGENOB consortium.
Any use or quoting of this document requires notification of and accept from the NUGENOB consortium represented by Professor Thorkild IA Sorensen tias@ipm.hosp.dk

8. *Herby Cottage Pie*
   Lean minced beef
   15g oil for frying
   Onion, mushrooms, chopped tinned tomatoes, tomato puree
   Worcester sauce, yeast extract, mixed herbs
   Potatoes mashed with a little milk and fresh chopped basil and chives
   Serve with a mixed salad

9. *Lemon & Rosemary Steak or Chicken breast*
   Small beef steak
   Marinade in 15g olive oil, lemon juice, fresh rosemary black pepper and vinegar for 6-8 hours
   Barbeque or grill
   Serve with baked potato or rice and mixed salad

10. *Citrus chicken*
   Chicken breast
   Marinade in 5g olive oil, 15g lime juice, 60g orange juice, chopped chives and black pepper for at least 1 hour
   Cover with foil and bake
   Serve with a small portion of potatoes, margarine and vegetables or salad

11. *Oriental Pork*
   Lean pork, cut into slices
   Marinade in soy sauce, Worcestershire sauce, tomato ketchup, honey and mustard.
   Fry onion and meat (without marinate).
   Add milk and simmer until meat is cooked; remove and keep warm
   Add marinade, mushrooms and peppers to the sauce.
   Serve with boiled rice and small salad

12. *Stirfried Beef with Broccoli*
   Lean beef steak cut into strips
   15ml vegetable oil to fry
   Stir fry garlic then add beef
   Stir in 2 tsps oyster sauce, 2 tsps of fish sauce and a pinch of sugar
   Add 90g broccoli and stir fry
   Serve with boiled rice

13. *Chicken with Cumin Seeds*
   Fry onion and garlic in 15g vegetable oil
   Stir in 1 tsp turmeric, 2 tsps cumin seeds and sliced or cubed chicken breast
   Cook for 5 minutes before adding courgettes
   Serve with boiled rice and

SOP for dietary assessment and intervention for public domain ©2001
Copyright © by the NUGENOB consortium
Neither this document nor any parts may be reproduced or transmitted in any form or by any means, electronic or mechanical, without permission in writing from the NUGENOB consortium.
Any use or quoting of this document requires notification of and accept from the NUGENOB consortium represented by Professor Thorkild IA Sorensen tias@ipm.hosp.dk

Mixed salad

SOP for dietary assessment and intervention for public domain ©2001
Copyright © by the NUGENOB consortium
Neither this document nor any parts may be reproduced or transmitted in any form or by any means, electronic or mechanical, without permission in writing from the NUGENOB consortium.
Any use or quoting of this document requires notification of and accept from the NUGENOB consortium represented by Professor Thorkild IA Sorensen tias@ipm.hosp.dk

APPENDIX V

SOP FOR TAKING A DIET HISTORY

Rationale

The rationale for taking a diet history at the first dietary intervention meeting is as follows. A diet history is intended to give an overview of normal intake over a period of time longer than a 3-day weighed intake. The aim in this case is to develop a broader picture of the subject's eating habits than can be obtained from the 3- day weighed record, to pre-empt potential compliance problems and support the subjects' belief that the programme is tailored to their needs. It will be used to encourage the subject to think about areas where they will have to modify their intake and how they might do this.

A diet history is a retrospective dietary assessment methodology used to determine the usual eating and drinking pattern of the subject. Information regarding the range of foods eaten, their frequency, approximate quantities, and weekday/weekend variation over the recent past can be obtained. It is intended that the interview would be structured and include prompts and cross checks. Information obtained may be useful for research purposes but this is not the primary aim.

The Diet History

1. The subject will be sitting in comfortable surroundings in a private room. A partner may be present if desired.

2. The dietitian introduces themself and explains that the questions asked will help to determine the subject's usual eating and drinking pattern so that later advice can be tailored to their specific needs.

3. The subject should generally be considering items ingested over the last year, their frequency, seasonal variation and approximate quantity (in household measures).

4. The following background questions will be asked initially. (Some of the questions can be confirmed by looking at the completed 'background information' questionnaire):
   - Are you vegetarian?
   - Do you follow a special diet advised by a doctor or anyone else?
   - Are there any foods that you avoid?
   - How many are there in your household; does everyone eat together?
   - Who cooks and who does the shopping (ask which supermarket)?
   - What cooking equipment do you have at home? eg oven, microwave, grill, steamer?
   - Do you have a refrigerator/deep freeze?
   - Do you go out to work; how many days per week; what hours do you work; does this include shifts?

SOP for dietary assessment and intervention for public domain ©2001
Copyright © by the NUGENOB consortium
Neither this document nor any parts may be reproduced or transmitted in any form or by any means, electronic or mechanical, without permission in writing from the NUGENOB consortium.
Any use or quoting of this document requires notification of and accept from the NUGENOB consortium represented by Professor Thorkild IA Sorensen tias@ipm.hosp.dk

5. The diet history continues with the dietitian suggesting that the subject thinks about food and drink that they consumed yesterday. The following are suggested questions:
   - 5.1 What was the first thing that you ate or drank yesterday?
   - 5.2 Approximately what time was this?
   - 5.3 What exactly does this consist of (meal or item)?
   - 5.4 Do you usually eat or drink 'x' at this time and do you give this 'meal' a name?
   - 5.5 How much did you have (household measure) and did you have more than one portion?
   - 5.6 Did you have anything else with this?
   - 5.7 Are there any variations or other types of food or drink that you would eat at this time? – (prompt for weekday, weekend or work day/non-work day).
   - 5.8 How often do you have 'x' compared to 'y' per week? (prompt for daily, weekly, every 2 weeks, monthly)
   - 5.9 What was the next thing that you ate or drank? (repeat questions 5.1-5.9)

6. The dietitian then asks about specific foods from a checklist, using questions such as:
   - Do you eat/drink 'x'?
   - How often do you eat/drink this? Prompts – daily, weekly, every 2 weeks, monthly?
   - What types do you eat/what is your regular brand/what is your favourite/how do you cook this/when do you usually eat this?

7. A final question relevant to this study may be: "Are there any situations coming up in the next 3 months where you may not be able to follow your usual pattern of eating and the types of food you eat (eg parties, holidays etc)?"

SOP for dietary assessment and intervention for public domain ©2001
Copyright © by the NUGENOB consortium
Neither this document nor any parts may be reproduced or transmitted in any form or by any means, electronic or mechanical, without permission in writing from the NUGENOB consortium.
Any use or quoting of this document requires notification of and accept from the NUGENOB consortium represented by Professor Thorkild IA Sorensen tias@ipm.hosp.dk

SUGGESTED RECORDING SHEET FOR DIET HISTORY

Vegetarian?	Yes	No		
Special Diet?	Yes	No	If yes – what is this?	………………
Foods Avoided?	Yes	No	If yes – what are they?	………………

Cooking Facilities/Methods	Family	Work
Eg oven, grill, microwave<br>Deep freeze	How many eat together?	During day/How many days?<br>Shifts/Hours?<br>Staff canteen?

Time	Meal<br>(eg breakfast, snack etc)

SOP for dietary assessment and intervention for public domain ©2001
Copyright © by the NUGENOB consortium
Neither this document nor any parts may be reproduced or transmitted in any form or by any means, electronic or mechanical, without permission in writing from the NUGENOB consortium.
Any use or quoting of this document requires notification of and accept from the NUGENOB consortium represented by Professor Thorkild IA Sorensen tias@ipm.hosp.dk

SUGGESTED CHECKLIST FOR FREQUENCY OF FOODS COMMONLY CONSUMED
(This list should include foods containing guar gum and soluble fibre, and will need to be adjusted to local diet)

FOOD	FREQUENCY OF CONSUMPTION/ TYPES	FOOD	FREQUENCY OF CONSUMPTION/ TYPES
Bread		Fruit	
Spread		Vegetables	
Milk		Foods with pastry	
Cheese		Mayonnaise	
Yogurt		Frying	
Crisps		Meat	
Nuts		Fish	
Juices		Pulses	
Fizzy Pop		Alcohol	
Sugar		Eating out	
Cakes		Entertaining	
Biscuits		Forthcoming Events	
Sweets			
Chocolates		Total drinks daily	

SOP for dietary assessment and intervention for public domain ©2001
Copyright © by the NUGENOB consortium
Neither this document nor any parts may be reproduced or transmitted in any form or by any means, electronic or mechanical, without permission in writing from the NUGENOB consortium.
Any use or quoting of this document requires notification of and accept from the NUGENOB consortium represented by Professor Thorkild IA Sorensen tias@ipm.hosp.dk

References

1. Bingham S A, The dietary assessment of individuals; methods, accuracy, new techniques and recommendations. Nutrition Abstracts and Reviews (Series A). 1987 Vol. 57 No. 10.

2. Manual on Methodology for Food Consumption Studies edited by Cameron M E and Van Staveren W A. Oxford University Press 1988.

3. Human Nutrition and Dietetics 10th edition edited by Garrow J S, James W P T, Ralph A. Churchill Livingstone 2000.

4. Anderson A S, An overview of diet survey methodology. British Food Journal. 1995 Vol. 97 No. 7 pg22-26.

SOP for dietary assessment and intervention for public domain ©2001
Copyright © by the NUGENOB consortium
Neither this document nor any parts may be reproduced or transmitted in any form or by any means, electronic or mechanical, without permission in writing from the NUGENOB consortium.
Any use or quoting of this document requires notification of and accept from the NUGENOB consortium represented by Professor Thorkild IA Sorensen tias@ipm.hosp.dk

APPENDIX VI

SOP FOR SUBJECT INFORMATION LEADING UP TO THE CLINICAL INVESTIGATION DAY

WHAT TO DO ON THE THREE DAYS BEFORE THE STUDY DAY?

For the 3 days before your study day:

1. Do your normal amount of physical activity at work or at home but avoid any strenuous activity.
2. Drink the amount of alcohol you have usually unless it is more than recommended daily amounts (your centre will be able to advise you on this*).
3. Eat as you do normally

PRIOR TO THE STUDY MORNING

You must 'fast' for the 12 hours leading up to your clinical investigation morning. This means that from 8.00pm (2000hours) on the previous evening:

1. You are ALLOWED to drink unflavoured still bottle or tap water ONLY.
2. You must NOT eat or drink anything else.
3. You must NOT smoke.
4. You should continue to take your normal medication.

ON THE STUDY MORNING

Arrive at the study morning by car, public transport or taxi. We don't want you to be active on the study day (eg. by walking or cycling to the center).
As suggested in the 'Subject information sheet' it would be helpful to bring the following when you come for the morning:

1. Light clothing (Eg track suit bottoms or shorts with a T-shirt; women are advised not to wear a dress or 'one piece' underwear
2. Cassette tapes
3. Your completed 3-day Food Diary

* Centers are advised to use national guidelines.

SOP-Randomisation

Only ***obese subjects*** are to be randomised to the low- or high-fat intervention diet.

When the recruited obese subjects are visiting the centre for the first time approximately 10 days before the Clinical Investigation Day (WP4), information on age, height and weight should have been collected, and each subject should have been given a subject identification number.

After (or on) the clinical investigation day, i.e. in the period between the clinical investigation day and the first dietetic intervention vsit, the assistant co-ordinator Petra Lahmann, Institute of Preventive Medicine, Copenhagen, should be contacted in order to allocate the subject to the low- or high-fat diet. Note that the dietary intervention should start not later than 14 days after the clinical investigation day.

a. Call IPM Copenhagen: +45 33 38 37 60
b. Identify your self with your partner number, name of institution and country
c. You will first be asked about the gender and age in years of the subject
d. Then the subject number, height and weight should be given
e. The assistant co-ordinator will then decide if the subject shall have a low- or high-fat diet
f. Points c-e can be repeated if there are more subjects to be randomised

Be sure to carefully record this information in your own files. If there should be any doubts or reconsiderations, the assistant co-ordinator may be contacted again to clarify the decision.

SOP for Buffy Coat sampling.

The buffy coat is the small white layer between plasma and the red blood cells.
The buffy coat contains the white blood cells including the leucocytes, which contain DNA.

EDTA-tube:

Take the plasma out for the other samples.
Leave the phases close to the buffy coat.
With a small pipet, take the plasma above the buffy coat, the buffy coat itself and a small phase of the red blood cells and put this in the cryotube for the buffy coat (max 1 ml each cryotube). In that case, you are sure the vial contains the buffy coat.

Put the samples in the liquid nitrogen, store them in the -80 freezer

For further information on bloodsampling, centrifuging etc. see also the SOP on bloodsampling.

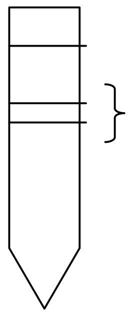

SOP Data entry and data transfer

Data entry in NUGENOB is based on a freeware program called EpiData. The program is developed for use on Windows based pc's. It may be possible to run it on a Mac with a pc-emulator program (not freeware), but we recommend that all clinical centres use a Windows pc.

Installation of EpiData

1. Point your Internet browser to www.epidata.dk.
2. Choose Program files and Documentation.
3. Download EpiData 2.0 by clicking on the Download button for EpiData 2.0 (1.3 MB).
4. Accept saving the file to disk by clicking ok.
5. Choose a directory to save the zip file named epid20.zip.

If you wish, you may download and view the Documentation. However, this SOP should supply you with the information needed.
From the main page you may register as a user of EpiData. We encourage you to do this to support the developers of this freeware program.

To install EpiData you need a zip program. If you do not know what a zip-file is, then view the explanation on the bottom of the Program files and Documentaion page of EpiData. We recommend that you use WinZip.

1. A free evaluation version of WinZip 8.0 can be downloaded from www.winzip.com (1.2 MB) by choosing Download evaluation version. This evaluation version is sufficient for the purposes of this project.
2. From the next page choose Download WinZip 8.0 for Windows 95/98/2000/ME.
3. From the next page choose Download now.
4. Accept saving the file to disk by clicking ok.
5. Choose a directory to save the zip file named winzip80.exe.
6. Double click winzip80.exe, which will start the installation.
7. During the setup accept all default values proposed.
8. When asked if you want to start with the WinZip Wizard or Classic setup choose Classic.

When WinZip is installed correctly:

1. Simply double-click on the epid20.zip file that you have downloaded. This will start WinZip and show the content of the archive.
2. Within WinZip double click on Setup.exe and follow the installation instructions. You may use all the default values proposed during the installation.
3. EpiData is started by choosing Programs/EpiData 2.0 from the start menu in the lower left corner of the Windows desktop. One may add a shortcut on the desktop by right clicking on Epidata 2.0 in the start menu, choosing copy, click on the desktop, right-clik and choose paste. Then EdiData can be started by double-clicking it on the desktop.

Preparing for data entry

All data should be entered on a single pc.
All data are collected in a single directory on the pc. Make a directory called c:\nugodata. If you for some reason choose to put data in another folder or drive please send an e-mail to ch@ipm.hosp.dk with this information.

Download the entire package of data entry form from the NUGENOB web-site to c:\nugodata. It is found under either WP12 at the bottom of the page or by pressing the button labelled data entry on the front page of the member section. The file is called allforms.zip. Double click it and chose save to file. Save it in c:\nugodata. Then double-click the zip-file and extract all files to c:\nugodata.
IF ANY DATA HAS BEEN ENTERED PREVIOUSLY THEY WILL DE OVERWRITTEN when unzipping the file. So do only do this once and then immediately delete the zip file allforms.zip.

Be sure that all files are in directory c:\nugodata

Entering data

Start EpiData. EpiData is started by choosing Programs/EpiData 2.0 from the start menu in the lower left corner of the Windows desktop, or by double-clicking the EpiData 2.0 icon on the desktop if available.

Choose *4. Enter Data* on the horizontal bar. From the open menu choose a file, for example the one for the questionnaire quest.rec, in the directory c:\nugodata. Be sure that the filetype is set to .rec. EpiData will jump to a new empty record when started.

In the lower left corner you can see the number of entered records and your current position in the data file. Use the triangles in the lower left corner to navigate from record to record.

Be sure that you are on a new empty record. Start entering data. Most people find it most convenient to use the numerical keypad on the right of the keyboard. Be sure that Num-Lock is turned on, otherwise the keypad will not produce numbers but instead produce cursor-movements.

The data entry form should be self-explanatory and closely follows the paper version of the questionnaire. When you enter a number that is as wide as the field (i.e. 3 digits in the subject number) the cursor will automatically jump to the next field. If the number is not as wide as the field you should press enter to jump to the next field.

Questions that are not answered in the questionnaire are simply left empty in the form by just pressing enter.

To jump forth and back between fields you can use the up and down arrow keys. You may also use the mouse to navigate the forms.

For each data entry form, which has the extension .rec, there is also a file called .chk. It contains all the rules on what should be entered in each field. This file should not be altered in any way by the

clinical centres. It is marked as read-only and should remain so. Also it is pertinent that it is not moved or deleted – otherwise your entered data are not validated.

QUESTIONNAIRE

The data entry form is named *quest.rec*

Questionnaires for subjects not eligible for enrolment should not be entered at present (but keep the paper version in your files).

After each page of the questionnaire two text fields for your notes have been added. In these fields you may write any supplementary information that might be relevant at a later stage.

Note that partner numbers below 10, e.g. 02, have to be entered as 2 without the zero

At certain places the entry program may skip some questions depending on what is entered. I.e., if the answer to question 11 is no (2) then question 11a and 12 will be skipped, and the program will jump to question 13. If the responder has answered question 11 and 12 anyway go back with the up-arrow key and fill out the fields.

For your convenience the many variables in questions 52b, 52c and the fields for extra siblings may be skipped if all are empty by answering yes (1) to the 'skip' question right before the variables.

CASE REPORT FORM

The Case Report Form for Obese has been divided into three parts:
Screening *scr_ob.rec*
Clinical Investigation Day *cid_ob.rec*
Dietary intervention and second
Clinical Investigation Day *dietintv.rec*

For the two first parts it is recommended to enter data for each subject, when each part is completed, i.e. after screening and after the Clinical Investigation Day. Some may even prefer to enter data from the Clinical Investigation Day right away as the investigation takes place.

For the dietary intervention part data (weight and compliance) should be entered after each visit.

The data entry program will automatically calculate all derived variables, e.g. mean weight, mean height, BMI, Waist/hip ratio. By also doing the calculations by hand on the paper version of the Case Report Form one can use these results to check that correct figures have been entered (or that one is able to use a calculator). Also the BMR on page 11 is automatically calculated according to the formulas.

Note that partner numbers below 10, e.g. 02, have to be entered as 2 without the zero

The Case Report Form for Reference Subjects has been divided into two parts:
Screening *scr_ref.rec*
Clinical Investigation Day *cid_ref.rec*

WEIGHED FOOD RECORDING

The results of the local calculations done on the weighed food records should be entered into this the form *foodrec.rec*,
Please examine the form to see which information is required.

The form is used for both obese and reference subject. Each single day is a separate record.

Data transfer to central database

At the end of each week data should be transferred to the central database in Copenhagen. This is done as follows:

1. Double-click the icon called bu.bat in c:\nugodata (If you are using Windows NT or Windows 2000 use bu2000.bat instead)
2. This will open a DOS window, that prompts for the date. Enter the date as ddmmyy
3. The program will now zip all your data files to a zip-file called buddmmyy.zip where ddmmyy is the date that was specified in 2. If you for example enter the date 270801 a file called bu270801.zip will be created.
4. Mail this file as an attachment to the address nugodata@ipm.hosp.dk
5. This should be done before Monday morning at 9.00, where it will be checked that all data have been received.

Each clinical centre may in addition choose to copy the zip-file to a floppy-disk and store it a safe place.

The zip-file is protected by a password (the same that is used to access the member section of the Nugenob-website) which must be specified if one wants to open the zip-file.
