# Supplementary material for: Genetic Polymorphisms and Weight Loss in Obesity: A Randomised Trial of Hypo-Energetic High- versus Low-Fat Diets
Source: PLoS Clin Trials. 2006 Jun 30;1(2):e12. doi: 10.1371/journal.pctr.0010012 (PMC1488899; doi:10.1371/journal.pctr.0010012)
Supplement: Alternative Language Abstract S1 [file pctr.0010012.sd004.doc]

**Abstract in Czech prepared by Vladimir Stich**

*Cíle:* Sledovat zdali geny s běžnými jednobodovými polymorfismy (SNPs), asociovanými s s fenotypy obesity, mají vliv na redukci váhy u obesních jedinců léčených nízkoenergetickou dietou s vysokým či nízkým podílem tuku.
*Design:* Randomizovaná, paralelní, dvouramenná, otevřená, multicentrická studie .
*Uspořádání:* Osm klinických středisek v sedmi evropských zemích

*Účastníci:* 771 obesních dospělých jedinců.
*Intervence:* 10-týdenní dietní intervence nízkoenergetickou (-600 kcal/d) dietou s energetickým podílem tuku 20-25 nebo 40-45%, ukončená u 648 jedinců.
*Výstupy*: Redukce váhy během 10 týdnů intervence ve vztahu ke genotypům 42 SNP u 26 kandidátských genů, pravděpodobně asociovaných s hypothalamickou regulací chuti k jídlu, účinností energetického výdeje, regulací diferenciace adipocytů, lipidovým a sacharidovým metabolismem, produkcí adipocytokinů, určena u 642 jedinců.
*Výsledky:* Pokud bylo pro každý ze zkoumaných SNP provedeno srovnání nositelů SNP a jedinců, kteří nebyli nositeli, pak, po adjustaci na pohlaví, věk, původní váhu a středisko, byla nalezena u heterozygotů rozdílná redukce váhy v rozmezí od –0.6 do 0.8 kg a u homozygotů v rozmezí od –0.7 do 3.1 kg. Dodatečná redukce váhy při dietě s nízkým podílem tuků byla v rozmezí od 1,.9 do –1.6 kg u heterozygotů a od 3.8 kg dto –2.1 kg u homozygotů ve srovnání s jedinci, kteří nebyly nositeli daného SNP. Při mnohočetném testování se neukázala žádná z asociací jako statisticky významná.

*Závěr:* Polymorfismy kandidátských genů spojených s obesitou mají malou, a snad zanedbatelnou, roli v regulaci změn váhy navozených nízkoenergetickou dietou s vysokým či nízkým podílem tuku
