# Supplementary material for: Genetic Polymorphisms and Weight Loss in Obesity: A Randomised Trial of Hypo-Energetic High- versus Low-Fat Diets
Source: PLoS Clin Trials. 2006 Jun 30;1(2):e12. doi: 10.1371/journal.pctr.0010012 (PMC1488899; doi:10.1371/journal.pctr.0010012)
Supplement: Alternative Language Abstract S2 [file pctr.0010012.sd005.doc]

**Abstract in Danish prepared by Thorkild IA Sørensen (with help by Katrine Grau)**

*Formål:* At undersøge om gener med hyppigt forekommende enkelt-nukleotid-polymorfismer (SNPs), som er associeret med fedmerelaterede fænotyper, påvirker vægttab hos fede forsøgsdeltagere, der behandles med en lav-kalorisk diæt med lavt eller højt fedtindhold.

*Design:* Randomiseret, parallelt, to-armet, ublindet, multicenter forsøg.

*Lokalisering:* Otte kliniske centre i syv europæiske lande.

*Deltagere:* 771 fede, voksne forsøgsdeltagere.

*Intervention:* 10-ugers kostintervention til lav-kaloriske (-600 kcal/d) diæter med tilstræbt fedtenergiprocent på 20-25 eller 40-45, gennemført i 648 forsøgsdeltagere.

*Udfald:* Vægttab gennem de ti uger i forhold til genotyperne af 42 SNPs i 26 kandidatgener, som sandsynligvis er associeret med hypothalamisk appetitregulation, efficiens af energiforbrug, regulering af fedtcelle-differentiering og -funktion, lipid- og glukosemetabolisme eller produktion af adipocytokiner, bestemt i 642 forsøgsdeltagere.

*Resultater:* Sammenlignet med ikke-bærere af hver af SNP’erne, og efter justering for køn, alder, baseline-vægt og center, viste heterozygote vægttabsdifferencer fra –0.6 til 0.8 kg, og homozygote fra –0.7 til 3.1 kg. Genotypeafhængigt, yderligere vægttab på diæten med lavt fedtindhold var fra 1.9 til –1.6 kg hos heterozygote og fra 3.8 kg til –2.1 kg hos homozygote, sammenlignet med ikke-bærere. Når de multiple test tages i betragtning, var ingen af associationerne statistisk signifikante.

*Konklusion:* Polymorfismer i en vifte af fedmerelaterede kandidatgener spiller en begrænset rolle, om nogen overhovedet, i forhold til vægtændringer fremkaldt ved en moderat lav-kalorisk diæt med højt eller lavt fedtidhold.
