# Supplementary material for: Genetic Polymorphisms and Weight Loss in Obesity: A Randomised Trial of Hypo-Energetic High- versus Low-Fat Diets
Source: PLoS Clin Trials. 2006 Jun 30;1(2):e12. doi: 10.1371/journal.pctr.0010012 (PMC1488899; doi:10.1371/journal.pctr.0010012)
Supplement: Alternative Language Abstract S3 [file pctr.0010012.sd006.doc]

**Abstract in Dutch prepared by Wim HM Saris**

*Doelstellingen:* Onderzoek of genen met enkelvoudige nucleotide polymorphisms (SNPs) gerelateerd zijn aan obesitas fenotypes welke het gewichts verlies beinvloeden in obese personen behandeld met een matig hypo-energetisch laag-vet of hoog vet diet.

*Studie opzet:* Gerandomiseerd, parallel, twee-armig, open label multi-centre onderzoek.

*Setting:* Acht klinische centra in zeven Europese landen.

*Deelnemers:* 771 obese mannen en vrouwen.

*Interventie:* 10-weken diet interventie met een hypo-energetisch (-600 kcal/d) diet met een beoogd vet energie percentage van 20-25 of 40-45, succesvol beeindigd door 648 personen.

*Uitkomsten:* Gewichtsverlies gedurende de 10 weken in relatie tot genotypen van 42 SNPs in 26 kandidaat genen, mogelijjk geassocieerd met de hypothalame regulatie van honger en verzadiging, efficientie van het energie gebruik, regulatie van vetcel differentiatie and functie van het lipiden en glucose metabolisme of productie van adipocytokines, gemeten in 642 personen.

*Resultaten:* Voor elk van de SNP’s gemeten ten opzichte van de niet-dragers, en na correctie voor sex, leeftijd, startgewicht en centra laten heterozygoten een gewichtsverlies zien dat varieert van –0.6 tot 0.8 kg, en homozygoten, van –0.7 tot 3.1 kg. Genotypische afhankelijk additioneel gewichtsverlies op een laag vet diet varieert van1.9 tot –1.6 kg in heterozygoten, en van 3.8 kg tot –2.1 kg in homozygoten ten opzichte van niet-dragers. Gegeven het multipele testen was geen van de associaties statistisch significant.

*Conclusie:* Polymorphisme in een panel van obesitas-gerelateerde kandidaat genen spelen slechts een kleine of mogelijk geen rol van betekenis in het moduleren van gewichts veranderingen onder invloed van een matig hypo-energetisch laag vet of hoog vet dieet.
