# Supplementary material for: Genetic Polymorphisms and Weight Loss in Obesity: A Randomised Trial of Hypo-Energetic High- versus Low-Fat Diets
Source: PLoS Clin Trials. 2006 Jun 30;1(2):e12. doi: 10.1371/journal.pctr.0010012 (PMC1488899; doi:10.1371/journal.pctr.0010012)
Supplement: Alternative Language Abstract S4 [file pctr.0010012.sd007.doc]

**Abstract in French prepared by Jean-Michel Oppert**

*Objectifs:* Déterminer si des gènes avec des variants (single nucleotide polymorphisms, SNPs) communs associés à des phénotypes d’obésité peuvent influencer la perte de poids chez des sujets obèses suivant un régime hypocalorique pauvre ou riche en graisses.
*Protocole:* Essai randomisé, multicentrique, en ouvert, avec deux bras parallèles.
*Sites:* Huit centres cliniques dans sept pays européens.

*Participants:* 771 adultes obèses.

*Interventions:* Intervention diététique pendant 10 semaines avec régime hypocalorique (-600 kcal/jour) dont l’apport lipidique était ciblé à 20-25% ou 40-45%, 648 sujets ayant suivi l’essai jusqu’à son terme.

*Critères de jugement:* Perte de poids après 10 semaines en fonction des génotypes correspondant à 42 SNPs situés dans 26 gènes-candidats impliqués le contrôle hypothalamique de la prise alimentaire, le rendement énergétique, le contrôle de la différenciation et du métabolisme adipocytaires, les métabolismes lipidiques et glucidiques ou la production d’adipocytokines, déterminés chez 642 sujets.

*Résultats:* Pour chaque SNP, après ajustement sur le sexe, l’âge, le poids initial et le centre, la différence de perte de poids par comparaison avec les sujets non-porteurs était comprise entre -0,6 et 0,8 kg chez les sujets hétérozygotes et entre -0,7 et 3,1 kg chez les sujets homozygotes. Par comparaison avec les sujets non porteurs, la perte de poids additionnelle liée au génotype sous le régime pauvre en graisses était comprise entre 1,9 et -1,6 kg chez les hétérozygotes et entre 3,8 et -2,1 kg chez les homozygotes. Aucune de ces associations n’était significative si l’on prenait en compte le nombre de tests effectués.

*Conclusion:* Ces polymorphismes dans un panel de gènes-candidats associés à l’obésité ne semblent jouer, au plus, qu’un rôle mineur pour moduler les variations de poids sous régime hypocalorique modéré à contenu élevé ou faible en graisses.
