# Supplementary material for: Genetic Polymorphisms and Weight Loss in Obesity: A Randomised Trial of Hypo-Energetic High- versus Low-Fat Diets
Source: PLoS Clin Trials. 2006 Jun 30;1(2):e12. doi: 10.1371/journal.pctr.0010012 (PMC1488899; doi:10.1371/journal.pctr.0010012)
Supplement: Alternative Language Abstract S5 [file pctr.0010012.sd008.doc]

**Abstract in Spanish prepared by J. Alfredo Martinez**

*Objetivos*: Investigar si algunos polimorfismos genéticos, que afectan a un solo nucleotido (SNP) y potencialmente asociados a fenotipos de obesidad, influyen sobre la perdida de peso en sujetos obesos tratados con una dieta hipocalórica baja en grasa.

*Diseño*: Ensayo abierto multicentrico, aleatorizado y paralelo con dos brazos.

*Localización:* 8 centros clínicos de 7 países europeos.

*Participantes:* 771 individuos obesos.

*Intervención:* Seguimiento de una dieta hipo-energética (-600 Kcal/día) durante 10 semanas con niveles de grasa preestablecidos en 20-25% y 40-45%, que fue completado por 648 participantes.

*Desenlace variables:* Perdida de peso al cabo de 10 semanas en portadores de 42 diferentes SNP correspondientes a 26 genes relacionados potencialmente con la regulación hipotalámica del apetito, eficiencia energética, regulación de la diferenciación y función de adipocitos, metabolismo hidrocarbonado o lipídico y con la producción de adipoquinas en 642 individuos.

*Resultados:* Las diferencias en la perdida de peso oscilaron entre -0,6 y 0,8 Kg, de portadores heterocigotos en relación a no portadores de los polimorfismos estudiados después de ajustar por sexo, edad, peso inicial y centro. Los portadores homocigotos mostraron una diferencia que variaba entre -0,7 Kg y 3,7 Kg en relación a no portadores de la variante genética correspondiente. Las perdidas de peso dependientes del genotipo en individuos que recibieron la dieta baja en grasa defirieron entre 1,9 y 1,6 Kg en los heterocigotos y entre 3,8 y 2,1 Kg en los homocigotos en comparación con los no portadores del polimorfismo considerado. Debido a las comparaciones estadísticas múltiples, la posible asociación no fue considerada estadísticamente significativa.

*Conclusión:* Los polimorfismos en un panel de genes candidatos parecen tener un papel poco importante en la perdida de peso dependiente de la prescripción de dietas hipocalóricas con diferente contenido en grasa.
