# Supplementary material for: Genetic Polymorphisms and Weight Loss in Obesity: A Randomised Trial of Hypo-Energetic High- versus Low-Fat Diets
Source: PLoS Clin Trials. 2006 Jun 30;1(2):e12. doi: 10.1371/journal.pctr.0010012 (PMC1488899; doi:10.1371/journal.pctr.0010012)
Supplement: Alternative Language Abstract S6 [file pctr.0010012.sd009.doc]

**Abstract in Swedish prepared by Peter Arner**

*Ändamål:* Att studera om gener med vanliga strukturella variationer (SNPs) som är kopplade till fetma-relaterade fenotyper kan påverka viktminskning hos feta individer vilka behandlas med en lågkalorisk diet innehållande lite fett eller mycket fett.

*Utförande:* Undersökning från flera centra med slumpartad lottning till två undersökningsarmar som studerades samtidigt.

*Deltagande:* 8 kliniska centra i 7 europeiska länder.

*Studiegrupp:* 771 feta, vuxna personer.

*Invention:* 10 veckors diet som var måttligt lågkalorisk (-600 kcal/dag) med ett förväntat fettinnehåll på antingen 20-25% eller 40-45%. 648 personer genomförde studien.

*Mätvariabler:* Viktminskning under 10 veckors diet satt i relation till genetisk variation (42 SNPs) i 26 kandidatgener för fetma. Dessa gener kan tänkas associera med hypotalamisk reglering av aptit, energiomsättning, reglering av fettcellens utmognad och funktion, lipid- och glukosmetabolism eller produktion av adipocytokiner, vilket bestämdes hos 672 individer.

*Resultat:* Jämfört med individer som inte hade fetma-associerade SNPs och efter korrektion för kön, ålder, initial vikt och forskningscenter så visade heterozygoter skillnader i viktförlust under dieten som varierade mellan 0.6-0.8 kg. För homozygoter var skillnaden 0.7-3.1 kg. Hos de som fick lågfettsdiet var dessa skillnader 1.9 - 1.6 kg hos heterozygoter och 3.8 - 2.1 kg hos homozygoter. Inga av de beskrivna skillnaderna var statistiskt signfikanta när inflytande av multipel testning vägdes in.

*Konklusion:* Polymorfi i en panel av kandidatgener för fetma spelar liten eller ingen roll för den viktminskning man ser efter lågfett- eller högfettsdiet med måttligt minskat energiinnehåll.
